# Global, Regional, and National Burden of Malaria and Dengue from 1992 to 2021, with Projections to 2036: An Age–Period–Cohort Analysis

**DOI:** 10.3390/tropicalmed11070201

**Published:** 2026-07-17

**Authors:** Yu Wang, Qilan Wen, Jinwei Chen, Yikun Chang, Na Cao, Xin Sun, Yuantao Hao, Wangjian Zhang, Zhicheng Du

**Affiliations:** 1 Department of Medical Statistics, School of Public Health, Center for Health Information Research, Sun Yat-sen Global Health Institute, Sun Yat-sen University, Guangzhou 510080, China; wangy2677@mail2.sysu.edu.cn (Y.W.); wenqlan@mail2.sysu.edu.cn (Q.W.);; 2 Center for Public Health and Epidemic Preparedness & Response, Peking University, Beijing 100191, China; 3 Department of Epidemiology & Biostatistics, School of Public Health, Peking University, Beijing 100191, China; 4 Key Laboratory of Epidemiology of Major Diseases, Ministry of Education, Peking University, Beijing 100191, China; 5 Guangzhou Joint Research Center for Disease Surveillance and Risk Assessment, Sun Yat-sen University & Guangzhou Center for Disease Control and Prevention, Guangzhou 510440, China

**Keywords:** malaria, dengue, global burden of disease, incidence, disability-adjusted life years, health inequalities

## Abstract

This study aimed to compare the burden of malaria and dengue worldwide and to project future incidence trends. Incidence and disability-adjusted life-years (DALYs) were obtained from the Global Burden of Disease Study 2021 (GBD 2021). Temporal trends were quantified using estimated annual percentage change (EAPC), age–period–cohort analysis was used to assess age, period, and cohort effects, and Bayesian age–period–cohort models were applied to project incidence to 2036. From 1992 to 2021, the global age-standardized incidence rate (ASIR) of malaria decreased (EAPC= −0.55%), whereas that of dengue increased (EAPC = 1.83%); the corresponding age-standardized DALY rates showed EAPCs of −1.64% and 1.29%, respectively. Across 21 GBD regions, SDI was inversely correlated with ASIR for malaria (*r* = −0.848) and dengue (*r* = −0.521), and with age-standardized DALY rates for malaria (*r* = −0.849) and dengue (*r* = −0.497). Malaria burden remained concentrated in low-SDI regions, particularly sub-Saharan Africa and Oceania, whereas dengue burden was highest in middle-SDI regions, especially tropical Latin America. From 2021 to 2036, the global ASIR of malaria was projected to decrease slightly by 1.36% (3485.27 to 3437.72). Whereas that of dengue was projected to increase by 10.58% (752.04 to 831.63). In males, the projected ASIR of malaria and dengue increased by 4.18% (3193.24 to 3326.58) and 9.96% (704.83 to 775.06), respectively. In females, the corresponding increases were 4.87% (3379.82 to 3544.27) and 8.83% (819.74 to 892.13). These findings suggest divergent epidemiological trajectories for malaria and dengue. The projections further suggest a consistent upward trajectory for dengue, with sex-specific relative changes differing descriptively by disease: the projected relative increase was numerically greater in females for malaria and in males for dengue.

## 1. Introduction

Malaria and dengue are the two most important mosquito-borne diseases worldwide and remain major causes of morbidity, mortality, and economic loss [1]. Although both are transmitted by mosquitoes, they differ substantially in pathogen type, epidemiology, affected populations, and control challenges. Malaria is a parasitic infection transmitted by *Anopheles* mosquitoes, whereas dengue is a viral disease transmitted mainly by *Aedes aegypti* and *Aedes albopictus*. Both diseases have shown a clear trend of expansion into previously unaffected regions, as well as re-emergence in areas where transmission had declined [2].

The burden of both diseases remains considerable. In 2023, an estimated 263 million malaria cases and 597,000 deaths were reported across 83 countries, with the African Region accounting for 94% of cases and 95% of deaths; children younger than 5 years remain the most vulnerable group [3]. Dengue has become the fastest-growing mosquito-borne viral disease globally [4] and was identified by the World Health Organization (WHO) as one of the ten major global public health threats in 2019 [5]. Since early 2023, dengue activity has increased sharply, with more than five million cases and approximately 5000 deaths reported across all WHO regions, and by 2024 active transmission had been documented in nearly 90 countries [6]. The health impact of both diseases is compounded by persistent gaps in prevention and treatment, including growing antimalarial drug resistance and the absence of specific antiviral therapies for dengue [6,7,8,9]. Their economic consequences are also substantial: dengue was estimated to impose an annual global cost of US$8.9 billion in 2013, while malaria caused US$4.3 billion in direct and indirect losses in 2016 [10,11].

Existing evidence has improved understanding of malaria and dengue, but most studies have examined them separately and with different analytical emphases. Recent work has characterized malaria incidence trends in high-risk regions using age–period–cohort (APC) analysis and has described malaria burden in children and young adolescents at global, regional, and national levels [12,13]. Parallel studies have quantified the global dengue burden using GBD 2021 data, including disease patterns in children and adolescents and APC effects in high-risk regions [14,15,16]. These studies have clarified important disease-specific patterns, but they do not provide a directly comparable account of how malaria and dengue differ in their long-term epidemiological trajectories, demographic profiles, and geographic concentration under a unified framework [12,13,14,15,16].

This gap matters for public health planning because malaria and dengue are increasingly competing for surveillance capacity, prevention resources, and policy attention across many settings. Evidence remains limited on their comparative burden over time, on the extent to which age, period, and cohort effects differ between them, and on how future incidence may evolve if recent trajectories persist. These limitations also constrain assessment of whether current progress is sufficient to support the WHO’s 2030 Sustainable Development Goals (SDGs) related to vector-borne disease control. Broader forecasting analyses of neglected tropical diseases and malaria have been reported, but these do not resolve the specific comparative epidemiology of malaria versus dengue [17]. We therefore conducted a comparative analysis of malaria and dengue burden from 1992 to 2021 using data from the Global Burden of Disease Study 2021, assessing incidence and disability-adjusted life years (DALYs) at global, regional, and national levels, examining variation across Socio-demographic Index (SDI) settings, applying APC modeling to characterize temporal patterns, and using Bayesian age–period–cohort (BAPC) modeling to project incidence trends from 2021 to 2036.

## 2. Methods

### 2.1. Data Sources

Data on incident counts, DALY counts, age-standardized rates (ASR) of incidence and DALYs for malaria and dengue from 1992 to 2021 were obtained from the Global Burden of Disease Study 2021 (GBD 2021). All data are freely accessible through the Global Health Data Exchange (https://ghdx.healthdata.org/gbd-2021/sources, accessed on 18 November 2024). ASR were computed per 100,000 population using the GBD reference population. The primary analyses used the GBD mean point estimates. The 95% uncertainty interval (UI) reported by GBD represents the 2.5th and 97.5th percentiles of the posterior draws for each estimate. All descriptive analyses reported 95% UI and the lower and upper GBD estimates were used in APC sensitivity analyses. Countries and territories (*n* = 204) were classified into five SDI quintiles (low, low-middle, middle, high-middle, and high). SDI is a composite measure of income per capita, educational attainment, and total fertility rate.

### 2.2. Statistical Analysis

#### 2.2.1. Temporal Trend Analysis

Temporal trends in ASR were quantified using the estimated annual percentage change (EAPC), a well-established metric for evaluating disease trajectory over time. We fitted a linear regression model to the natural logarithm of the ASR to capture the exponential nature of disease rate changes.

y =α +βx +ε

where *y* = ln (ASR), *x* = calendar year, *α* represents the intercept, *β* denotes the regression coefficient indicating the rate of change per year, and *ε* is the error term. The EAPC was subsequently computed as $100\;\times \;(e^{\beta }\;-\;1)$, representing the average annual percentage change in ASR over the study period [18]. An increasing trend was defined as an EAPC with a lower limit of the 95% confidence interval (CI) greater than 0, a decreasing trend as an upper limit less than 0, and a stable trend otherwise [19]. We further conducted descriptive analyses to summarize temporal patterns in incidence and DALYs across different demographic strata, including 5-year age groups, SDI regions, and the 21 GBD geographical areas, enabling the identification of high-risk populations and geographical hots pots.

To describe nonlinear global trends, we additionally performed joinpoint regression for malaria and dengue ASIR and age-standardized DALY rates from 1992 to 2021. Segmented log-linear models were used to estimate annual percentage change (*APC*) for each segment and average annual percentage change (*AAPC*) across the whole period. *APC* and *AAPC* with 95%CI were reported.

#### 2.2.2. Correlation Analysis

Associations between ASR and the SDI were evaluated using locally estimated scatterplot smoothing (LOESS) and Spearman’s rank correlation [15]. LOESS curves were fitted using the “geom_smooth()” function from the “ggplot2” package in R to visualize nonlinear relationships between malaria or dengue ASR and SDI across 21 GBD regions for 1992 and 2021. Spearman’s correlation quantified the strength (*r*) and significance of these associations.

#### 2.2.3. Age–Period–Cohort Analysis

Age-specific incident counts and corresponding population estimates were categorized into twenty consecutive 5-year age groups and six 5-year calendar periods from 1992 to 2021, as required by the National Cancer Institute APC Web Tool (https://analysistools.cancer.gov/apc/, accessed on 15 March 2025). We used GBD incident case counts as the numerator and population estimates as the person-year denominator. The Poisson log-linear APC model was specified as:

$$Y_{ap}=Poisson\left(\mu_{ap}\right)$$

$$\ln\left(\mu_{ap}\right)=\ln\left(P_{ap}\right)+\alpha_{a}+\beta_{b}+\gamma_{c}$$

where $Y_{ap}$ is the number of incident cases in age group *a* and period *p*, $P_{ap}$ is the corresponding population offset, $\alpha_{a}$ is the age effect, $\beta_{b}$ is the period effect, and $\gamma_{c}$ is the cohort effect defined by c=p−a. Net drift (overall temporal trend) and local drifts (age-specific trends) were estimated as annual percentage changes. In the APC model, the age effect was represented by age-specific rates aligned with birth cohorts, while period and cohort effects were modeled as relative risks [20]. The reference groups were set as the 0–4 age group, the 2002–2006 period, and the 1957–1961 birth cohort for age, period, and cohort effects, respectively. Rate ratios (*RR*s) with 95%CI were calculated to compare each category against its reference. The Wald χ^2^ test was used to assess statistical significance [21]. To assess the influence of GBD uncertainty on APC conclusions, we repeated the APC analyses using the lower and upper bounds of the GBD 95% UI as sensitivity analyses. Results were visualized using R version 4.4.2.

#### 2.2.4. Bayesian Age–Period–Cohort Projection

We implemented a BAPC model to project incidence rates through 2036. This model has been verified to outperform many other linear power models and can achieve more reasonable predictions [22]. The models used incident counts with population offsets and assumed a Poisson likelihood:

$$Y_{i}\sim \;Poisson\left(\mu_{i}\right)$$

$$ln\left(\mu_{i}\right)=ln\left(P_{i}\right)+\eta_{i}$$

where $P_{i}$ denotes the population offset and $\eta_{i}$ includes age, period, cohort, and overdispersion components. Posterior inference was performed using integrated nested Laplace approximation (INLA), as implemented in the BAPC and INLA packages in R [23]. Candidate models used first- or second-order random-walk priors (RW1 or RW2) for age, period, and cohort effects, with log-gamma precision hyperpriors, consistent with common BAPC/INLA implementations [22]. Model selection considered both in-sample fit and out-of-sample predictive performance. We trained candidate models on 1992–2011 data and tested them against observed GBD estimates for 2012–2021. Deviance Information Criterion (*DIC*) was used to assess model fit [24], whereas Root Mean Squared Error (*RMSE*), Mean Absolute Error (*MAE*), and Mean Absolute Percentage Error (*MAPE*) were used to quantify predictive error. The final projection model was selected by prioritizing out-of-sample predictive performance among models with acceptable *DIC* and numerical stability. Projected ASIR were summarized as posterior means with 95%CI. Future population denominators were based on GBD population projections.

## 3. Results

### 3.1. Global and SDI Regional Trends

**Figure 1:**
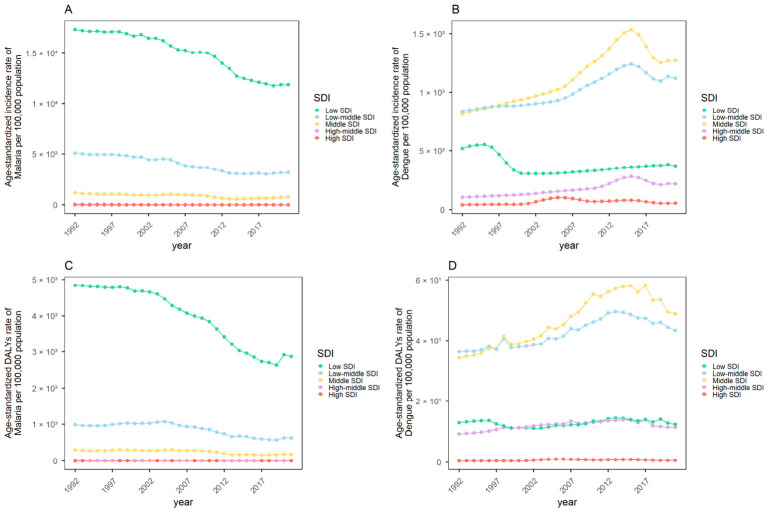
ASR of incidence and DALYs contributed by SDI groups, for malaria and dengue, in 1992–2021. ASIR (**A**) and ASR of DALYs (**C**) of malaria. ASIR (**B**) and ASR of DALYs (**D**) of dengue. DALYs = disability-adjusted life-years; ASR = age-standardized rate; SDI = sociodemographic index.

As shown in Tables S1 and S2, malaria cases increased from 221.029 million in 1992 to 249.117 million in 2021, whereas malaria ASIR decreased from 3678.17 to 3485.27 per 100,000 (EAPC = −0.55%) and the ASR of DALYs decreased from 993.67 to 806.00 per 100,000 (EAPC = −1.64%). Dengue cases increased from 28.847 million to 58.964 million, the ASIR of dengue was 752.04 per 100,000 in 2021 and the ASR of DALYs increased from 22.06 to 27.76 per 100,000 (EAPC = 1.29%). Malaria burden remained substantially higher than dengue burden (Figure 1 and Figure S1).

Joinpoint regression showed nonlinear global trends (Table S8). Malaria ASIR increased during 1992–2001 (APC = 0.93%, 95%CI 0.68 to 1.19), declined during 2001–2010 and 2010–2014, and increased again during 2014–2021 (APC = 0.95%, 95%CI 0.52 to 1.39); the overall *AAPC* was −0.21% (95%CI −0.42 to 0.00). The malaria ASR of DALYs showed a significant overall decline (*AAPC* = −0.63%, 95%CI −0.97 to −0.29), with an increase during 2018–2021. Dengue ASIR increased during 1992–2004 and 2004–2015, declined during 2015–2018, and then stabilized during 2018–2021; the overall *AAPC* was 1.31% (95%CI 1.07 to 1.55). The dengue ASR of DALYs increased overall (*AAPC* = 0.82%, 95%CI 0.52 to 1.12), despite a decline during 2014–2021.

Across SDI regions, malaria burden was concentrated in low SDI and low-middle SDI regions, whereas dengue burden was highest in low-middle SDI and middle SDI regions (Figure 1). The largest decline in malaria rates was observed in high SDI regions, with EAPCs of −15.02% (95%CI −16.87% to −13.12%) for ASIR and −10.90% (95%CI −11.97% to −9.82%) for the ASR of DALYs. For dengue, the highest increase in ASIR occurred in middle-high SDI regions (EAPC 3.43%, 95%CI 2.97% to 3.90%), whereas the largest increase in the ASR of DALYs occurred in middle SDI regions (EAPC 1.86%, 95%CI 1.53% to 2.19%) (Tables S1 and S2).

Regional heterogeneity was marked (Table S3). For malaria, Western sub-Saharan Africa had the largest number of incident cases in 2021 (137.21 million), while Central sub-Saharan Africa had the highest ASIR (21,152.56 per 100,000) and Western sub-Saharan Africa had the highest ASR of DALYs (5668.41 per 100,000). The most rapid declines in malaria ASIR and ASR of DALYs were observed in Central Asia (EAPC −34.05%, 95%CI −44.07% to −22.24% and −22.77%, 95%CI −33.70% to −21.31%). For dengue, the highest ASIR in 2021 occurred in Tropical Latin America (5774.82 per 100,000), followed by South Asia, Central Latin America, and Southeast Asia. The highest ASR of DALYs occurred in Southeast Asia (147.04 per 100,000), followed by Tropical Latin America and South Asia. High-income North America had the largest relative EAPC for dengue ASIR and DALY rates, but its absolute ASIR remained very low, increasing from 0.09 per 100,000 in 1992 to 0.36 per 100,000 in 2021.

Overall, malaria burden remained overwhelmingly concentrated in low SDI regions, particularly sub-Saharan Africa, with broadly declining trends across most endemic areas, whereas dengue burden was highest and fastest growing in middle-higher SDI regions, especially in Latin America and parts of Asia.

### 3.2. National Trends

At the national level, 83 countries and territories reported malaria cases and 85 reported malaria DALYs in 2021 (Tables S4 and S6). Nigeria had the largest number of cases (71,275,230, 28.61% of the global total), followed by the Democratic Republic of the Congo and Uganda. Together these three countries accounted for 43.47% of global cases. The highest malaria ASIRs were observed in Liberia (27,702.66 per 100,000), Benin, and Burkina Faso. Nigeria also had the highest number of malaria DALYs (16,497,677.19), and Sierra Leone had the highest malaria ASR of DALYs (8940.31 per 100,000). Among endemic countries, 72 of 85 showed declining malaria ASR of DALYs. EAPCs in Tables S4–S7 were estimated from all available annual GBD observations for each country. The largest reductions were observed in Bhutan (EAPC = −27.08%), Ecuador (EAPC = −22.60%), and Guatemala (EAPC = −20.73%) (Table S6, Figure S3), whereas Venezuela showed increases in both malaria ASIR and ASR of DALYs (Tables S4 and S6, Figures S2 and S3).

Dengue was reported in 126 countries and territories in 2021, with 134 reporting dengue DALYs (Tables S5 and S7). India had the largest number of dengue cases (28,205,519, 47.84% of the global total), followed by Brazil and Pakistan; together these three countries accounted for 74.11% of global cases. The highest dengue ASIRs were observed in Tonga (14,363.29 per 100,000), Seychelles, and Comoros. India also had the highest number of dengue DALYs (841,615.98), whereas Tonga had the highest ASR of DALYs (168.65 per 100,000). Among countries with data, 74 of 134 showed increasing dengue ASR of DALYs. The largest increases were observed in Equatorial Guinea, Nauru, and Guatemala.

Geospatially, malaria remained highly endemic in tropical Africa, whereas dengue is increasingly widespread, with high transmission intensity in Southeast Asia and South America and expansion toward temperate zones, including parts of North America and Oceania (Figures S2–S5).

### 3.3. Age Patterns

In 2021, malaria burden was concentrated in children younger than 5 years and overall in people younger than 24 years. Dengue showed a broader age distribution, with elevated incidence in those aged 5–24 years and in adults aged 90 years or older; the highest dengue ASR of DALYs occurred in children younger than 9 years and adults aged 85 years or older.

From 1992 to 2021, malaria ASIR and ASR of DALYs decreased across age groups, most markedly in adults aged 60 years or older, whereas the malaria decline was smaller in those aged 25–34 years. Dengue incidence and ASR of DALYs increased in all age groups, with the largest increase in older adults and the fastest rise in ASIR in those aged 95 years or older (EAPC = 3.29%) (Table 1).

### 3.4. Correlation Between SDI and the Burden of Malaria and Dengue

**Figure 2:**
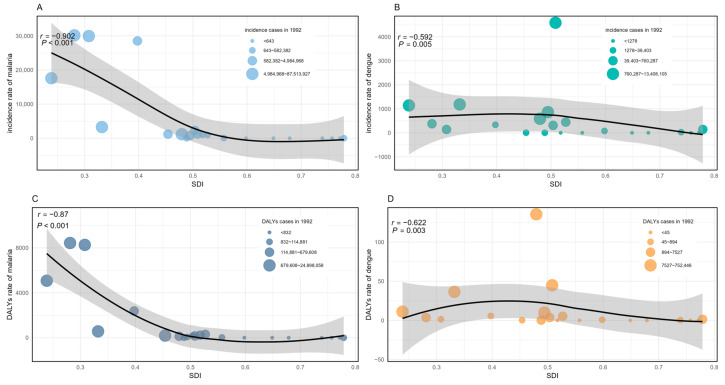
Relationship between the ASR of malaria or dengue and SDI by 21 GBD regions in 1992. Spearman correlation analysis between the SDI and ASIR (**A**) and ASR of DALYs (**C**) for malaria, and ASIR (**B**) and ASR of DALYs (**D**) for dengue across 21 regional levels in 1992 (The cases of incidence and DALYs from 21 regions in 1992 are represented by circles. The size of the circles increased with the cases of incidence and DALYs).

**Figure 3:**
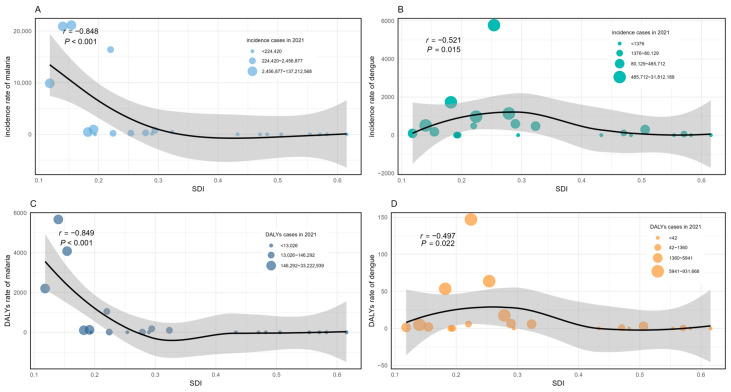
Relationship between the ASR of malaria or dengue and SDI by 21 GBD regions in 2021. Spearman correlation analysis between the SDI and ASIR (**A**) and ASR of DALYs (**C**) for malaria, and ASIR (**B**) and ASR of DALYs (**D**) for dengue across 21 regional levels in 2021 (The cases of incidence and DALYs from 21 regions in 2021 are represented by circles. The size of the circles increased with the cases of incidence and DALYs).

Across the 21 GBD regions, both diseases showed inverse correlations with SDI. For ASIR, the association was stronger for malaria (*r* = −0.848, *p* < 0.001) than for dengue (*r* = −0.521, *p* = 0.015), and the same pattern was observed for ASR of DALYs. Compared with 1992, correlations were weaker in 2021 (Figure 2 and Figure 3). Paired comparisons did not support a statistically significant change in the SDI-burden correlations between 1992 and 2021. Within-year comparisons also showed no significant differences between SDI-ASIR and SDI-DALY-rate correlations for either disease in 1992 or 2021 (all *p* > 0.05; Tables S9 and S10).

### 3.5. Age, Period, and Cohort Effects on Malaria and Dengue Incidence

In the APC analysis, malaria incidence declined overall, with a steeper reduction in males and the smallest decline in low SDI regions. Dengue incidence increased overall, although the increase was smaller in females. Notably, low SDI regions showed a declining dengue pattern, a pattern consistent with the EAPC results (Table 2). Sensitivity analyses using the lower and upper bounds of GBD 95% UI supported the overall direction of malaria decline. For dengue, the global and most non-low-SDI estimates remained positive, but the low-SDI estimates varied in magnitude and direction across lower- and upper-bound scenarios (Tables S11 and S12).

**Figure 4:**
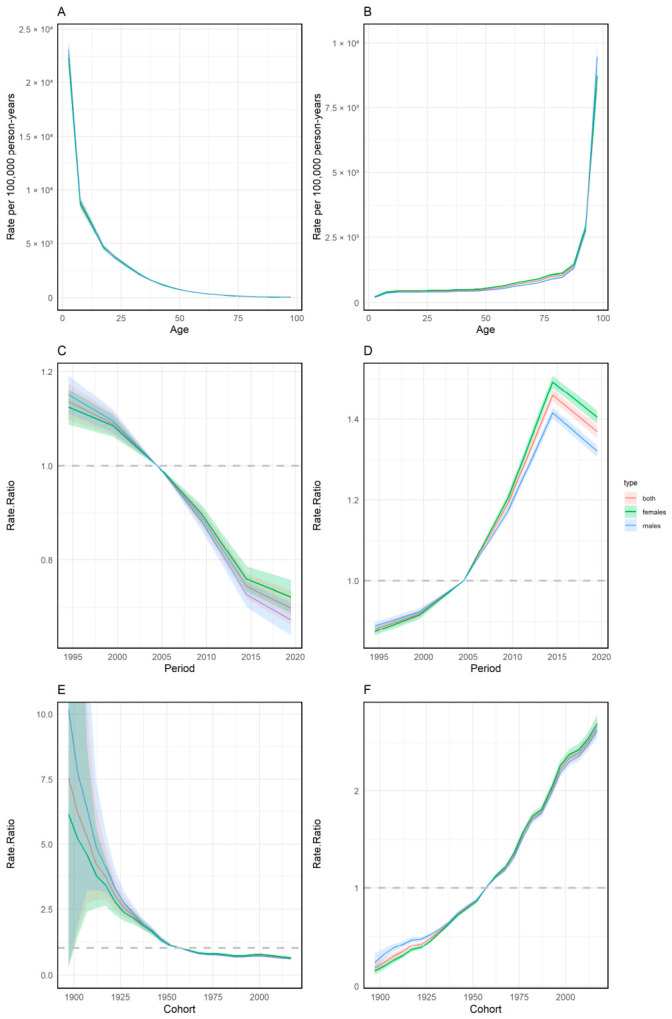
Age–period–cohort effect of incidence rate of malaria and dengue in global from 1992 to 2021. Age effect of malaria (**A**) and dengue (**B**); Period effect of malaria (**C**) and dengue (**D**); Cohort effect of malaria (**E**) and dengue (**F**). Expected values with 95%CI, based on global from 1992 to 2021, are shown as a solid line and shaded area. The dashed horizontal line indicates RR = 1 (reference).

Age effects differed clearly between diseases: malaria incidence risk was concentrated in children and declined with age, whereas dengue incidence risk increased with age and was highest among older adults. Sex differences were limited. Period effects showed a sustained decline for malaria and an overall increase for dengue, with a turning point around 2014. Cohort effects showed a wave-like decline in malaria risk after the 1957 birth cohorts and a stepwise increase in dengue risk (Figure 4). Most SDI strata followed the global APC pattern. Notable deviations were observed for dengue in low-SDI regions, where period effects increased after 2004 but cohort effects declined in later birth cohorts, and for malaria in high-middle and high-SDI regions, where the period effects increased or stabilized after 2014 (Figures S6–S10). Sensitivity analyses of the APC models demonstrated that the results across different SDI regions were generally consistent with the primary analyses. However, in the low SDI region, the age and cohort effects for dengue estimated using the lower-bound GBD inputs (2.5th percentile) were less stable, and the cohort effects based on the upper-bound inputs (97.5th percentile) also showed instability (Figure 4 and Figures S11–S22).

### 3.6. Projected Incidence of Malaria and Dengue to 2036

**Figure 5:**
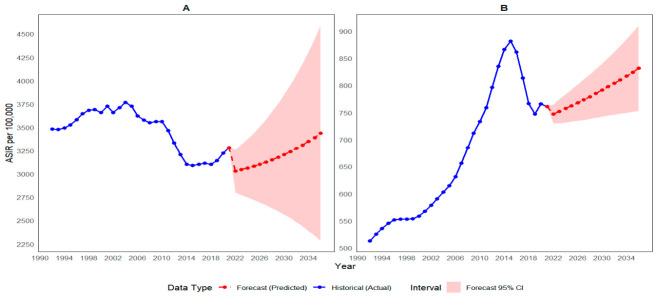
Prediction of age-standardized incidence rates of malaria and dengue in global from 1992 to 2036. The solid lines indicate the observed values (1992–2021) and the dotted lines are the predicted values (2022–2036). The ASIR of malaria (**A**) and dengue (**B**) globally.

Under the 1992–2011 training and 2012–2021 testing split, candidate BAPC models were compared using *DIC* and predictive error metrics (Tables S13 and S14). For the global both-sex projection, the selected malaria model used age = RW2, period = RW1, and cohort = RW2 (*DIC* = 7282.34; *RMSE* = 192.72; *MAE* = 176.19; *MAPE* = 5.60%), and the selected dengue model used age = RW1, period = RW1, and cohort = RW2 (*DIC* = 7060.21; *RMSE* = 71.15; *MAE* = 64.73; *MAPE* = 8.24%). BAPC projections indicated different near-term trajectories for the two diseases (Figure 5, Figures S11 and S12). Globally (Table S15), malaria ASIR was 3485.27 per 100,000 in 2021 and was projected to be 3207.74 (95%CI 2565.34 to 3850.14) in 2030 and 3437.72 (95%CI 2283.38 to 4592.05) in 2036. Dengue ASIR was 752.04 per 100,000 in 2021, was projected to increase to 791.31 (95%CI 742.53 to 840.09) in 2030, and was 831.63 (95%CI 752.64 to 910.61) in 2036. In males, malaria ASIR was 3193.24 in 2021 and was projected to be 3114.73 in 2030 and 3326.58 in 2036, whereas dengue ASIR was 704.83 in 2021 and was projected to reach 735.46 in 2030 and 775.06 in 2036. In females, malaria ASIR was 3379.82 in 2021 and was projected to be 3301.08 in 2030 and 3544.27 in 2036, whereas dengue ASIR was 819.74 in 2021 and was projected to reach 849.80 in 2030 and 892.13 in 2036.

## 4. Discussion

This study provides a comprehensive assessment of the global burden, long-term trends, and regional disparities of malaria and dengue across age groups, SDI regions, and national contexts, with projections of incidence risk through 2036. Our findings indicate divergent epidemiological trajectories: between 1992 and 2021, the global burden of malaria declined overall, whereas dengue burden increased substantially. In 2021, an estimated 249.11 million malaria cases were recorded worldwide, a 12.71% increase since 1992; however, the ASIR declined to 3485.27 per 100,000 (EAPC = −0.55%), and the ASR of DALYs decreased to 806.00 per 100,000 (EAPC = −1.64%), indicating a gradual reduction in population-level risk. In contrast, dengue cases reached 58.96 million in 2021, a 104.40% increase since 1992, with an ASIR of 752.04 per 100,000 (EAPC = 1.83%) and a rising ASR of DALYs (EAPC = 1.29%), reflecting a sustained upward trend in burden. Besides, marked regional heterogeneity was observed. Malaria burden remained highest in low SDI regions, particularly in Central and Western sub-Saharan Africa and Oceania, whereas dengue burden was most pronounced in middle SDI regions and Tropical Latin America. Together, these findings highlight the continued concentration of malaria in less developed settings alongside the expanding global footprint of dengue.

### 4.1. Divergent Epidemiological Trajectories of Viral and Parasitic Vector-Borne Diseases in the Context of Global Health Transition

Global disease burden trends reveal a divergence between dengue (a viral disease) and malaria (a parasitic infection). While improvements in sanitation and healthcare infrastructure have generally reduced the overall risk of parasitic diseases, such as malaria [12], the burden of viral infectious diseases, including dengue, has continued to rise, especially in the post COVID-19 era, driven by factors, such as viral evolution, immune evasion, climate change, urbanization, and increasing travel and trade [25,26,27,28,29]. The ASIR of dengue has climbed steadily since 1992, peaking around 2015 and then declining slowly. However, a slight rebound occurred in 2020–2021, and the overall trend remains upward. This trajectory aligns with observations by Jie Deng et al. [14,15], and the recent increase may be attributed to new data sources and updated estimation methods in GBD 2021, which also lead to variation in peak timing across studies. Moreover, although global malaria incidence has declined, the pace has slowed in recent years, and resistance to insecticides and antimalarial drugs remains a persistent challenge [30,31].

Dengue incidence and DALY burdens showed a broader age distribution than malaria. Incidence was elevated among adolescents, young adults, and the oldest age groups, whereas DALY rates were highest among young children and older adults. These groups, due to immune immaturity in young children or immune senescence plus comorbidities in older adults, experience more severe disease and longer recovery periods [16,32,33]. Higher observed incidence in older adults may reflect cumulative lifetime exposure in endemic settings, repeated heterotypic infections and immune history, demographic aging, health-care seeking and diagnostic ascertainment, and local changes in serotype circulation [34,35]. Malaria predominantly affects children and young adults under 30. Our study further found that children under 5 years old bear the highest burden of malaria significantly above other age groups. In the context of falling global fertility rates [36], these findings underscore the urgent need to strengthen maternal and child health interventions, early-life nutrition and care, and protective measures for infants and toddlers.

In our analysis across 21 GBD regions, there is a strong negative correlation between the SDI and both ASIR or ASR of DALYs for malaria and dengue. Descent in malaria and dengue burden correlates with higher SDI, indicating that dengue and malaria transmission is also strongly shaped by urbanization, water storage, mobility, climate suitability, and Aedes ecology [37]. Importantly, formal paired tests did not support a statistically significant change in the SDI-burden correlations between 1992 and 2021. This non-significant temporal change may reflect that regional SDI can mask substantial within-region inequalities and local ecological heterogeneity [37,38].

### 4.2. APC Analysis Reveals Opposing Temporal Trends and Persistent SDI

APC analyses further elucidate long-term trend differences between the two: malaria ASIR has been decreasing steadily (especially among males), while dengue shows a clear upward trend. Regions with low SDI show the smallest decline in malaria incidence, highlighting enduring challenges in malaria control in resource-poor settings. Interestingly, for dengue in low-SDI regions, the main analysis indicated a decline, yet sensitivity analyses showed unstable net-drift directions. This likely reflects greater data uncertainty where surveillance and laboratory confirmation are incomplete. Without laboratory testing, dengue is often misclassified as other febrile illnesses in low-resource settings [39]. Additionally, outbreak-driven reporting and variable diagnostic access may distort apparent trends, rather than signaling a genuine reversal in transmission [38,40]. Despite these uncertainties, the APC model reveals distinct patterns by age, period, and birth cohort. The high dengue risk in older adults contrasts sharply with the concentrated burden of malaria among young children, reflecting differences in transmission dynamics, accumulation of immune experience, and age-related exposure. Studies show that advanced age is an independent risk factor for severe/fatal dengue outcomes; comorbidities and immune aging exacerbate disease severity and prolong recovery in elders [33,41,42,43]. This may also contribute to the accumulation and transmission of cases among elderly individuals. Cohort effects indicate that birth cohorts after about 1957 show wave-like, progressive declines in malaria risk, whereas dengue risk rises in a stepped fashion, underlining intergenerational shifts in risk. Regional disparities are also pronounced. This anomalous observation may reflect complex interactions among climate variables (e.g., temperature, rainfall, humidity), ecological adaptations of mosquito vectors (e.g., changing distribution, density of *Aedes* spp.), and improvements in public health capacity (surveillance, diagnostics, vector control), which affect dengue transmission differently in areas of different development levels [44,45,46].

### 4.3. Conditional Projections Suggest Persistent Control Challenges and Potential Modification by Emerging Interventions

Our projections indicate divergent trajectories for malaria and dengue through 2036. For malaria, the projected ASIR declined initially and then increased slowly, although the 2036 estimate remained slightly below the 2021 level. This pattern may partly reflect the BAPC model structure: 2022 was the first out-of-sample year, and the random-walk priors smoothed the relatively high 2021 terminal estimate toward the latent age–period–cohort trajectory fitted from 1992 to 2021. The early decline is also compatible with the cumulative effects of existing control measures, including insecticide-treated nets, indoor residual spraying, rapid diagnostic tests, artemisinin-based combination therapies, chemoprevention, and surveillance [47,48]. However, the subsequent gradual increase suggests possible erosion of these gains. This later increase is in line with previous evidence that malaria progress has slowed since the mid-2010s, due to insecticide and antimalarial resistance, coverage gaps, health-system disruptions, population displacement, climate suitability, and vector adaptation [49,50]. The projected 2030 ASIR of 3207.74 per 100,000, compared with 3089.52 in 2015, indicates fragile progress and potential difficulty in achieving the WHO 2030 target under current age–period–cohort patterns [48]. In contrast, dengue incidence is projected to continue rising, approaching 791 per 100,000 (95%CI 742.53 to 840.09) by 2030, underscoring the limited effectiveness of old vector control strategies in curbing the global spread of Aedes-borne diseases [51]. The cyclical fluctuations and overall upward trajectory reflect the inherent complexity of dengue epidemiology, including serotype dynamics, cross-immunity, and the ecological adaptability of Aedes vectors, challenges that are magnified in rapidly urbanizing and climate-sensitive regions [45,52,53,54]. However, these projections are not deterministic. Emerging evidence suggests that novel interventions could substantially alter future trajectories. In a cluster-randomized trial, deployments of Wolbachia-infected mosquitoes significantly reduced the incidence of virologically confirmed dengue and dengue-related hospitalizations [55]. Likewise, the tetravalent dengue vaccine TAK-003 demonstrated sustained protective efficacy over 4.5 years of follow-up in a phase 3 trial, although effectiveness varied by serotype, age, baseline serostatus, and geographic setting [56]. If aggressively implemented, such strategies could shift future incidence toward the lower bound of our projections or even below the current forecasted trajectory. Thus, our projections may serve as a useful benchmark for identifying control gaps and evaluating the potential gains of future intervention efforts.

### 4.4. Study Limitations

This study has several limitations. First, as with all GBD-based analyses, our results depend on the availability and quality of underlying data in GBD 2021. Variability in reporting systems, completeness, and diagnostic accuracy across countries may introduce uncertainty [36]. In many nations with a high burden of mosquito-borne diseases, weak surveillance systems compromise the reliability of estimates. Second, discrepancies between GBD estimates and nationally reported dengue cases have been documented [57,58], underscoring the need for region-specific validation. Third, regional SDI may not fully capture within-region and within-country inequalities in housing, water storage, health-care access, and local vector control. Fourth, APC models are subject to the intrinsic identifiability problem, and estimates for dengue in the oldest age groups may be unstable due to sparse data and age-allocation artifacts. In low-SDI regions, data completeness and accuracy may be further limited by surveillance capacity, potentially introducing bias. Last, the BAPC forecasts represent continuation scenarios based on recent APC patterns. While these models implicitly capture net effects of climate and urbanization through period/cohort dimensions, they do not explicitly account for future changes in vaccines, therapeutics, or vector control, which could alter future trajectories.

## 5. Conclusions

The global burden of mosquito-borne diseases from 1992 to 2021 remains a pressing public health challenge, requiring sustained attention and tailored interventions. Our findings show that malaria, despite a declining trend, remains concentrated in low-SDI regions, particularly Central and Western sub-Saharan Africa and Oceania. In contrast, dengue displays a pronounced upward trajectory, with disproportionately high incidence and DALY burdens in middle SDI regions, Tropical Latin America, South Asia, and Southeast Asia. Strikingly, there is an expanding geographical reach of Aedes-borne viruses beyond traditional endemic areas. The heterogeneous distribution of malaria and dengue burden across SDI regions underscores the complex interplay of ecological, social, and economic determinants in shaping mosquito-borne disease transmission. These patterns support the need for integrated and context-specific control strategies, including expanded vaccine coverage where available, comprehensive vector management, and strengthened primary health-care systems, particularly in high-burden and resource-limited settings. Future progress in malaria prevention and dengue control will depend on translating existing scientific advances and emerging control strategies into equitable, context-specific implementation to curb further increases in disease burden.

## Figures and Tables

**Table 1:** Incidence and DALY rates of Malaria and Dengue in all ages in 2021, and EAPC from 1992 to 2021.

Characteristic	Incidence		DALYs	
	ASR (per 100,000, 95% UI), 2021	EAPC (%, 95%CI), 1992–2021	ASR (per 100,000, 95% UI), 2021	EAPC (%, 95%CI), 1992–2021
**Malaria**				
<5 years	14,631.95 (10,175.30, 21,120.91)	−0.57% (−0.87, −0.27) *	5778.06 (2350.49, 10,773.05)	−1.55% (−1.99, −1.11) *
5–9 years	5904.68 (3945.53, 9732.27)	−0.18% (−0.42, 0.06)	419.31 (170.49, 828.25)	−1.59% (−1.95, −1.23) *
10–14 years	4827.31 (3243.54, 7939.77)	−0.20% (−0.33, −0.08) *	247.25 (102.59, 507.91)	−1.28% (−1.62, −0.93) *
15–19 years	3655.56 (2692.49, 4807.60)	0.11% (−0.03, 0.26)	274.41 (111.14, 547.45)	−0.45% (−0.70, −0.19) *
20–24 years	2796.94 (2112.91, 3659.13)	−0.30% (−0.48, −0.11) *	269.47 (102.86, 551.57)	−0.42% (−0.82, −0.01) *
25–29 years	2067.60 (1605.13, 2678.71)	−0.66% (−0.88, −0.43) *	212.62 (77.91, 447.86)	−0.36% (−0.87, 0.16)
30–34 years	1455.10 (1132.86, 1863.68)	−0.98% (−1.16, −0.80) *	179.17 (64.06, 382.61)	−0.39% (−0.80, 0.03)
35–39 years	1134.60 (889.33, 1450.17)	−1.17% (−1.29, −1.05) *	186.20 (63.65, 415.05)	−0.43% (−0.74, −0.12) *
40–44 years	902.07 (711.62, 1155.05)	−1.56% (−1.72, −1.41) *	198.32 (66.89, 447.28)	−0.60% (−0.97, −0.23) *
45–49 years	651.08 (519.27, 837.42)	−2.20% (−2.40, −2.00) *	192.01 (64.32, 432.88)	−0.97% (−1.36, −0.58) *
50–54 years	468.90 (376.41, 604.28)	−2.77% (−2.97, −2.57) *	206.94 (67.19, 479.41)	−1.40% (−1.76, −1.04) *
55–59 years	352.02 (283.06, 451.71)	−3.14% (−3.38, −2.90) *	219.83 (70.89, 513.91)	−1.47% (−1.88, −1.06) *
60–64 years	286.29 (229.13, 369.34)	−3.47% (−3.85, −3.08) *	254.09 (82.72, 598.50)	−1.74% (−2.28, −1.20) *
65–69 years	208.78 (166.97, 272.66)	−3.53% (−4.08, −2.97) *	219.75 (70.11, 522.02)	−1.78% (−2.38, −1.18) *
70–74 years	163.81 (130.29, 220.63)	−3.49% (−4.04, −2.93) *	266.91 (87.48, 612.60)	−1.34% (−1.67, −1.01) *
75–79 years	139.14 (109.78, 191.18)	−3.58% (−4.06, −3.10) *	143.66 (45.11, 346.70)	−2.24% (−3.30, −1.16) *
80–84 years	103.48 (80.19, 146.94)	−3.79% (−4.33, −3.25) *	62.80 (21.62, 139.50)	−2.92% (−5.08, −0.72) *
85–89 years	78.86 (60.49, 115.45)	−4.25% (−4.85, −3.66) *	20.49 (7.71, 43.68)	−3.48% (−6.20, −0.69) *
90–94 years	60.56 (46.78,89.20)	−4.48% (−5.14, −3.81) *	5.65 (3.21,10.16)	−3.76% (−4.84, −2.68) *
95+ years	56.80 (44.01,77.03)	−4.05% (−4.56, −3.53) *	2.48 (1.78,3.26)	−4.61% (−4.83, −4.38) *
**Dengue**				
<5 years	527.54 (131.16, 978.12)	1.05% (0.82, 1.29) *	81.16 (42.65, 118.97)	0.26% (0.01, 0.52) *
5–9 years	888.19 (220.00, 1636.70)	1.23% (0.97, 1.49) *	36.22 (19.99, 53.85)	0.59% (0.23, 0.95) *
10–14 years	945.96 (229.24, 1751.97)	1.47% (1.12, 1.82) *	20.72 (8.50, 36.30)	1.89% (1.42, 2.37) *
15–19 years	923.17 (223.25, 1713.26)	1.72% (1.39, 2.06) *	22.16 (9.61, 37.34)	1.50% (1.20, 1.79) *
20–24 years	904.03 (224.69, 1661.77)	1.92% (1.72, 2.13) *	23.30 (10.05, 38.35)	1.72% (1.49, 1.94) *
25–29 years	844.88 (217.84, 1537.51)	2.04% (1.81, 2.28) *	23.00 (8.97, 39.08)	1.72% (1.48, 1.95) *
30–34 years	727.69 (191.20, 1316.48)	2.09% (1.72, 2.47) *	16.56 (6.57, 28.64)	2.76% (2.36, 3.16) *
35–39 years	733.70 (197.58, 1323.32)	2.25% (1.92, 2.58) *	17.62 (7.63, 28.88)	2.84% (2.51, 3.17) *
40–44 years	694.24 (193.63, 1245.46)	2.23% (2.00, 2.45) *	18.63 (8.08, 30.29)	3.01% (2.76, 3.26) *
45–49 years	613.41 (173.72, 1097.87)	2.05% (1.77, 2.34) *	17.81 (8.48, 28.85)	2.54% (2.27, 2.80) *
50–54 years	600.19 (175.24, 1068.86)	2.06% (1.69, 2.44) *	15.65 (6.97, 25.93)	2.34% (1.94, 2.74) *
55–59 years	591.24 (176.64, 1050.51)	2.20% (1.88, 2.53) *	19.00 (8.83, 30.26)	2.87% (2.56, 3.18) *
60–64 years	651.65 (193.46, 1154.36)	2.27% (2.03, 2.52) *	18.11 (8.73, 28.81)	2.50% (2.25, 2.75) *
65–69 years	606.87 (178.49, 1078.45)	2.45% (2.10, 2.81) *	16.38 (7.16, 26.22)	2.81% (2.43, 3.19) *
70–74 years	598.85 (173.53, 1070.44)	2.64% (2.27, 3.02) *	21.65 (11.66, 32.95)	2.73% (2.45, 3.01) *
75–79 years	661.43 (192.89, 1179.99)	2.62% (2.29, 2.96) *	20.49 (10.62, 31.62)	2.86% (2.61, 3.12) *
80–84 years	604.01 (178.69, 1078.39)	2.66% (2.30, 3.02) *	26.59 (15.69, 38.73)	3.39% (3.03, 3.76) *
85–89 years	672.86 (209.13, 1195.87)	2.51% (2.13, 2.89) *	30.60 (19.35, 42.59)	2.97% (2.51, 3.44) *
90–94 years	1120.30(370.02,1998.35)	2.44% (1.96, 2.93) *	35.21 (21.50,50.57)	2.74% (2.24, 3.26) *
95+ years	2768.78(1012.68,4885.70)	3.29% (2.48, 4.10) *	56.84 (32.02,87.41)	2.93% (2.17, 3.70) *

*: statistically significant at the 5% level. UI: uncertainty interval, CI: confidence interval, DALY: disability-adjusted life-year, EAPC: Estimated annual percentage change.

**Table 2:** Net drift of malaria and dengue incidence globally and across SDI regions from 1992 to 2021 (95%CI).

Group	Net Drift Value (95%CI)					
	Global	Low SDI	Low-Middle SDI	Middle SDI	High-Middle SDI	High SDI
**Malaria**						
both	−2.08 (−2.39, −1.77)	−1.35 (−1.88, −0.82)	−3.38 (−3.71, −3.05)	−3.99 (−4.46, −3.51)	−5.05 (−5.83, −4.26)	−13.39 (−13.79, −12.99)
females	−1.91 (−2.21, −1.62)	−1.38 (−1.90, −0.86)	−3.31 (−3.64, −2.99)	−3.81 (−4.27, −3.35)	−4.87 (−5.61, −4.14)	−12.81 (−13.26, −12.35)
males	−2.27 (−2.59, −1.95)	−1.32 (−1.86, −0.77)	−3.45 (−3.79, −3.11)	−4.15 (−4.65, −3.65)	−5.25 (−6.09, −4.40)	−14.09 (−14.45, −13.72)
**Dengue**						
both	2.18 (2.13, 2.24)	−0.66 (−0.73, −0.58)	1.52 (1.47, 1.56)	2.15 (2.09, 2.20)	3.66 (3.58, 3.75)	2.51 (2.34, 2.67)
females	2.33 (2.27, 2.38)	−0.86 (−0.94, −0.79)	1.53 (1.48, 1.58)	2.23 (2.17, 2.29)	3.76 (3.67, 3.85)	2.52 (2.35, 2.69)
males	1.97 (1.92, 2.03)	−0.42 (−0.50, −0.34)	1.48 (1.43, 1.52)	2.03 (1.98, 2.09)	3.53 (3.44, 3.61)	2.44 (2.25, 2.62)

Net drift reflects the overall annual percentage change in the ASIR from 1992 to 2021.

## Data Availability

The data sources and codes used in the GBD, Injuries, and Risk Factors Study 2021 are publicly available at https://gbd2021.healthdata.org/gbd-results (accessed on 18 November 2024).

## Supplementary Materials

The following supporting information can be downloaded at: https://www.mdpi.com/article/10.3390/tropicalmed11070201/s1, Table S1: Global and SDI trends of malaria and dengue incidence from 1992 to 2021; Table S2: Global and SDI trends of malaria and dengue DALYs from 1992 to 2021; Table S3: Cases and age-standardized rates of incidence and DALYs in 1992 and 2021, and their estimated annual percentage changes from 1992 to 2021 for malaria and dengue in all ages by 21 GBD regions; Table S4: Age-standardized rates of incidence in 1992 and 2021, and their estimated annual percentage changes from 1992 to 2021 for malaria in all ages, by country; Table S5: Age-standardized rates of incidence in 1992 and 2021, and their estimated annual percentage changes from 1992 to 2021 for dengue in all ages, by country; Table S6: Age-standardized rates of DALY in 2021, and their estimated annual percentage changes from 1992 to 2021 for malaria in all ages, by country; Table S7: Age-standardized rates of DALY in 2021, and their estimated annual percentage changes from 1992 to 2021 for dengue in all ages, by country; Table S8: Joinpoint regression analysis results for dengue and malaria burden; Table S9: Formal comparison of SDI-burden correlations between 1992 and 2021 across 21 GBD regions; Table S10: Formal comparison of SDI-ASIR and SDI-age-standardized DALY rate correlations within the same year; Table S11: APC sensitivity analysis using GBD lower-bound estimates: net drift of incidence, 1992–2021; Table S12: APC sensitivity analysis using GBD upper-bound estimates: net drift of incidence, 1992–2021; Table S13: Candidate BAPC model comparison under the 1992–2011 training and 2012–2021 testing split for malaria; Table S14: Candidate BAPC model comparison under the 1992–2011 training and 2012–2021 testing split for dengue; Table S15: Projected ASIR of malaria and dengue by sex, 2022–2036; Figure S1: Numbers of incident cases and DALY contributed by five GBD SDI, for malaria and dengue, in 1992–2021; Figure S2: EAPC of ASIR contributed by 204 country, for malaria, in 1992–2021; Figure S3: EAPC of age-standardized rates of DALY contributed by 204 country, for malaria, in 1992–2021; Figure S4: EAPC of ASIR contributed by 204 country, for dengue, in 1992–2021; Figure S5: EAPC of age-standardized rates of DALY contributed by 204 country, for dengue, in 1992–2021; Figure S6: APC effect of incidence rate of malaria and dengue in low SDI region from 1992 to 2021; Figure S7: APC effect of incidence rate of malaria and dengue in low-middle SDI region from 1992 to 2021; Figure S8: APC effect of incidence rate of malaria and dengue in middle SDI region from 1992 to 2021; Figure S9: APC effect of incidence rate of malaria and dengue in high-middle SDI region from 1992 to 2021; Figure S10: APC effect of incidence rate of malaria and dengue in high SDI region from 1992 to 2021; Figure S11: APC sensitivity analysis using GBD lower-bound estimates, global population; Figure S12: APC sensitivity analysis using GBD lower-bound estimates, low-SDI region; Figure S13: APC sensitivity analysis using GBD lower-bound estimates, low-middle-SDI region; Figure S14: APC sensitivity analysis using GBD lower-bound estimates, middle-SDI region; Figure S15: APC sensitivity analysis using GBD lower-bound estimates, high-middle-SDI region; Figure S16: APC sensitivity analysis using GBD lower-bound estimates, high-SDI region; Figure S17: APC sensitivity analysis using GBD upper-bound estimates, global population; Figure S18: APC sensitivity analysis using GBD upper-bound estimates, low-SDI region; Figure S19: APC sensitivity analysis using GBD upper-bound estimates, low-middle-SDI region; Figure S20: APC sensitivity analysis using GBD upper-bound estimates, middle-SDI region; Figure S21: APC sensitivity analysis using GBD upper-bound estimates, high-middle-SDI region; Figure S22: APC sensitivity analysis using GBD upper-bound estimates, high-SDI region; Figure S23: Prediction of age-standardized incidence rates of malaria and dengue by males in global from 1992 to 2036; Figure S24: Prediction of age-standardized incidence rates of malaria and dengue by females in global from 1992 to 2036.
