# Supplementary material for: Global, Regional, and National Burden of Malaria and Dengue from 1992 to 2021, with Projections to 2036: An Age–Period–Cohort Analysis
Source: Trop Med Infect Dis. 2026 Jul 17;11(7):201. doi: 10.3390/tropicalmed11070201 (PMC13418721; doi:10.3390/tropicalmed11070201)
Supplement: Supplementary file 1 [file tropicalmed-11-00201-s001.zip › tropicalmed-4368846-supplementary.pdf]

SUPPLEMENTARY MATERIAL

Content

Table S1. Global and SDI trends of malaria and dengue incidence from 1992 to 2021. 3
Table S2. Global and SDI trends of malaria and dengue DALYs from 1992 to 2021. 4
Table S3. Cases and age-standardized rates of incidence and DALYs in 1992 and 2021, and their estimated annual percentage changes from 1992 to 2021 for malaria and dengue in all ages by 21 GBD regions. 5
Table S4. Age-standardized rates of incidence in 1992 and 2021, and their estimated annual percentage changes from 1992 to 2021 for malaria in all ages, by country. 10
Table S5. Age-standardized rates of incidence in 1992 and 2021, and their estimated annual percentage changes from 1992 to 2021 for dengue in all ages, by country. 19
Table S6. Age-standardized rates of DALY in 2021, and their estimated annual percentage changes from 1992 to 2021 for malaria in all ages, by country. 27
Table S7. Age-standardized rates of DALY in 2021, and their estimated annual percentage changes from 1992 to 2021 for dengue in all ages, by country. 36
Table S8. Joinpoint regression analysis results for dengue and malaria burden. 44
Table S9. Formal comparison of SDI-burden correlations between 1992 and 2021 across 21 GBD regions. 45
Table S10. Formal comparison of SDI-ASIR and SDI-age-standardized DALY rate correlations within the same year. 45
Table S11 APC sensitivity analysis using GBD lower-bound estimates: net drift of incidence, 1992–2021. 46
Table S12 APC sensitivity analysis using GBD upper-bound estimates: net drift of incidence, 1992–2021. 46
Table S13. Candidate BAPC model comparison under the 1992–2011 training and 2012–2021 testing split for malaria. 47
Table S14. Candidate BAPC model comparison under the 1992–2011 training and 2012–2021 testing split for dengue. 48
Table S15. Projected ASIR of malaria and dengue by sex, 2022–2036. 49
Figure S1. Numbers of incident cases and DALY contributed by five GBD SDI, for malaria and dengue, in 1992-2021 50
Figure S2. EAPC of ASIR contributed by 204 country, for malaria, in 1992-2021 51
Figure S3. EAPC of age-standardized rates of DALY contributed by 204 country, for malaria, in 1992-2021 51
Figure S4. EAPC of ASIR contributed by 204 country, for dengue, in 1992-2021 52
Figure S5. EAPC of age-standardized rates of DALY contributed by 204 country, for dengue, in 1992-2021 52
Figure S6. APC effect of incidence rate of malaria and dengue in low SDI region from 1992 to 2021 53
Figure S7. APC effect of incidence rate of malaria and dengue in low-middle SDI region from 1992 to 2021 54
Figure S8. APC effect of incidence rate of malaria and dengue in middle SDI region from 1992 to 2021 55

Figure S9. APC effect of incidence rate of malaria and dengue in high-middle SDI region from 1992 to 2021 56
Figure S10. APC effect of incidence rate of malaria and dengue in high SDI region from 1992 to 2021........ 57
Figure S11. APC sensitivity analysis using GBD lower-bound estimates, global population........ 58
Figure S12. APC sensitivity analysis using GBD lower-bound estimates, low-SDI region........ 59
Figure S13. APC sensitivity analysis using GBD lower-bound estimates, low-middle-SDI region. 60
Figure S14. APC sensitivity analysis using GBD lower-bound estimates, middle-SDI region........ 61
Figure S15. APC sensitivity analysis using GBD lower-bound estimates, high-middle-SDI region. 62
Figure S16. APC sensitivity analysis using GBD lower-bound estimates, high-SDI region........ 63
Figure S17. APC sensitivity analysis using GBD upper-bound estimates, global population........ 64
Figure S18. APC sensitivity analysis using GBD upper-bound estimates, low-SDI region........ 65
Figure S19. APC sensitivity analysis using GBD upper-bound estimates, low-middle-SDI region. 66
Figure S20. APC sensitivity analysis using GBD upper-bound estimates, middle-SDI region........ 67
Figure S21. APC sensitivity analysis using GBD upper-bound estimates, high-middle-SDI region. 68
Figure S22. APC sensitivity analysis using GBD upper-bound estimates, high-SDI region........ 69
Figure S23. Prediction of age-standardized incidence rates of malaria and dengue by males in global from 1992 to 2036 70
Figure S24. Prediction of age-standardized incidence rates of malaria and dengue by females in global from 1992 to 2036 70

Table S1. Global and SDI trends of malaria and dengue incidence from 1992 to 2021.

Location	Disease	1992 Incidence Number (N×100,000, 95% UI)	1992 ASIR (per 100 000, 95% UI)	2021 Incidence Number (N×100,000, 95% UI)	2021 ASIR (per 100 000, 95% UI)	1992-2021 EAPC (%, 95% CI)
Global	Malaria	2210.29 (1862.93, 2665.12)	3678.17 (3108.77, 4424.7)	2491.17 (2003.59, 3150.17)	3485.27 (2804.46, 4435.69)	-0.55% (-0.75, -0.35)*
	Dengue	288.47 (62.07, 530.97)	510.67 (108.43, 941.74)	589.64 (154.73, 1068.85)	752.04 (196.33, 1363.35)	1.83% (1.55, 2.11)*
Low SDI	Malaria	1258.75 (1045.99, 1522.47)	17332.83 (14665.59, 20962.98)	1696.89 (1389.28, 2107.62)	11883.52 (9755.64, 14691.05)	-1.52% (-1.69, -1.36)*
	Dengue	27.34 (20.14, 38.93)	517.9 (385.13, 721.2)	39.33 (1.26, 95.80)	368.03 (11.69, 884.71)	-1.02% (-1.75, -0.29)*
Low-middle SDI	Malaria	722.09 (577.62, 912.75)	5105.15 (4104.83, 6398.96)	633.88 (489.62, 820.41)	3160.63 (2438.56, 4089.91)	-2.17% (-2.39, -1.96)*
	Dengue	100.30 (16.07, 194.52)	833.72 (129.09, 1624.69)	212.52 (22.95, 454.70)	1117.7 (123.23, 2373.55)	1.38% (1.16, 1.60)*
Middle SDI	Malaria	221.16 (180.80, 285.14)	1164.29 (958.79, 1488.22)	157.85 (105.02, 226.77)	750.12 (489.97, 1093.02)	-2.16% (-2.63, -1.68)*
	Dengue	146.06 (13.82, 302.01)	814.02 (78, 1684.53)	306.64 (105.47, 548.85)	1269.27 (437.36, 2268)	2.14% (1.79, 2.48)*
High-middle SDI	Malaria	5.92 (1.78, 15.97)	56.15 (16.02, 152.3)	0.81 (0.32, 2.08)	6.41 (2.5, 16.81)	-7.74% (-10.07, -5.35)*
	Dengue	11.16 (2.00, 24.87)	103.25 (18.45, 229.83)	24.94 (10.62, 43.08)	215.66 (91.61, 372.3)	3.43% (2.97, 3.90)*
High SDI	Malaria	0.20 (0.19, 0.22)	2.32 (2.22, 2.51)	0.01 (0.00, 0.02)	0.06 (0.01, 0.16)	-15.02% (-16.87, -13.12)*
	Dengue	3.48 (0.74, 7.76)	40.2 (8.61, 89.51)	6.01 (1.33, 13.14)	54.64 (12.22, 119.21)	1.66% (0.58, 2.76)*

95% UI = 95% uncertainty intervals; 95% CI = 95% Confidence Interval; ASIR = Age-Standardized Incidence Rate; EAPC = Estimated annual percentage change.

Table S2. Global and SDI trends of malaria and dengue DALYs from 1992 to 2021.

Location	Disease	1992		2021		1992-2021
		DALYs Number (N×10,000, 95% UI)	DALYs ASR (per 100 000, 95% UI)	DALYs Number (N×10,000, 95% UI)	DALYs ASR (per 100 000, 95% UI)	EAPC (%, 95% CI)
Global	Malaria	6000.03 (3070.51, 11080.19)	993.67（506.96, 1843.02）	5517.41 (2176.13, 10833.79)	806.00（318.93, 1570.18）	-1.64% (-2.25, -1.04)*
	Dengue	129.48 (93.13, 159.74)	22.06（16.09, 27.47）	207.65 (105.62, 313.07)	27.76（14.21, 41.65）	1.29% (0.95, 1.64)*
Low SDI	Malaria	3921.84 (2171.22, 6710.36)	4834.97 (2606.32, 8453.59)	3966.39 (1571.60, 7599.04)	2869.17 (1107.42, 5683.87)	-2.34% (-2.62, -2.05)*
	Dengue	7.09 (5.25, 9.24)	12.91 (9.69, 16.84)	11.88 (3.82, 22.24)	12.34 (4.44, 22.08)	0.36% (0.03, 0.69)*
Low-middle SDI	Malaria	1503.45 (663.95, 3214.15)	988.27 (434.67, 2134.12)	1196.23 (475.22, 2402.13)	630.94 (249.10, 1273.67)	-2.10% (-2.53, -1.66)*
	Dengue	47.96 (33.66, 62.04)	36.29 (25.26, 48.88)	79.39 (35.11, 129.04)	43.35 (19.69, 69.74)	1.07% (0.85, 1.29)*
Middle SDI	Malaria	564.90 (246.28, 1207.13)	298.97 (129.65, 637.71)	352.07 (136.39, 686.66)	177.62 (70.05, 334.71)	-2.32% (-2.86, -1.79)*
	Dengue	64.97 (42.96, 80.80)	34.23 (22.66, 43.16)	104.58 (57.53, 154.42)	48.78 (27.32, 71.02)	1.86% (1.53, 2.19)*
High-middle SDI	Malaria	6.19 (1.06, 37.13)	6.35 (1.00, 39.35)	0.71 (0.16, 1.86)	0.74 (0.14, 1.93)	-7.46% (-9.43, -5.45)*
	Dengue	8.97 (6.38, 12.12)	9.24 (6.47, 12.59)	11.01 (6.41, 15.62)	11.39 (6.67, 16.03)	0.97% (0.61, 1.33)*
High SDI	Malaria	0.76 (0.42, 1.28)	0.89 (0.48, 1.52)	0.05 (0.03, 0.07)	0.05 (0.03, 0.07)	-10.90% (-11.97, -9.82)*
	Dengue	0.37 (0.08, 0.92)	0.43 (0.10, 1.07)	0.65 (0.16, 1.53)	0.58 (0.14, 1.39)	1.80% (0.76, 2.86)*

95% UI = 95% uncertainty intervals; 95% CI = 95% Confidence Interval; EAPC = Estimated annual percentage change; SDI= sociodemographic index.

Table S3. Cases and age-standardized rates of incidence and DALYs in 1992 and 2021, and their estimated annual percentage changes from 1992 to 2021 for malaria and dengue in all ages by 21 GBD regions.

region	Incidence					DALYs				
	Number of cases, 1992	ASR per 100 000 population,1992	Number of cases, 2021	ASR per 100 000 population,2021	EAPC, (%, 95%CI) 1992-2021	Count,1992	ASR per 100 000 population,1992	Count,2021	ASR per 100 000 population,2021	EAPC, (%, 95%CI) 1992-2021
**Malaria**										
East Asia	186682.66 (154484.30, 226203.36)	14.60 (12.10, 17.67)	7696.93 (6007.71, 9719.29)	0.51 (0.40, 0.64)	-11.43% (-14.05, -8.74)	27542.34 (2634.17, 257752.68)	2.11 (0.21, 19.53)	224.61 (163.67, 308.63)	0.02 (0.01, 0.02)	-15.47% (-16.19, -14.74)
Southeast Asia	5786040.90 (4229576.36, 8479630.46)	1190.47 (889.69, 1707.36)	1435932.54 (1251087.90, 1636670.64)	199.78 (174.24, 227.94)	-6.25% (-6.91, -5.58)	679608.02 (179714.41, 2619799.36)	133.92 (34.34, 527.68)	110524.33 (34865.88, 363321.60)	16.06 (5.18, 52.09)	-5.87% (-7.88, -3.82)
Oceania	2391184.22 (1192216.80, 4668959.63)	28525.79 (15507.64, 52307.27)	2456877.32 (2144002.06, 2798559.37)	16416.24 (14480.86, 18511.18)	-2.74% (-3.37, -2.11)	165551.89 (37283.36, 552603.26)	2383.60 (503.74, 8143.99)	146292.01 (66771.79, 266017.95)	1047.48 (474.82, 1931.01)	-2.61% (-3.50, -1.71)
Central Asia	48223.25 (4477.73, 271554.30)	66.65 (6.43, 367.44)	-	-	-34.05% (-44.07, -22.24)	25435.11 (2685.50, 85645.57)	28.01 (3.15, 93.58)	-	-	-27.77% (-33.70, -21.31)
Central Europe	-	-	-	-	-	-	-	-	-	-
Eastern Europe	-	-	-	-	-	-	-	-	-	-
High-income Asia Pacific	1674.05 (704.79, 3389.36)	0.93 (0.39, 1.88)	439.01 (112.68, 1180.93)	0.23 (0.06, 0.61)	-7.51% (-9.96, -5.00)	2019.21 (1618.58, 2502.72)	1.14 (0.91, 1.42)	62.01 (51.10, 76.51)	0.03 (0.02, 0.04)	-12.61% (-15.44, -9.70)
Australasia	-	-	-	-	-	-	-	-	-	-

Western Europe	-		-	-	-	589.59 (321.22, 949.47)	0.13 (0.07, 0.22)	-	-	-13.07% (-16.30, -9.70)
Southern Latin America	643.01 (643.01, 643.01)	1.28 (1.28, 1.28)	-	-	-14.20% (-18.12, -10.09)	832.36 (705.02, 995.08)	1.64 (1.39, 1.96)	-	-	-19.60% (-27.69, -10.61)
High-income North America	-	-	-	-	-	-	-	-	-	-
Caribbean	364603.76 (280362.82, 486135.39)	961.94 (746.39, 1264.65)	224420.10 (128249.94, 441699.07)	477.10 (271.66, 943.05)	-3.65% (-4.79, -2.51)	117406.15 (50253.35, 246607.56)	301.24 (127.60, 636.54)	47765.20 (7055.56, 148307.18)	103.69 (15.42, 321.88)	-4.48% (-6.08, -2.85)
Andean Latin America	868916.15 (426923.34, 1976223.55)	2293.53 (1147.74, 5016.46)	84418.23 (28331.03, 153000.68)	125.91 (42.22, 228.26)	-9.69% (-10.89, -8.47)	80408.92 (22648.53, 205277.81)	183.41 (51.28, 469.58)	2153.97 (798.31, 7401.01)	3.24 (1.22, 11.06)	-14.79% (-16.96, -12.56)
Central Latin America	1229481.05 (1105692.79, 1368451.27)	745.33 (672.87, 824.84)	781377.70 (475267.29, 1229609.51)	301.91 (182.87, 476.52)	-2.82% (-4.87, -0.73)	114881.40 (51685.72, 214566.16)	59.25 (27.43, 109.62)	31985.88 (7411.69, 75722.58)	12.97 (3.02, 30.76)	-5.24% (-7.40, -3.02)
Tropical Latin America	2129621.42 (1770925.73, 2523742.62)	1238.20 (1037.28, 1462.51)	512283.10 (412736.16, 637002.09)	227.91 (182.21, 287.44)	-7.63% (-8.73, -6.51)	261149.82 (97550.62, 519485.91)	159.26 (58.79, 319.91)	13026.32 (3188.42, 33877.54)	6.01 (1.46, 15.63)	-12.88% (-13.84, -11.90)
North Africa and Middle East	4984968.16 (3564402.92, 7174244.64)	1207.85 (891.76, 1679.75)	6013613.75 (4093690.04, 8829737.27)	938.54 (638.93, 1381.99)	-2.34% (-3.76, -0.91)	762254.51 (311934.32, 1652790.71)	191.78 (74.16, 425.73)	786625.40 (237427.86, 1697098.01)	125.54 (37.81, 272.14)	-2.31% (-3.35, -1.27)
South Asia	38412765.74 (24168294.67, 66933343.52)	3278.54 (2152.37, 5299.96)	8408514.68 (5377410.99, 15276265.77)	446.38 (284.79, 813.55)	-6.13% (-6.82, -5.45)	8217894.89 (3040960.12, 23978917.57)	569.82 (208.18, 1683.69)	1820079.64 (126366.23, 6010614.07)	104.10 (6.91, 344.83)	-5.25% (-6.11, -4.38)

Central sub-Saharan Africa	25323341.62 (20719880.49, 30827986.57)	29943.34 (24876.89, 36559.94)	38283193.40 (30271382.72, 48712790.94)	21152.56 (16857.61, 26956.39)	-1.70% (-2.03, -1.38)	8355610.87 (4625866.72, 13289776.45)	8270.62 (4533.33, 13310.62)	7237039.49 (3416571.46, 12922264.35)	4076.68 (1861.99, 7643.64)	-3.14% (-3.58, -2.70)
Eastern sub-Saharan Africa	51204692.85 (42006489.00, 62868720.55)	17593.25 (14674.53, 21185.72)	53056139.78 (39641886.00, 66116041.16)	9882.91 (7375.20, 12284.83)	-2.59% (-2.97, -2.21)	16140723.05 (9549325.18, 26393871.21)	5077.89 (2912.68, 8489.11)	11609738.74 (4520723.84, 21788865.49)	2199.73 (830.69, 4301.55)	-4.53% (-5.10, -3.96)
Southern sub-Saharan Africa	582381.79 (427662.38, 896231.05)	1018.54 (760.80, 1522.36)	639154.02 (492420.49, 829700.38)	772.77 (596.75, 1000.85)	-0.62% (-2.13, 0.92)	150307.06 (51086.18, 331085.21)	227.72 (75.17, 513.51)	145604.46 (63098.15, 305447.87)	179.57 (77.69, 376.84)	0.30% (-1.10, 1.72)
Western sub-Saharan Africa	87513926.93 (71369613.77, 104531748.95)	30154.71 (24783.93, 36313.73)	137212567.80 (104765957.27, 169541524.11)	20913.32 (16198.50, 26342.27)	-1.55% (-1.72, -1.38)	24898058.15 (12912041.66, 42445121.10)	8440.70 (4345.22, 14587.99)	33222938.65 (13351431.17, 63411625.35)	5668.41 (2216.18, 11127.48)	-1.77% (-2.17, -1.36)
**Dengue**										
East Asia	36900.99 (5553.22, 94673.73)	3.06 (0.46, 7.86)	61440.30(15719.84,155194.76)	4.27(1.11,10.66)	1.18% (1.01, 1.35)	3856.38 (2419.24, 5326.60)	0.34 (0.21, 0.47)	1360.02(568.26,2540.32)	0.09(0.04,0.18)	-2.98% (-3.54, -2.43)
Southeast Asia	2864333.83 (735282.72, 6296711.48)	587.51 (150.84, 1281.39)	6728443.85(4787955.06,10431377.55)	971.89(691.33,1500.41)	2.34% (2.04, 2.65)	752445.82 (512617.67, 1117259.72)	135.51 (94.86, 196.20)	909114.20(589034.85, 1241630.50)	147.04(95.32,200.97)	0.76% (0.53, 0.99)
Oceania	22760.33 (4442.18, 55487.33)	334.93 (65.03, 808.08)	63970.37(28313.99,126070.14)	486.03(211.17,953.78)	1.69% (1.32, 2.06)	365.48 (118.56, 792.78)	5.69 (1.91, 12.01)	766.78(347.48,1540.46)	6.04(2.73,11.88)	0.42% (-0.16, 1.00)
Central Asia	-	-	-	-	-	-	-	-	-	-
Central Europe	-	-	-	-	-	-	-	-	-	-
Eastern Europe	-	-	-	-	-	-	-	-	-	-

High-income Asia Pacific	237967.76 (53409.36, 567464.28)	141.35 (31.74, 337.08)	485712.47(107597.24,1123586.02)	294.01(65.07,679.53)	3.25% (1.95, 4.57)	2341.27 (426.60, 6414.40)	1.40 (0.26, 3.82)	4713.22(875.96,12044.73)	2.90(0.52,7.39)	3.26% (1.95, 4.59)
Australasia	5935.85 (403.53, 16751.00)	28.70 (1.95, 80.99)	18447.84(5642.05,43236.80)	58.99(18.09,139.42)	3.93% (2.99, 4.87)	60.85 (3.64, 190.20)	0.29 (0.02, 0.92)	191.74(49.82,498.28)	0.61(0.16,1.60)	3.98% (3.04, 4.93)
Western Europe	-	-	-	-	-	45.22 (14.09, 94.85)	0.01 (0.00, 0.02)	1.49(0.51,2.74)	0.00 (0.00, 0.00)	-4.85% (-9.17, -0.32)
Southern Latin America	39403.23 (1319.78, 108695.93)	77.65 (2.60, 214.20)	80129.01(18026.93,188076.27)	118.83(26.67,278.96)	2.10% (1.59, 2.60)	412.06 (18.95, 1353.94)	0.81 (0.04, 2.66)	819.81(149.57,2211.01)	1.22(0.22,3.28)	2.01% (1.51, 2.52)
High-income North America	244.44 (34.61, 718.28)	0.09 (0.01, 0.25)	1376.40(40.25,7206.87)	0.36(0.01,1.86)	7.73% (6.29, 9.18)	6.44 (3.31, 11.98)	0.00 (0.00, 0.00)	42.09(15.85,121.12)	0.02(0.01,0.04)	8.30% (7.37, 9.23)
Caribbean	160674.70 (13270.86, 409265.31)	447.23 (37.30, 1145.83)	227073.50(29104.17,644225.75)	475.93(60.68,1325.33)	0.44% (-0.30, 1.18)	1962.56 (402.51, 4941.52)	5.38 (1.09, 13.61)	2751.25(546.46,7216.81)	5.83(1.17,15.48)	0.65% (-0.60, 1.91)
Andean Latin America	124103.84 (18347.83, 260099.63)	309.72 (45.94, 649.95)	391707.88(163083.19,669824.19)	593.22(247.03,1014.24)	2.17% (1.75, 2.60)	1500.95 (351.81, 3524.80)	3.64 (0.82, 8.58)	4327.20(1845.49,8129.16)	6.56(2.80,12.31)	2.33% (1.35, 3.32)
Central Latin America	1482847.01 (357315.93, 2699276.65)	862.85 (208.66, 1571.46)	2886641.39(1757443.51,4048875.24)	1140.37(694.53,1599.48)	2.77% (1.50, 4.05)	17856.03 (5961.11, 36543.25)	10.06 (3.27, 20.66)	43074.33(24432.76,67687.04)	17.53(9.92,27.60)	3.33% (2.27, 4.39)
Tropical Latin America	7292741.49 (714489.55, 16580924.18)	4586.17 (449.47, 10439.34)	13043195.24(3996125.94,26305621.50)	5774.82(1774.73,11624.76)	1.55% (0.97, 2.14)	71965.36 (7829.44, 179448.31)	44.84 (4.93, 111.88)	143869.56(50995.10,306624.28)	63.76(22.43,136.04)	2.20% (1.59, 2.81)
North Africa and Middle East	21693.16 (8818.63, 64837.29)	6.00 (2.46, 18.03)	53390.97(17761.11,188662.52)	8.50(2.86,30.02)	1.76% (0.69, 2.84)	894.02 (470.10, 1577.67)	0.25 (0.14, 0.42)	1035.07(445.39,2669.40)	0.18(0.08,0.45)	1.17% (-0.93, 3.30)

South Asia	13408104.63 (208182.02, 27802787.95)	1182.78 (18.60, 2452.17)	31812189.13(1872476.97,67070659.35)	1726.94(102.48,3635.94)	1.51% (1.44, 1.57)	409610.53 (202636.78, 663976.61)	36.46 (18.23, 58.98)	931668.08(324113.44, 1630476.70)	53.46(19.54,91.91)	1.91% (1.64, 2.18)
Central sub-Saharan Africa	81106.06 (6470.43, 378083.99)	140.16 (11.27, 648.07)	245096.55(11852.44,1403021.73)	178.08(8.62,1019.40)	1.08% (0.97, 1.19)	832.00 (63.58, 4598.71)	1.43 (0.11, 7.90)	2645.58(125.85,14646.47)	1.91(0.09,10.31)	1.13% (1.04, 1.23)
Eastern sub-Saharan Africa	2306898.88 (175673.50, 5735306.86)	1142.19 (87.44, 2843.11)	387627.24(6547.47,1462385.76)	94.37(1.55,364.39)	-8.94% (-11.16, -6.67)	23103.64 (1880.21, 58811.89)	11.14 (0.88, 28.33)	5940.78(1374.58,17522.87)	1.28(0.23,4.12)	-7.66% (-9.79, -5.47)
Southern sub-Saharan Africa	1277.68 (118.17, 5060.16)	2.18 (0.20, 8.62)	1086.66(57.33,7292.00)	1.32(0.07,8.83)	-2.62% (-3.48, -1.75)	14.48 (2.56, 65.24)	0.02 (0.00, 0.11)	12.28(1.13,79.37)	0.01(0.00,0.10)	-2.50% (-3.35, -1.65)
Western sub-Saharan Africa	760287.23 (1058.64, 2282200.69)	377.57 (0.52, 1129.44)	2476656.12(120934.32,8632874.73)	512.53(25.35,1792.58)	1.10% (0.99, 1.21)	7526.68 (64.23, 26157.86)	3.69 (0.02, 12.75)	24191.21(1156.70,83576.17)	4.93(0.24,17.05)	1.11% (1.00, 1.21)

Abbreviations: DALY=disability-adjusted life-years. EAPC=estimated annual percentage change. ASR=age-standardised rate. GBD=Global Burden of Diseases, Injuries, and Risk Factors Study. CI=confidence interval.

Table S4. Age-standardized rates of incidence in 1992 and 2021, and their estimated annual percentage changes from 1992 to 2021 for malaria in all ages, by country.

	1992		2021		1992 - 2021	Included
Location	Incidence Number	ASIR, per 100 000	Incidence Number	ASIR, per 100 000	EAPC 95%CI	years
**Afghanistan**	595984.84 (324668.27, 1008274.42)	5789.11 (3353.81, 9200.38)	213265.74 (171694.96, 263318.48)	772.70 (624.86, 950.36)	-5.26(-6.82, -3.67)	1992-2021
**Albania**	-	-	-	-	-	-
**Algeria**	1123.54 (201.55, 4016.94)	4.67 (0.84, 16.69)	-	-	-22.56(-26.17, -18.78)	1992-2013
**American Samoa**	-	-	-	-	-	-
**Andorra**	-	-	-	-	-	-
**Angola**	3588389.09 (2105888.53, 5254850.99)	22899.04 (14771.96, 32256.60)	7950609.80 (4771321.10, 11941955.46)	17668.89 (10760.77, 26623.06)	-2.08(-2.79, -1.36)	1992-2021
**Antigua and Barbuda**	-	-	-	-	-	-
**Argentina**	642.98 (642.98, 642.98)	1.92 (1.92, 1.92)	-	-	-14.20(-18.12, -10.09)	1992-2010
**Armenia**	-	-	-	-	-12.27(-39.39, 26.97)	1994, 1996-2005
**Australia**	-	-	-	-	-	-
**Austria**	-	-	-	-	-	-
**Azerbaijan**	169.16 (113.23, 245.55)	2.31 (1.55, 3.35)	-	-	-28.64(-38.81, -16.77)	1992-2012
**Bahamas**	-	-	-	-	-	-
**Bahrain**	-	-	-	-	-	-
**Bangladesh**	1214661.29 (378307.64, 3321423.71)	1179.42 (375.86, 3112.67)	57438.16 (50623.62, 64993.85)	34.45 (30.36, 38.98)	-10.53(-12.12, -8.90)	1992-2021
**Barbados**	-	-	-	-	-	-
**Belarus**	-	-	-	-	-	-
**Belgium**	-	-	-	-	-	-
**Belize**	26923.59 (87.07, 99605.93)	10905.08 (49.93, 37673.36)	-	-	-29.04(-31.80, -26.18)	1992-2018
**Benin**	2334040.11 (1711686.36, 2869830.88)	29627.04 (22087.97, 35801.53)	5186358.68 (3504781.74, 6717218.68)	27371.07 (18769.46, 35280.94)	-0.34(-0.63, -0.06)	1992-2021
**Bermuda**	-	-	-	-	-	-
**Bhutan**	362410.83 (113356.16, 862692.66)	44817.59 (16226.60, 102355.57)	64.47 (56.28, 74.60)	8.20 (7.16, 9.49)	-31.01(-33.27, -28.68)	1992-2021

**Bolivia**	133640.13 (61992.56, 261258.66)	2131.48 (1013.71, 4066.39)	7503.79 (5629.85, 9826.30)	63.69 (47.78, 83.40)	-9.97(-11.01, -8.91)	1992-2021
**Bosnia and Herzegovina**	-	-	-	-	-	-
**Botswana**	382.77 (329.86, 436.74)	30.74 (26.50, 35.08)	2900.46 (2466.95, 3378.21)	120.18 (102.21, 139.97)	-5.44(-8.51, -2.27)	1992-2021
**Brazil**	2123959.66 (1765368.40, 2517781.43)	1270.41 (1063.39, 1501.34)	512283.10 (412736.16, 637002.09)	235.88 (188.55, 297.92)	-7.58(-8.68, -6.47)	1992-2021
**Brunei Darussalam**	-	-	-	-	-	-
**Bulgaria**	-	-	-	-	-	-
**Burkina Faso**	6078565.86 (4905909.06, 7643353.18)	39980.90 (32219.61, 50024.86)	8594162.42 (5419217.49, 12383140.08)	26759.08 (17858.40, 37140.73)	-1.64(-1.98, -1.30)	1992-2021
**Burundi**	2744554.61 (1836500.20, 3368148.28)	31629.13 (22634.52, 39108.54)	3821730.66 (2267022.69, 5277253.87)	21282.80 (13890.45, 29788.54)	-2.81(-3.82, -1.79)	1992-2021
**Cabo Verde**	2.86 (2.07, 3.87)	0.88 (0.64, 1.19)	51.34 (42.52, 60.45)	8.88 (7.35, 10.45)	5.04(-3.87, 14.76)	1992-2019, 2021
**Cambodia**	504833.78 (424990.12, 598215.15)	4961.48 (4224.97, 5809.94)	16186.46 (13561.65, 19048.53)	95.14 (79.71, 111.96)	-8.00(-9.75, -6.22)	1992-2021
**Cameroon**	4824115.50 (3874513.65, 5800287.48)	29601.98 (23197.62, 36104.86)	7799106.89 (4913916.07, 11596155.65)	18889.84 (12234.39, 27349.75)	-2.12(-2.48, -1.76)	1992-2021
**Canada**	-	-	-	-	-	-
**Central African Republic**	1238765.69 (757550.23, 1627733.31)	30173.19 (19804.56, 39545.85)	1669883.10 (889584.50, 2659761.88)	23265.69 (13120.00, 36698.67)	-0.95(-1.17, -0.72)	1992-2021
**Chad**	1967410.99 (1245777.02, 2833714.40)	21084.03 (13882.83, 29786.77)	3621415.08 (2112961.26, 5867122.57)	14141.14 (8561.40, 21916.09)	-1.58(-1.82, -1.33)	1992-2021
**Chile**	-	-	-	-	-	-
**China**	186682.66 (154484.30, 226203.36)	15.12 (12.53, 18.30)	-	-	-28.49(-34.99, -21.33)	1992-2016
**Colombia**	553694.28 (465630.39, 655023.56)	1699.28 (1431.12, 2006.97)	116791.54 (60778.55, 199432.30)	225.81 (117.44, 385.79)	-6.92(-8.10, -5.71)	1992-2021
**Comoros**	60652.80 (480.25, 367732.53)	9946.41 (114.34, 56940.38)	12383.83 (10210.43, 14941.61)	1687.94 (1395.05, 2031.47)	-7.26(-10.66, -3.73)	1992-2021
**Congo**	898921.97 (643882.18, 1223738.80)	26096.01 (18986.07, 35024.20)	1170267.11 (754610.76, 1833684.48)	18817.37 (12065.08, 29733.93)	-1.78(-2.22, -1.35)	1992-2021
**Cook Islands**	-	-	-	-	-	-
**Costa Rica**	14770.48 (11912.06, 18120.60)	480.47 (387.87, 588.85)	356.82 (356.82, 356.82)	7.09 (7.09, 7.09)	-25.02(-30.58, -19.02)	1992-2014, 2016-2021
**Coted'Ivoire**	6927556.19 (5580555.12, 8701800.75)	35727.43 (29154.77, 45226.69)	7572558.82 (3778855.88, 11291312.74)	20865.90 (10810.75, 30262.36)	-2.50(-3.08, -1.92)	1992-2021
**Croatia**	-	-	-	-	-	-
**Cuba**	-	-	-	-	-	-

**Cyprus**	-	-	-	-	-	-
**Czechia**	-	-	-	-	-	-
**Democratic People's Republic of Korea**	-	-	7696.93 (6007.71, 9719.29)	26.89 (20.99, 33.96)	-12.01(-16.86, -6.88)	1998-2021
**Democratic Republic of the Congo**	19100481.01 (15383640.86, 24146421.23)	32250.28 (25739.39, 40889.97)	26756910.20 (20405699.16, 34661135.37)	22588.96 (17024.31, 28769.42)	-1.61(-1.94, -1.28)	1992-2021
**Denmark**	-	-	-	-	-	-
**Djibouti**	33315.24 (28461.72, 39118.75)	6490.59 (5654.93, 7471.25)	128243.28 (118143.18, 139168.99)	9947.73 (9195.70, 10761.56)	2.18(-5.52, 10.51)	1992-2021
**Dominica**	-	-	-	-	-	-
**Dominican Republic**	2270.19 (1732.49, 2893.85)	32.63 (24.90, 41.59)	2305.04 (582.77, 6352.09)	20.57 (5.20, 56.70)	-6.59(-8.81, -4.32)	1992-2021
**Ecuador**	110670.29 (93706.97, 127551.89)	1131.47 (959.30, 1302.82)	6539.50 (5267.11, 8038.43)	35.88 (28.90, 44.10)	-16.98(-20.77, -13.01)	1992-2021
**Egypt**	-	-	-	-	-	-
**El Salvador**	23111.64 (17492.50, 29856.46)	464.68 (352.40, 599.06)	-	-	-30.07(-32.07, -28.01)	1992-2016
**Equatorial Guinea**	148871.80 (89740.97, 202904.29)	22000.92 (13613.05, 29546.47)	321023.32 (150029.42, 503279.23)	17045.48 (8015.43, 26553.95)	-0.64(-0.90, -0.38)	1992-2021
**Eritrea**	55898.86 (15685.17, 152173.82)	1940.85 (569.67, 4963.03)	44287.34 (41676.75, 47079.13)	714.86 (673.04, 759.56)	-1.75(-4.32, 0.90)	1992-2021
**Estonia**	-	-	-	-	-	-
**Eswatini**	5604.14 (543.38, 21334.67)	725.61 (74.17, 2690.94)	1344.04 (1057.79, 1666.56)	121.83 (95.91, 151.03)	-9.92(-11.67, -8.13)	1992-2021
**Ethiopia**	1009023.12 (856577.36, 1182229.23)	1916.01 (1644.71, 2231.94)	3202830.19 (2630444.89, 3780922.59)	3047.59 (2507.64, 3576.32)	1.64(-1.17, 4.54)	1992-2021
**Fiji**	-	-	-	-	-	-
**Finland**	-	-	-	-	-	-
**France**	-	-	-	-	-	-
**Gabon**	347912.06 (217956.30, 498459.54)	25936.72 (17200.52, 36445.53)	414499.88 (198032.42, 722690.90)	19918.28 (9676.81, 34898.22)	-1.24(-2.22, -0.26)	1992-2021
**Gambia**	112435.66 (89866.88, 137320.08)	9457.95 (7863.68, 11142.99)	155233.82 (133984.88, 180202.42)	6278.26 (5506.80, 7167.69)	0.18(-1.63, 2.03)	1992-2021
**Georgia**	-	-	-	-	-20.38(-42.50, 10.26)	1996, 1998-2009
**Germany**	-	-	-	-	-	-
**Ghana**	7480019.61 (5609004.64, 8975868.55)	33990.11 (25340.84, 40663.65)	5701382.39 (3965275.84, 8149081.85)	13998.93 (9902.10, 19710.88)	-2.89(-3.49, -2.27)	1992-2021
**Greece**	-	-	-	-	-	-

**Greenland**	-	-	-	-	-	-
**Grenada**	-	-	-	-	-	-
**Guam**	-	-	-	-	-	-
**Guatemala**	264059.28 (191832.59, 356080.20)	3193.91 (2383.88, 4192.95)	-	-	-14.16(-15.83, -12.45)	1992-2020
**Guinea**	2982018.81 (2302172.10, 3859848.47)	32016.94 (24868.52, 41800.75)	4423676.17 (2339411.21, 6371023.40)	24017.74 (13371.39, 33220.04)	-0.89(-1.17, -0.60)	1992-2021
**Guinea-Bissau**	443624.55 (272567.77, 665783.30)	28905.99 (19049.49, 43296.04)	217427.67 (67954.93, 499155.18)	8353.06 (2630.02, 18310.44)	-6.27(-7.51, -5.01)	1992-2021
**Guyana**	162015.11 (128773.28, 199874.18)	19086.05 (15567.76, 22893.95)	91996.49 (75698.30, 111354.11)	11875.36 (9785.27, 14345.34)	-3.38(-5.07, -1.65)	1992-2021
**Haiti**	143767.81 (103320.30, 202645.92)	2280.64 (1664.54, 3158.02)	121342.15 (29828.98, 333983.84)	965.83 (239.37, 2625.56)	-3.89(-5.60, -2.15)	1992-2021
**Honduras**	199114.09 (153069.28, 254992.02)	4143.89 (3265.49, 5178.48)	2625.45 (80.99, 6827.15)	26.64 (0.82, 69.26)	-18.23(-19.58, -16.86)	1992-2021
**Hungary**	-	-	-	-	-	-
**Iceland**	-	-	-	-	-	-
**India**	31356461.69 (18588079.80, 56165429.95)	3447.14 (2129.04, 5795.87)	4323027.50 (1631909.94, 11240080.29)	298.19 (112.14, 778.45)	-7.40(-8.43, -6.36)	1992-2021
**Indonesia**	2429016.44 (986381.51, 5089898.44)	1214.27 (511.37, 2514.74)	1184668.80 (1012297.71, 1388599.56)	411.01 (350.78, 480.45)	-4.74(-5.39, -4.09)	1992-2021
**Iran (Islamic Republic of)**	237233.44 (186757.91, 295057.21)	401.89 (323.81, 492.15)	1001.09 (973.86, 1016.95)	1.15 (1.10, 1.17)	-27.08(-30.02, -24.02)	1992-2021
**Iraq**	24802.60 (17040.19, 34459.32)	147.48 (101.44, 204.66)	-	-	-43.69(-53.75, -31.43)	1992-2008
**Ireland**	-	-	-	-	-	-
**Israel**	-	-	-	-	-	-
**Italy**	-	-	-	-	-	-
**Jamaica**	-	-	-	-	-	-
**Japan**	-	-	-	-	-	-
**Jordan**	-	-	-	-	-	-
**Kazakhstan**	-	-	-	-	-	-
**Kenya**	6027261.44 (4664643.79, 7785685.30)	17018.88 (13364.20, 22102.52)	3154201.85 (2339594.31, 4078470.16)	5426.32 (3918.29, 6956.75)	-5.54(-6.46, -4.60)	1992-2021
**Kiribati**	-	-	-	-	-	-
**Kuwait**	-	-	-	-	-	-

**Kyrgyzstan**	-	-	-	-	-11.17(-36.96, 25.17)	1996, 1998, 2000-2010
**Lao People's Democratic Republic**	117532.95 (94784.52, 144510.12)	2899.46 (2361.24, 3529.53)	8657.20 (7233.06, 10223.32)	118.76 (99.23, 140.24)	-9.72(-11.60, -7.79)	1992-2021
**Latvia**	-	-	-	-	-	-
**Lebanon**	-	-	-	-	-	-
**Lesotho**	-	-	-	-	-	-
**Liberia**	888736.26 (506279.43, 1240502.38)	32030.85 (18167.23, 44024.98)	1932161.12 (1005407.64, 2808843.00)	27702.66 (14565.45, 38887.49)	-0.74(-1.12, -0.36)	1992-2021
**Libya**	-	-	-	-	-	-
**Lithuania**	-	-	-	-	-	-
**Luxembourg**	-	-	-	-	-	-
**Madagascar**	2857308.92 (2113640.36, 3633973.60)	16272.83 (12207.45, 20602.08)	2719663.53 (1489559.66, 4099411.08)	7802.36 (4338.01, 11387.17)	-3.71(-4.40, -3.02)	1992-2021
**Malawi**	4872410.32 (3858647.18, 6002750.51)	31646.47 (25577.78, 38960.58)	4287598.80 (2794318.36, 7136443.96)	17226.92 (11375.40, 28150.10)	-2.60(-2.94, -2.27)	1992-2021
**Malaysia**	80178.54 (74321.16, 86720.94)	450.98 (418.11, 487.68)	-	-	-22.59(-25.00, -20.09)	1992-2017
**Maldives**	-	-	-	-	-	-
**Mali**	4335641.02 (3473852.68, 5588159.39)	32625.53 (26387.00, 42003.19)	6762512.08 (4407723.74, 10064050.40)	19654.81 (13531.42, 28756.96)	-1.77(-2.10, -1.43)	1992-2021
**Malta**	-	-	-	-	-	-
**Marshall Islands**	-	-	-	-	-	-
**Mauritania**	72322.95 (51874.61, 99975.52)	3497.73 (2574.78, 4703.89)	108913.21 (88976.09, 132844.97)	2616.37 (2160.35, 3155.94)	-1.36(-3.67, 0.99)	1992-2021
**Mauritius**	-	-	-	-	-	-
**Mexico**	48681.60 (38413.74, 58470.18)	58.26 (46.02, 70.05)	692.61 (250.37, 1401.45)	0.52 (0.19, 1.05)	-14.47(-15.82, -13.10)	1992-2021
**Micronesia (Federated States of)**	-	-	-	-	-	-
**Monaco**	-	-	-	-	-	-
**Mongolia**	-	-	-	-	-	-
**Montenegro**	-	-	-	-	-	-

**Morocco**	1575.77 (1157.14, 2095.04)	6.44 (4.73, 8.56)	-	-	-36.37(-44.20, -27.44)	1992-2000, 2002-2004, 2010
**Mozambique**	6392517.17 (5298150.12, 7758530.30)	32911.44 (27066.89, 39772.49)	9958909.30 (7037360.38, 13088080.47)	23058.48 (16692.98, 29781.02)	-1.61(-1.80, -1.42)	1992-2021
**Myanmar**	496325.37 (413496.96, 591306.78)	1247.53 (1040.73, 1483.69)	202892.80 (171387.72, 239624.70)	356.12 (300.79, 420.63)	-3.57(-5.87, -1.22)	1992-2021
**Namibia**	70572.29 (6681.87, 293523.84)	4449.83 (497.53, 16121.41)	24238.71 (19897.38, 29246.82)	1024.55 (841.94, 1234.88)	-8.96(-13.57, -4.11)	1992-2021
**Nauru**	-	-	-	-	-	-
**Nepal**	131543.27 (108218.90, 159139.88)	720.66 (594.03, 869.35)	2586.17 (2224.58, 2985.48)	8.36 (7.19, 9.65)	-15.70(-17.94, -13.41)	1992-2021
**Netherlands**	-	-	-	-	-	-
**New Zealand**	-	-	-	-	-	-
**Nicaragua**	68025.14 (56109.26, 82063.09)	1825.96 (1520.31, 2180.02)	156577.84 (3436.93, 465218.47)	2340.84 (44.67, 6919.15)	-9.30(-14.61, -3.67)	1992-2021
**Niger**	3371079.40 (1787755.96, 4909873.97)	26067.93 (14915.20, 37175.15)	7996585.31 (4500690.55, 11480216.88)	21332.47 (13006.50, 30283.69)	-0.30(-0.72, 0.12)	1992-2021
**Nigeria**	41136466.60 (32325165.62, 50963191.41)	31091.78 (24919.73, 38855.77)	71275230.82 (50133632.44, 92032176.74)	22958.33 (16489.50, 29576.99)	-1.44(-1.69, -1.19)	1992-2021
**Niue**	-	-	-	-	-	-
**North Macedonia**	-	-	-	-	-	-
**Northern Mariana Islands**	-	-	-	-	-	-
**Norway**	-	-	-	-	-	-
**Oman**	14910.25 (14910.25, 14910.25)	775.31 (775.31, 775.31)	120.61 (1.02, 898.33)	2.43 (0.02, 18.12)	-15.12(-22.21, -7.39)	1992-2003, 2007-2008, 2010-2021
**Pakistan**	5347688.66 (1065721.21, 17077332.07)	4187.56 (981.39, 11923.54)	4025398.37 (3229412.39, 4940369.22)	1682.59 (1333.63, 2061.65)	-2.33(-3.13, -1.52)	1992-2021
**Palau**	-	-	-	-	-	-
**Palestine**	-	-	-	-	-	-
**Panama**	1992.28 (1609.04, 2460.53)	83.15 (67.17, 102.69)	5373.13 (967.41, 16166.12)	123.37 (22.20, 371.33)	-0.77(-3.85, 2.41)	1992-2021
**Papua New Guinea**	2018146.74 (979305.57, 4091501.91)	37971.00 (20254.82, 71482.24)	2141456.48 (1844478.24, 2468612.78)	19170.75 (16755.74, 21789.99)	-3.03(-3.65, -2.41)	1992-2021
**Paraguay**	5661.76 (4139.28, 7456.29)	145.41 (106.39, 191.36)	-	-	-17.01(-26.12, -6.77)	1992-2010

**Peru**	624605.73 (190951.84, 1709781.88)	2877.79 (905.41, 7629.45)	70374.95 (14603.75, 138835.46)	189.94 (39.41, 374.79)	-8.66(-10.09, -7.21)	1992-2021
**Philippines**	294860.44 (242076.22, 348958.73)	413.42 (336.82, 490.01)	13955.76 (11794.11, 16480.25)	12.23 (10.30, 14.41)	-11.34(-12.38, -10.29)	1992-2021
**Poland**	-	-	-	-	-	-
**Portugal**	-	-	-	-	-	-
**Puerto Rico**	-	-	-	-	-	-
**Qatar**	-	-	-	-	-	-
**Republic of Korea**	1674.05 (704.79, 3389.36)	3.58 (1.51, 7.25)	439.01 (112.68, 1180.93)	0.76 (0.19, 2.03)	-8.03(-10.46, -5.53)	1992-2021
**Republic of Moldova**	-	-	-	-	-	-
**Romania**	-	-	-	-	-	-
**Russian Federation**	-	-	-	-	-	-
**Rwanda**	1566849.50 (735055.84, 2632981.11)	14837.77 (7495.27, 24092.11)	560955.01 (394467.99, 760231.82)	3708.80 (2680.96, 5102.32)	-5.45(-6.62, -4.27)	1992-2021
**Saint Kitts and Nevis**	-	-	-	-	-	-
**Saint Lucia**	-	-	-	-	-	-
**Saint Vincent and the Grenadines**	-	-	-	-	-	-
**Samoa**	-	-	-	-	-	-
**San Marino**	-	-	-	-	-	-
**Sao Tome and Principe**	20394.33 (1476.92, 90723.47)	13105.63 (1326.50, 50476.39)	4654.01 (3987.82, 5408.44)	2208.03 (1900.09, 2554.71)	-8.97(-11.63, -6.23)	1992-2021
**Saudi Arabia**	18331.07 (18331.07, 18331.07)	116.79 (116.79, 116.79)	199.36 (9.67, 927.67)	0.48 (0.02, 2.25)	-22.11(-26.14, -17.86)	1992-2021
**Senegal**	783799.38 (306367.30, 1644527.67)	8763.97 (3961.30, 16199.13)	964429.83 (820205.60, 1111715.69)	5970.27 (5152.15, 6792.34)	-3.23(-4.27, -2.17)	1992-2021
**Serbia**	-	-	-	-	-	-
**Seychelles**	-	-	-	-	-	-
**Sierra Leone**	1919050.91 (996916.70, 2470817.13)	32636.32 (17749.23, 41999.93)	2908590.68 (1620543.40, 4125693.63)	25205.74 (14430.05, 36137.61)	-0.68(-0.90, -0.45)	1992-2021
**Singapore**	-	-	-	-	-	-
**Slovakia**	-	-	-	-	-	-
**Slovenia**	-	-	-	-	-	-
**Solomon Islands**	190136.34 (46762.41, 531505.19)	40527.69 (12607.23, 102866.89)	204460.98 (180713.44, 229523.78)	26543.37 (23807.76, 29509.52)	-4.12(-5.45, -2.77)	1992-2021

**Somalia**	1265105.24 (775108.23, 2188128.71)	11806.12 (7451.97, 19141.44)	1441048.22 (853916.31, 2034508.11)	5185.38 (3097.42, 7087.93)	-4.85(-6.02, -3.65)	1992-2021
**South Africa**	46347.07 (5047.35, 210246.49)	121.68 (13.45, 541.61)	25637.59 (19766.44, 32393.16)	44.32 (34.18, 56.04)	-5.67(-8.16, -3.11)	1992-2021
**South Sudan**	2258639.71 (1612690.10, 2934294.21)	26351.88 (19371.60, 33829.87)	2570192.31 (1500903.65, 3755125.65)	19786.01 (11968.37, 28729.63)	-1.21(-1.44, -0.97)	1992-2021
**Spain**	-	-	-	-	-	-
**Sri Lanka**	1127666.64 (1007720.58, 1256719.89)	6395.55 (5728.48, 7106.33)	-	-	-41.03(-45.70, -35.95)	1992-2012
**Sudan**	2513270.15 (1758794.99, 3352036.46)	9107.59 (6711.07, 12108.76)	3595545.13 (1732015.63, 6388852.10)	7016.30 (3418.36, 12471.18)	-2.87(-4.13, -1.61)	1992-2021
**Suriname**	17182.07 (1364.34, 88223.87)	4289.40 (360.49, 20800.19)	1181.80 (91.56, 2877.08)	197.96 (15.31, 482.59)	-17.80(-21.30, -14.15)	1992-2021
**Sweden**	-	-	-	-	-	-
**Switzerland**	-	-	-	-	-	-
**Syrian Arab Republic**	1759.66 (1254.53, 2470.62)	14.99 (10.69, 21.04)	-	-	-52.30(-59.98, -43.14)	1992-2004
**Taiwan (Province of China)**	-	-	-	-	-	-
**Tajikistan**	48021.65 (4244.69, 271342.77)	904.70 (85.78, 4904.78)	-	-	-33.78(-44.02, -21.68)	1992-2014
**Thailand**	510900.64 (461626.69, 568591.67)	866.21 (782.62, 964.11)	6265.78 (5398.92, 7240.93)	8.51 (7.33, 9.83)	-13.90(-15.53, -12.25)	1992-2021
**Timor-Leste**	65561.36 (4128.45, 313766.22)	7228.75 (560.16, 31728.15)	-	-	-22.83(-30.41, -14.43)	1992-2017, 2020
**Togo**	1833943.16 (1281367.19, 2335422.08)	31897.29 (23148.73, 39780.35)	1986516.46 (1098765.20, 2838515.68)	19235.85 (10868.64, 27835.10)	-1.82(-2.25, -1.40)	1992-2021
**Tokelau**	-	-	-	-	-	-
**Tonga**	-	-	-	-	-	-
**Trinidad and Tobago**	-	-	-	-	-	-
**Tunisia**	-	-	-	-	-	-
**Turkey**	12027.61 (3336.95, 31797.63)	21.05 (5.84, 55.64)	-	-	-26.06(-32.76, -18.69)	1992-2009
**Turkmenistan**	32.43 (20.95, 48.81)	0.93 (0.60, 1.39)	-	-	-10.99(-27.56, 9.37)	1992-1994, 1996-2006
**Tuvalu**	-	-	-	-	-	-
**Uganda**	9054359.34 (7187099.10, 11444203.48)	31489.47 (25600.61, 39475.78)	10269172.40 (7709279.91, 12875208.05)	17141.56 (12932.35, 20995.70)	-2.35(-2.68, -2.03)	1992-2021
**Ukraine**	-	-	-	-	-	-

**United Arab Emirates**	-	-	-	-	-	-
**United Kingdom**	-	-	-	-	-	-
**United Republic of Tanzania**	9850529.36 (7688977.30, 12491100.27)	25023.63 (19810.92, 31800.84)	7349549.86 (4695135.66, 10431617.57)	10156.33 (6568.53, 13820.26)	-4.33(-4.87, -3.79)	1992-2021
**United States Virgin Islands**	-	-	-	-	-	-
**United States of America**	-	-	-	-	-	-
**Uruguay**	-	-	-	-	-	-
**Uzbekistan**	-	-	-	-	-24.29(-41.69, -1.70)	1992-1991, 1999-2008, 2010
**Vanuatu**	31830.82 (28311.00, 35698.22)	18252.08 (16520.21, 20110.30)	652.98 (565.63, 751.12)	221.79 (192.18, 255.03)	-12.15(-15.02, -9.18)	1992-2021
**Venezuela (Bolivarian Republic of)**	56032.27 (47746.88, 65442.33)	301.67 (257.16, 352.09)	498960.32 (267138.62, 837972.73)	1834.52 (977.52, 3102.58)	9.43(6.94, 11.98)	1992-2021
**Viet Nam**	150771.05 (48741.25, 350448.92)	227.79 (73.83, 528.08)	1302.93 (1073.22, 1547.46)	1.25 (1.03, 1.48)	-13.96(-15.85, -12.03)	1992-2021
**Yemen**	1561190.78 (548929.46, 3372755.92)	9518.98 (4046.56, 17631.40)	2197872.91 (1696092.18, 2785475.77)	6391.84 (5059.80, 7910.35)	-2.11(-4.01, -0.17)	1992-2021
**Zambia**	3117567.59 (2371320.60, 4044871.59)	25500.46 (19806.17, 32741.21)	3489183.09 (2055667.08, 4939491.35)	13838.70 (8355.96, 19341.47)	-2.82(-3.46, -2.17)	1992-2021
**Zimbabwe**	459475.52 (364992.04, 567871.02)	4336.27 (3526.22, 5239.24)	585033.23 (435818.75, 771773.46)	3819.55 (2899.88, 4942.36)	-0.03(-1.57, 1.53)	1992-2021

Abbreviations: EAPC=estimated annual percentage change. ASR=age-standardized rate. GBD=Global Burden of Diseases, Injuries, and Risk Factors Study. CI=confidence interval.

Table S5. Age-standardized rates of incidence in 1992 and 2021, and their estimated annual percentage changes from 1992 to 2021 for dengue in all ages, by country.

	1992		2021		1992 - 2021	Included
**Location**	**Incidence Number**	**ASIR, per 100 000**	**Incidence Number**	**ASIR, per 100 000**	**EAPC 95%CI**	**years**
**Afghanistan**	139.95 (7.94, 680.70)	1.35 (0.08, 6.57)	628.07 (13.04, 4132.64)	2.00 (0.04, 13.15)	1.86(1.52,2.19)	1992-2021
**Albania**	-	-	-	-	-	-
**Algeria**	-	-	-	-	-	-
**American Samoa**	994.22 (246.95, 3136.92)	1933.89 (479.69, 6100.53)	1138.33 (249.56, 3838.12)	2281.21 (500.47, 7690.38)	0.64(0.06,1.24)	1992-2021
**Andorra**	-	-	-	-	-	-
**Angola**	4155.41 (246.68, 26644.26)	37.77 (2.24, 242.16)	69099.16 (2324.23, 478006.60)	209.75 (7.04, 1450.54)	7.40(6.82,7.98)	1992-2021
**Antigua and Barbuda**	125.81 (21.85, 260.45)	200.40 (34.79, 414.84)	45.17 (5.03, 122.86)	51.32 (5.71, 139.61)	-5.33(-6.67, -3.98)	1992-2021
**Argentina**	39401.31 (1319.72, 108690.65)	116.09 (3.88, 320.23)	80124.59 (18025.94, 188065.89)	175.50 (39.41, 411.99)	2.02(1.52,2.52)	1992-2021
**Armenia**	-	-	-	-	-	-
**Australia**	5935.85 (403.53, 16751.00)	34.52 (2.35, 97.41)	18447.84 (5642.05, 43236.80)	70.98 (21.76, 167.78)	3.91(2.98,4.85)	1992-2021
**Austria**	-	-	-	-	-	-
**Azerbaijan**	-	-	-	-	-	-
**Bahamas**	3188.23 (62.51, 9027.69)	1188.53 (23.27, 3365.24)	5306.68 (47.13, 18325.48)	1375.07 (12.21, 4748.53)	0.44(0.38,0.51)	1992-2021
**Bahrain**	-	-	-	-	-	-
**Bangladesh**	286866.46 (11703.31, 662831.81)	249.85 (10.13, 577.34)	700104.00 (115272.25, 1361863.05)	425.31 (70.01, 827.39)	1.99(1.80,2.19)	1992-2021
**Barbados**	4314.70 (468.48, 10008.18)	1693.51 (183.76, 3928.51)	5763.54 (649.30, 14314.93)	1933.88 (218.65, 4805.35)	1.57(-0.15,3.33)	1992-2021
**Belarus**	-	-	-	-	-	-
**Belgium**	-	-	-	-	-	-
**Belize**	691.88 (19.57, 1984.33)	346.12 (9.79, 992.68)	2066.23 (199.07, 6140.49)	478.91 (46.13, 1423.28)	2.48(0.71,4.27)	1992-2021
**Benin**	20331.31 (17.30, 135089.26)	390.04 (0.33, 2591.54)	68891.30 (1328.56, 504002.18)	507.13 (9.72, 3709.88)	1.52(1.14,1.90)	1992-2021
**Bermuda**	-	-	-	-	-	-
**Bhutan**	209.93 (3.15, 578.90)	32.20 (0.49, 88.79)	392.08 (10.40, 1105.15)	52.04 (1.38, 146.69)	5.19(0.60,9.99)	1992-2021
**Bolivia**	26897.48 (272.35, 74277.00)	399.35 (4.04, 1102.78)	82853.89 (15420.99, 183966.88)	705.09 (131.06, 1565.41)	2.64(2.16,3.11)	1992-2021

**Bosnia and Herzegovina**	-	-	-	-	-	-
**Botswana**	-	-	-	-	-	-
**Brazil**	7238527.86 (712701.62, 16454669.71)	4678.59 (460.90, 10647.10)	12863008.69 (3853875.59, 26106042.32)	5886.04 (1764.16, 11932.19)	1.56(0.97,2.15)	1992-2021
**Brunei Darussalam**	129.17 (11.68, 399.38)	47.50 (4.29, 146.88)	573.67 (150.80, 1426.02)	130.21 (34.37, 322.56)	4.70(3.43,5.99)	1992-2021
**Bulgaria**	-	-	-	-	-	-
**Burkina Faso**	5901.53 (10.78, 29416.82)	58.73 (0.11, 292.73)	32953.89 (1801.00, 192399.87)	144.20 (7.84, 841.83)	3.67(3.43,3.91)	1992-2021
**Burundi**	27090.97 (64.72, 93413.32)	465.68 (1.11, 1615.74)	2304.00 (38.52, 15878.00)	17.27 (0.29, 119.00)	-12.36(-15.63, -8.95)	1992-2021
**Cabo Verde**	21710.38 (1.51, 107999.59)	5731.39 (0.40, 28511.11)	43310.93 (96.09, 246752.91)	7751.51 (17.14, 44162.18)	0.95(0.85,1.05)	1992-2021
**Cambodia**	21472.80 (5379.04, 42978.50)	197.78 (49.71, 395.52)	42192.93 (8732.63, 92202.14)	249.20 (51.84, 544.45)	1.21(0.80,1.62)	1992-2021
**Cameroon**	23216.20 (32.32, 154553.33)	207.00 (0.29, 1378.01)	107185.93 (3438.57, 749780.16)	334.53 (10.75, 2340.03)	1.80(1.54,2.06)	1992-2021
**Canada**	-	-	-	-	-	-
**Central African Republic**	6136.64 (119.00, 35037.19)	213.76 (4.15, 1220.38)	13557.13 (64.44, 99012.04)	246.35 (1.18, 1799.12)	0.52(0.27,0.78)	1992-2021
**Chad**	2120.58 (5.71, 14235.62)	33.11 (0.09, 222.25)	8069.95 (206.64, 61253.47)	45.11 (1.16, 342.38)	0.38(0.08,0.69)	1992-2021
**Chile**	-	-	-	-	-	-
**China**	5000.50 (442.71, 14089.53)	0.42 (0.04, 1.18)	26748.49 (7382.49, 82676.68)	2.01 (0.55, 6.23)	5.77(4.75,6.81)	1992-2021
**Colombia**	357071.27 (84218.58, 732432.05)	1047.39 (247.32, 2148.22)	737205.57 (182961.68, 1464660.56)	1498.10 (372.27, 2977.05)	2.76(0.75,4.81)	1992-2021
**Comoros**	62823.00 (17700.85, 224500.48)	12931.32 (3644.60, 46120.61)	88297.89 (17.21, 589726.92)	11841.48 (2.30, 78807.32)	-1.27(-1.61, -0.93)	1992-2021
**Congo**	9793.46 (948.61, 50872.09)	381.55 (36.84, 1981.40)	16475.24 (663.11, 128353.51)	304.88 (12.29, 2375.15)	-0.13(-0.43,0.17)	1992-2021
**Cook Islands**	4.76 (0.90, 12.82)	24.73 (4.66, 66.52)	18.50 (0.48, 76.40)	104.33 (2.70, 430.79)	6.36(4.57,8.19)	1992-2021
**Costa Rica**	80803.59 (17827.13, 169492.51)	2484.41 (548.59, 5211.59)	127337.28 (18843.49, 315726.43)	2678.09 (394.95, 6641.53)	1.22(-0.56,3.03)	1992-2021
**Coted'Ivoire**	20824.22 (58.69, 148385.09)	157.59 (0.44, 1122.90)	58312.16 (1899.29, 450631.21)	208.89 (6.80, 1614.27)	1.26(0.91,1.62)	1992-2021
**Croatia**	-	-	-	-	-	-
**Cuba**	13126.09 (2479.63, 33148.07)	119.96 (22.66, 302.94)	19075.32 (3735.15, 51399.01)	169.39 (33.28, 456.79)	1.15(0.66,1.64)	1992-2021
**Cyprus**	-	-	-	-	-	-
**Czechia**	-	-	-	-	-	-
**Democratic People's Republic of Korea**	-	-	-	-	-	-

**Democratic Republic of the Congo**	56598.92 (3242.72, 351503.20)	140.35 (8.04, 871.54)	135941.83 (3188.36, 972141.66)	149.83 (3.52, 1071.33)	0.15(0.08,0.23)	1992-2021
**Denmark**	-	-	-	-	-	-
**Djibouti**	56459.45 (749.00, 145648.88)	11405.47 (151.36, 29459.02)	7676.69 (65.98, 54012.92)	614.93 (5.29, 4326.66)	-11.13(-13.24, -8.96)	1992-2021
**Dominica**	251.09 (16.67, 610.81)	347.12 (23.07, 844.28)	138.03 (12.23, 404.10)	205.80 (18.22, 602.47)	0.03(-2.76,2.89)	1992-2021
**Dominican Republic**	25826.43 (463.62, 73500.40)	344.85 (6.19, 981.41)	59174.22 (10394.24, 138442.80)	539.29 (94.77, 1261.66)	2.68(1.17,4.21)	1992-2021
**Ecuador**	33957.77 (6435.02, 78600.78)	321.35 (60.83, 743.76)	94195.64 (22766.17, 195368.48)	521.48 (126.07, 1082.87)	-0.08(-1.42,1.28)	1992-2021
**Egypt**	13067.67 (2254.08, 53443.58)	22.69 (3.91, 92.80)	27001.55 (1902.17, 138105.66)	25.64 (1.80, 131.15)	-0.02(-0.42,0.38)	1992-2021
**El Salvador**	55556.56 (5216.87, 140453.88)	1010.31 (94.95, 2553.83)	97129.76 (17376.74, 222202.47)	1488.30 (266.18, 3404.65)	2.22(1.10,3.36)	1992-2021
**Equatorial Guinea**	58.35 (9.13, 296.88)	12.73 (2.00, 64.70)	1809.13 (50.19, 13659.67)	117.92 (3.21, 890.43)	10.93(8.94,12.97)	1992-2021
**Eritrea**	7693.12 (78.59, 27479.79)	243.49 (2.48, 877.61)	689.53 (3.42, 5014.31)	10.46 (0.05, 76.04)	-11.56(-13.82, -9.24)	1992-2021
**Estonia**	-	-	-	-	-	-
**Eswatini**	-	-	-	-	-	-
**Ethiopia**	276438.94 (5474.73, 957522.87)	508.77 (9.88, 1759.39)	16461.96 (172.34, 79766.90)	14.90 (0.17, 71.88)	-12.47(-16.02, -8.76)	1992-2021
**Fiji**	7542.44 (509.58, 20813.46)	974.72 (66.03, 2689.53)	15396.98 (4508.88, 35131.81)	1682.41 (494.46, 3824.52)	2.00(1.49,2.51)	1992-2021
**Finland**	-	-	-	-	-	-
**France**	-	-	-	-	-	-
**Gabon**	4363.29 (257.11, 26830.12)	421.05 (24.75, 2588.90)	8214.05 (200.50, 50989.08)	450.39 (10.98, 2795.72)	-0.00(-0.37,0.37)	1992-2021
**Gambia**	2863.51 (8.97, 16291.69)	267.86 (0.84, 1523.97)	11163.67 (562.83, 79699.78)	461.20 (23.26, 3292.39)	1.48(1.06,1.90)	1992-2021
**Georgia**	-	-	-	-	-	-
**Germany**	-	-	-	-	-	-
**Ghana**	66246.10 (91.38, 426249.41)	420.46 (0.58, 2705.39)	245940.16 (6582.39, 1686497.06)	717.52 (19.11, 4919.80)	1.35(0.94,1.76)	1992-2021
**Greece**	-	-	-	-	-	-
**Greenland**	-	-	-	-	-	-
**Grenada**	68.21 (5.82, 161.41)	75.73 (6.47, 179.20)	344.21 (109.58, 898.92)	338.83 (107.94, 885.18)	5.31(4.92,5.70)	1992-2021
**Guam**	-	-	-	-	-	-
**Guatemala**	15990.00 (4740.63, 31846.01)	178.54 (52.93, 355.54)	36460.76 (7619.16, 78847.61)	229.39 (47.87, 495.98)	1.46(0.41,2.52)	1992-2021

**Guinea**	4955.12 (6.61, 46925.46)	76.47 (0.10, 724.22)	13709.68 (22.60, 88085.02)	101.09 (0.17, 649.45)	1.36(0.23,2.51)	1992-2021
**Guinea-Bissau**	2401.01 (2.02, 14212.35)	226.49 (0.19, 1340.65)	8481.04 (91.30, 60225.17)	408.49 (4.36, 2900.65)	1.77(1.56,1.99)	1992-2021
**Guyana**	1408.01 (7.76, 4102.33)	180.57 (0.99, 526.09)	1797.85 (157.75, 5150.10)	236.52 (20.77, 677.50)	2.39(0.83,3.97)	1992-2021
**Haiti**	21106.68 (69.81, 95797.09)	313.79 (1.04, 1424.22)	53436.46 (203.44, 287598.12)	417.09 (1.59, 2244.80)	-0.35(-1.31,0.62)	1992-2021
**Honduras**	45193.79 (6368.19, 94275.16)	895.69 (126.28, 1868.09)	89066.29 (8683.12, 248181.57)	877.03 (85.72, 2443.51)	0.65(-1.18,2.51)	1992-2021
**Hungary**	-	-	-	-	-	-
**Iceland**	-	-	-	-	-	-
**India**	11897113.82 (171637.22, 24759328.65)	1340.97 (19.70, 2790.78)	28205519.48 (1415602.02, 60205863.46)	1997.18 (100.91, 4261.45)	1.59(1.52,1.65)	1992-2021
**Indonesia**	1730262.91 (316199.46, 4556687.89)	890.60 (162.28, 2330.01)	2488470.07 (976958.08, 5092593.22)	906.32 (356.12, 1850.51)	0.29(0.13,0.44)	1992-2021
**Iran (Islamic Republic of)**	-	-	-	-	-	-
**Iraq**	-	-	-	-	-	-
**Ireland**	-	-	-	-	-	-
**Israel**	-	-	-	-	-	-
**Italy**	-	-	-	-	-	-
**Jamaica**	5717.44 (366.07, 13577.59)	233.30 (14.95, 554.01)	4351.77 (958.18, 10034.86)	153.60 (33.87, 354.18)	-0.14(-2.41,2.17)	1992-2021
**Japan**	-	-	-	-	-	-
**Jordan**	379.67 (35.19, 1253.56)	9.30 (0.86, 30.70)	1819.32 (59.84, 6495.13)	14.74 (0.48, 52.63)	5.42(1.96,9.00)	1992-2021
**Kazakhstan**	-	-	-	-	-	-
**Kenya**	635970.59 (49684.97, 2036061.99)	2576.66 (202.10, 8325.46)	169905.41 (2883.79, 1009757.83)	333.98 (5.54, 1966.90)	-7.08(-9.28, -4.83)	1992-2021
**Kiribati**	4410.98 (383.57, 14840.28)	5759.67 (504.44, 19371.74)	7399.50 (512.80, 29477.90)	6120.52 (424.68, 24379.43)	-0.23(-0.54,0.09)	1992-2021
**Kuwait**	1487.86 (143.52, 4407.83)	88.49 (8.54, 262.02)	2570.13 (171.80, 9195.02)	57.30 (3.83, 205.42)	1.25(-1.59,4.18)	1992-2021
**Kyrgyzstan**	-	-	-	-	-	-
**Lao People's Democratic Republic**	10658.97 (1803.29, 23100.45)	241.90 (41.05, 524.12)	23740.48 (5984.01, 48972.24)	323.91 (81.57, 669.51)	1.63(0.21,3.08)	1992-2021
**Latvia**	-	-	-	-	-	-
**Lebanon**	660.97 (110.73, 1885.93)	21.37 (3.58, 60.95)	1776.63 (83.79, 6187.52)	32.11 (1.51, 112.07)	4.25(1.21,7.38)	1992-2021
**Lesotho**	-	-	-	-	-	-

**Liberia**	8488.90 (7.70, 53223.12)	421.06 (0.38, 2639.90)	30117.66 (594.74, 194431.40)	547.61 (10.82, 3535.24)	1.05(0.70,1.40)	1992-2021
**Libya**	-	-	-	-	-	-
**Lithuania**	-	-	-	-	-	-
**Luxembourg**	-	-	-	-	-	-
**Madagascar**	49745.09 (578.39, 182561.55)	393.73 (4.58, 1446.57)	9763.55 (34.14, 73348.53)	33.87 (0.12, 254.41)	-8.62(-10.91, -6.29)	1992-2021
**Malawi**	79455.14 (483.73, 295419.88)	757.94 (4.61, 2824.21)	6114.74 (64.36, 41670.25)	30.91 (0.33, 210.64)	-12.13(-14.83, -9.35)	1992-2021
**Malaysia**	169303.37 (16947.36, 426296.53)	898.68 (89.92, 2262.79)	816195.19 (308260.24, 2067784.11)	2586.69 (976.79, 6552.92)	5.67(4.67,6.67)	1992-2021
**Maldives**	871.60 (0.00, 3500.66)	365.92 (0.00, 1471.73)	25895.36 (0.00, 93392.40)	5095.16 (0.00, 18143.76)	10.30(8.56,12.08)	1992-2021
**Mali**	4315.79 (13.13, 29102.08)	48.03 (0.15, 323.86)	14288.66 (416.77, 126646.24)	58.82 (1.72, 521.37)	1.08(0.95,1.20)	1992-2021
**Malta**	-	-	-	-	-	-
**Marshall Islands**	3872.30 (731.26, 16365.60)	8021.62 (1521.21, 33819.39)	5794.76 (594.17, 27794.37)	10324.23 (1060.09, 49396.09)	1.05(0.59,1.52)	1992-2021
**Mauritania**	582.24 (1.32, 3834.43)	26.85 (0.06, 176.80)	1874.30 (104.97, 14407.07)	42.14 (2.36, 323.87)	1.91(1.66,2.16)	1992-2021
**Mauritius**	4028.00 (519.80, 12730.31)	360.90 (46.50, 1137.27)	12712.83 (347.09, 51032.76)	1009.32 (27.58, 4044.79)	3.51(3.08,3.94)	1992-2021
**Mexico**	677830.08 (101808.51, 1303068.04)	758.69 (114.16, 1458.37)	1403089.58 (1004122.45, 1818973.34)	1088.49 (778.93, 1410.87)	3.59(2.02,5.18)	1992-2021
**Micronesia (Federated States of)**	500.82 (83.71, 1344.92)	465.14 (77.69, 1249.01)	443.95 (64.28, 1330.75)	432.72 (62.45, 1296.72)	-0.29(-0.62,0.05)	1992-2021
**Monaco**	-	-	-	-	-	-
**Mongolia**	-	-	-	-	-	-
**Montenegro**	-	-	-	-	-	-
**Morocco**	-	-	-	-	-	-
**Mozambique**	102840.88 (3043.61, 325539.49)	756.83 (22.36, 2388.77)	35948.61 (63.33, 264454.64)	114.33 (0.20, 841.09)	-5.86(-7.64, -4.05)	1992-2021
**Myanmar**	31044.23 (5971.96, 71284.66)	74.32 (14.32, 170.63)	44433.31 (9641.46, 98008.84)	79.26 (17.15, 174.81)	2.13(0.99,3.28)	1992-2021
**Namibia**	-	-	-	-	-	-
**Nauru**	2.46 (0.00, 9.51)	23.79 (0.00, 91.94)	117.67 (0.00, 495.43)	1067.73 (0.00, 4466.61)	18.94(13.14,25.03)	1992-2021
**Nepal**	105509.10 (282.15, 282157.85)	518.61 (1.39, 1386.88)	276879.89 (2739.29, 813633.54)	887.98 (8.79, 2609.38)	2.21(2.00,2.42)	1992-2021
**Netherlands**	-	-	-	-	-	-
**New Zealand**	-	-	-	-	-	-

**Nicaragua**	20133.59 (2176.79, 51008.80)	480.21 (51.99, 1216.37)	75311.63 (28329.55, 179689.35)	1129.92 (425.63, 2678.84)	3.69(3.13,4.26)	1992-2021
**Niger**	3277.65 (6.75, 19726.44)	38.30 (0.08, 230.49)	9172.65 (292.49, 78420.24)	36.29 (1.16, 310.24)	0.03(-0.29,0.35)	1992-2021
**Nigeria**	529483.25 (478.88, 1705938.85)	555.63 (0.50, 1816.10)	1719289.79 (46741.20, 6118737.90)	747.78 (20.95, 2627.93)	1.10(0.93,1.27)	1992-2021
**Niue**	1.66 (0.29, 5.29)	72.77 (12.62, 232.11)	1.93 (0.46, 5.45)	115.73 (27.82, 327.21)	1.91(1.42,2.40)	1992-2021
**North Macedonia**	-	-	-	-	-	-
**Northern Mariana Islands**	239.32 (59.25, 595.15)	512.21 (127.08, 1277.41)	360.45 (37.44, 1375.54)	756.57 (78.56, 2894.85)	0.42(-0.92,1.77)	1992-2021
**Norway**	-	-	-	-	-	-
**Oman**	234.73 (8.60, 1822.26)	12.75 (0.47, 99.06)	1005.94 (4.93, 5648.12)	22.61 (0.11, 126.97)	2.13(1.49,2.77)	1992-2021
**Pakistan**	1118405.31 (8168.92, 2953762.58)	963.78 (6.97, 2547.36)	2629293.69 (53357.04, 7216114.34)	1125.50 (22.41, 3092.40)	0.55(0.50,0.61)	1992-2021
**Palau**	555.20 (14.82, 2480.34)	3479.62 (92.95, 15544.81)	572.29 (0.92, 3069.39)	3257.77 (5.23, 17472.55)	-1.49(-2.50, -0.48)	1992-2021
**Palestine**	371.60 (48.83, 1069.81)	16.72 (2.20, 48.02)	1022.57 (61.47, 3764.55)	19.76 (1.19, 73.23)	3.00(0.21,5.87)	1992-2021
**Panama**	14176.57 (2281.47, 31807.33)	563.66 (90.71, 1264.64)	50264.03 (18240.10, 142788.40)	1164.04 (422.32, 3306.10)	2.07(1.29,2.87)	1992-2021
**Papua New Guinea**	665.61 (209.43, 1702.95)	15.31 (4.82, 39.17)	3410.93 (268.61, 11497.61)	32.80 (2.58, 110.56)	1.91(0.36,3.48)	1992-2021
**Paraguay**	54213.63 (1055.55, 159431.43)	1254.68 (24.38, 3689.74)	180186.55 (48740.40, 380401.65)	2506.64 (678.47, 5302.03)	2.39(1.93,2.86)	1992-2021
**Peru**	63248.59 (8700.83, 145291.74)	278.46 (38.32, 639.65)	214658.36 (67021.75, 534029.51)	592.85 (185.10, 1475.31)	3.55(3.12,3.98)	1992-2021
**Philippines**	117681.16 (17522.03, 264083.45)	176.46 (26.18, 394.60)	1262723.28 (299748.02, 4486387.08)	1111.20 (265.17, 3936.42)	7.20(6.68,7.72)	1992-2021
**Poland**	-	-	-	-	-	-
**Portugal**	-	-	-	-	-	-
**Puerto Rico**	70022.09 (4333.39, 232368.80)	1902.79 (117.76, 6314.40)	57086.63 (106.97, 230221.79)	1667.65 (3.17, 6725.48)	-0.12(-0.72,0.48)	1992-2021
**Qatar**	-	-	-	-	-	-
**Republic of Korea**	-	-	-	-	-	-
**Republic of Moldova**	-	-	-	-	-	-
**Romania**	-	-	-	-	-	-
**Russian Federation**	-	-	-	-	-	-
**Rwanda**	38149.47 (98.41, 141891.83)	504.27 (1.30, 1887.07)	1557.73 (69.09, 10399.38)	11.69 (0.52, 78.01)	-14.23(-17.19, -11.16)	1992-2021
**Saint Kitts and Nevis**	35.62 (0.34, 211.74)	84.36 (0.81, 501.47)	39.41 (1.48, 300.75)	68.79 (2.59, 524.95)	-0.20(-0.51,0.11)	1992-2021

**Saint Lucia**	191.19 (28.25, 446.87)	134.66 (19.84, 314.66)	435.41 (98.73, 1059.93)	248.35 (56.22, 605.96)	3.15(2.47,3.84)	1992-2021
**Saint Vincent and the Grenadines**	621.23 (79.88, 1444.62)	554.51 (71.42, 1289.21)	385.10 (15.94, 1236.29)	339.61 (14.05, 1090.24)	-1.27(-2.35, -0.17)	1992-2021
**Samoa**	338.83 (45.32, 874.04)	196.33 (26.30, 506.34)	1184.31 (364.51, 3456.93)	553.46 (170.35, 1609.97)	3.22(1.83,4.63)	1992-2021
**San Marino**	-	-	-	-	-	-
**Sao Tome and Principe**	977.37 (1.74, 5391.76)	761.59 (1.35, 4201.37)	1734.12 (64.37, 12623.68)	793.84 (29.45, 5778.64)	0.13(-0.09,0.36)	1992-2021
**Saudi Arabia**	241.55 (81.41, 475.99)	1.41 (0.48, 2.80)	1761.00 (500.43, 5195.49)	4.82 (1.37, 14.25)	5.03(4.23,5.83)	1992-2021
**Senegal**	3190.03 (8.22, 14960.85)	39.29 (0.10, 184.28)	11027.39 (1846.37, 40606.54)	68.95 (11.54, 253.79)	2.26(1.96,2.55)	1992-2021
**Serbia**	-	-	-	-	-	-
**Seychelles**	5642.82 (44.43, 27987.15)	7504.55 (59.16, 37220.62)	12843.68 (2396.14, 54245.72)	12417.92 (2317.99, 52444.37)	1.77(1.48,2.07)	1992-2021
**Sierra Leone**	22293.07 (25.91, 164631.24)	536.24 (0.62, 3960.09)	47375.83 (988.24, 333472.39)	530.75 (11.07, 3735.94)	0.02(-0.25,0.29)	1992-2021
**Singapore**	237838.59 (53384.38, 567158.31)	7475.43 (1678.33, 17825.01)	485138.80 (107231.32, 1122957.87)	8714.74 (1927.54, 20177.45)	1.15(-0.10,2.40)	1992-2021
**Slovakia**	-	-	-	-	-	-
**Slovenia**	-	-	-	-	-	-
**Solomon Islands**	1457.46 (4.48, 6120.33)	400.55 (1.24, 1682.00)	7402.71 (1452.83, 22563.37)	1083.53 (212.62, 3293.28)	3.68(2.93,4.44)	1992-2021
**Somalia**	65539.49 (548.70, 228922.34)	842.83 (7.05, 2944.87)	8759.56 (26.12, 67737.59)	40.42 (0.12, 312.55)	-11.15(-13.38, -8.86)	1992-2021
**South Africa**	-	-	-	-	-	-
**South Sudan**	44438.53 (5.07, 162239.78)	719.42 (0.08, 2636.54)	92.52 (3.61, 526.42)	0.95 (0.04, 5.39)	-25.61(-29.15, -21.88)	1992-2021
**Spain**	-	-	-	-	-	-
**Sri Lanka**	81413.68 (4607.33, 229559.30)	464.81 (26.31, 1310.60)	466305.90 (122171.51, 1557128.99)	2106.45 (552.08, 7025.59)	6.53(5.85,7.20)	1992-2021
**Sudan**	2490.39 (103.78, 11188.69)	11.45 (0.48, 51.43)	7162.15 (202.51, 46822.50)	16.32 (0.46, 106.71)	1.45(0.58,2.34)	1992-2021
**Suriname**	1386.00 (231.07, 3313.47)	351.89 (58.77, 841.17)	1714.14 (35.16, 5179.96)	296.70 (6.09, 896.60)	-3.11(-5.36, -0.82)	1992-2021
**Sweden**	-	-	-	-	-	-
**Switzerland**	-	-	-	-	-	-
**Syrian Arab Republic**	485.83 (69.54, 1381.27)	3.53 (0.50, 10.03)	519.37 (43.14, 1717.90)	3.70 (0.31, 12.24)	2.79(-0.55,6.25)	1992-2021
**Taiwan (Province of China)**	31900.49 (4719.47, 86652.35)	153.83 (22.79, 417.85)	34691.82 (2321.39, 125737.96)	147.88 (9.94, 536.03)	-0.06(-0.36,0.25)	1992-2021
**Tajikistan**	-	-	-	-	-	-

**Thailand**	192342.64 (53769.65, 369539.48)	329.38 (91.91, 632.70)	416690.11 (114970.54, 918117.67)	630.40 (174.78, 1381.13)	4.40(3.57,5.24)	1992-2021
**Timor-Leste**	679.02 (86.48, 1902.78)	83.81 (10.67, 234.86)	3124.76 (825.83, 8006.75)	221.18 (58.52, 566.70)	3.59(2.75,4.43)	1992-2021
**Togo**	17085.48 (20.17, 124010.56)	437.88 (0.52, 3178.21)	43728.11 (31.80, 318823.92)	521.12 (0.38, 3799.49)	0.63(0.35,0.90)	1992-2021
**Tokelau**	0.11 (0.00, 0.41)	6.93 (0.00, 26.13)	0.55 (0.00, 2.33)	39.22 (0.00, 167.87)	8.04(5.48,10.66)	1992-2021
**Tonga**	369.16 (56.61, 1293.79)	367.81 (56.32, 1288.69)	15323.74 (145.57, 54321.62)	14363.29 (136.46, 50916.52)	18.11(13.32,23.11)	1992-2021
**Trinidad and Tobago**	6856.89 (729.07, 18031.46)	556.15 (59.11, 1462.43)	8068.35 (363.25, 24299.81)	583.17 (26.24, 1756.41)	-0.03(-0.84,0.78)	1992-2021
**Tunisia**	-	-	-	-	-	-
**Turkey**	-	-	-	-	-	-
**Turkmenistan**	-	-	-	-	-	-
**Tuvalu**	4.22 (0.00, 16.88)	45.18 (0.00, 181.35)	55.77 (0.00, 241.79)	451.94 (0.00, 1964.59)	9.99(7.48,12.55)	1992-2021
**Uganda**	274907.89 (1400.60, 982929.30)	1489.84 (7.58, 5348.95)	21737.88 (265.94, 178217.82)	49.58 (0.60, 406.47)	-12.62(-15.26, -9.89)	1992-2021
**Ukraine**	-	-	-	-	-	-
**United Arab Emirates**	-	-	-	-	-	-
**United Kingdom**	-	-	-	-	-	-
**United Republic of Tanzania**	500331.96 (342.35, 1727761.06)	1816.95 (1.24, 6262.53)	9740.37 (224.65, 82107.68)	16.52 (0.38, 139.28)	-16.43(-20.17, -12.53)	1992-2021
**United States Virgin Islands**	252.81 (15.56, 1119.79)	239.38 (14.70, 1060.23)	160.56 (2.61, 917.48)	187.33 (3.06, 1070.51)	-0.73(-1.03, -0.43)	1992-2021
**United States of America**	244.43 (34.61, 718.26)	0.09 (0.01, 0.28)	1376.38 (40.25, 7206.76)	0.40 (0.01, 2.07)	7.73(6.30,9.18)	1992-2021
**Uruguay**	-	-	-	-	-	-
**Uzbekistan**	-	-	-	-	-	-
**Vanuatu**	362.83 (73.66, 841.83)	226.86 (46.04, 526.36)	2475.93 (703.16, 7230.73)	791.73 (224.85, 2317.10)	5.02(4.13,5.93)	1992-2021
**Venezuela (Bolivarian Republic of)**	216091.57 (50311.65, 436568.75)	1089.44 (253.88, 2200.67)	270776.48 (34700.09, 665509.49)	1022.45 (131.00, 2513.07)	0.25(-1.19,1.71)	1992-2021
**Viet Nam**	494777.39 (142482.58, 972566.47)	691.04 (198.03, 1357.65)	1103731.18 (409581.22, 2670612.12)	1118.13 (415.08, 2700.97)	1.53(1.26,1.80)	1992-2021
**Yemen**	2120.94 (103.65, 10954.74)	14.40 (0.70, 74.35)	8074.44 (807.72, 42838.31)	23.76 (2.37, 126.03)	1.75(1.56,1.93)	1992-2021
**Zambia**	83270.84 (533.79, 289700.55)	993.88 (6.37, 3448.41)	8239.33 (123.86, 53052.85)	41.76 (0.63, 268.92)	-11.71(-14.43, -8.91)	1992-2021
**Zimbabwe**	1277.68 (118.17, 5060.16)	11.61 (1.07, 45.96)	1086.66 (57.33, 7292.00)	6.93 (0.36, 46.47)	-2.64(-3.41, -1.86)	1992-2021

Abbreviations: EAPC=estimated annual percentage change. ASR=age-standardized rate. GBD=Global Burden of Diseases, Injuries, and Risk Factors Study. CI=confidence interval.

Table S6. Age-standardized rates of DALY in 2021, and their estimated annual percentage changes from 1992 to 2021 for malaria in all ages, by country.

	1992		2021		1992 - 2021	Included
Location	DALY Number	DALY ASR, per 100 000	DALY Number	DALY ASR, per 100 000	EAPC 95%CI	years
Afghanistan	19987.35 (4923.12,77540.72)	174.99 (42.16,682.07)	9488.24 (5132.00,13638.53)	28.87 (15.34,42.11)	-4.60(-7.69, -1.42)	1992-2021
Albania	-	-	-	-	-	-
Algeria	580.04 (406.26,785.20)	2.14 (1.51,2.86)	-	-	-4.81(-7.32, -2.23)	1992-2014
American Samoa	-	-	-	-	-	-
Andorra	-	-	-	-	-	-
Angola	577276.39 (207755.44,1144511.59)	3403.76 (1212.17,6858.35)	1354462.00 (592483.48,2699269.64)	3417.13 (1399.90,7141.20)	-2.48(-3.57, -1.38)	1992-2021
Antigua and Barbuda	-	-	-	-	-	-
Argentina	832.32 (704.99,995.03)	2.47 (2.09,2.95)	-	-	-19.73(-27.84, -10.70)	1992-2014
Armenia	-	-	-	-	-29.99(-39.85, -18.50)	1994, 1996-2009
Australia	-	-	-	-	-	-
Austria	-	-	-	-	-	-
Azerbaijan	304.91 (242.03,384.28)	3.86 (3.08,4.82)	-	-	-18.46(-22.71, -13.98)	1992-2014
Bahamas	-	-	-	-	-	-
Bahrain	-	-	-	-	-	-
Bangladesh	825668.12 (118242.29,2726803.30)	662.58 (90.88,2240.76)	35155.38 (9650.83,68721.94)	20.83 (5.74,40.71)	-9.29(-11.07, -7.48)	1992-2021
Barbados	-	-	-	-	-	-
Belarus	-	-	-	-	-	-
Belgium	-	-	-	-	-	-
Belize	88.39 (8.25,333.47)	37.94 (3.62,139.07)	-	-	-19.59(-21.53, -17.61)	1992-2018
Benin	469187.41 (257832.80,730773.61)	5721.98 (3086.00,9004.30)	1141758.47 (568075.77,1965939.00)	6891.46 (3261.99,12411.94)	0.48(-0.18,1.16)	1992-2021
Bermuda	-	-	-	-	-	-
Bhutan	40004.76 (7171.66,106888.06)	4668.77 (837.66,12854.23)	21.01 (15.14,29.15)	2.96 (2.13,4.16)	-27.08(-29.16, -24.94)	1992-2021
Bolivia	4327.88 (1411.60,13726.12)	54.32 (17.89,169.37)	206.79 (131.11,302.83)	1.73 (1.11,2.52)	-10.22(-11.26, -9.16)	1992-2021

**Bosnia and Herzegovina**	-	-	-	-	-	-
**Botswana**	253.40 (160.73,391.49)	17.15 (10.85,27.55)	684.78 (422.68,1158.85)	27.85 (17.12,47.75)	-6.49(-8.87, -4.06)	1992-2021
**Brazil**	261021.16 (97421.43,519356.77)	164.32 (60.60,330.06)	13026.32 (3188.42,33877.54)	6.22 (1.52,16.19)	-12.86(-13.83, -11.89)	1992-2021
**Brunei Darussalam**	-	-	-	-	-	-
**Bulgaria**	-	-	-	-	-	-
**Burkina Faso**	1725419.16 (933952.38,2884106.12)	11781.96 (6387.03,19882.51)	2570349.15 (1233546.05,4419193.83)	8938.18 (4195.32,15779.33)	-1.28(-1.88, -0.68)	1992-2021
**Burundi**	1249158.07 (670904.83,1896854.99)	12748.15 (6748.17,19783.16)	1096291.16 (514109.29,1972713.76)	5978.63 (2755.33,11435.02)	-4.76(-5.75, -3.76)	1992-2021
**Cabo Verde**	93.39 (67.65,125.41)	25.34 (18.37,33.78)	140.22 (95.12,188.90)	24.02 (16.16,32.04)	-0.19(-0.95,0.57)	1992-2021
**Cambodia**	69287.29 (32745.47,137627.74)	502.99 (234.00,1008.22)	995.63 (713.84,1374.14)	5.74 (4.12,7.90)	-9.28(-12.17, -6.30)	1992-2021
**Cameroon**	1141114.81 (624012.10,1834316.24)	7330.35 (3959.19,11947.94)	1934040.63 (798432.28,3723213.95)	5668.07 (2298.15,11312.99)	-2.05(-2.77, -1.33)	1992-2021
**Canada**	-	-	-	-	-	-
**Central African Republic**	203419.31 (92754.47,363612.11)	4516.16 (2035.10,8158.71)	369574.11 (149612.75,814948.20)	5663.66 (2226.20,12789.37)	0.43(-0.25,1.11)	1992-2021
**Chad**	457719.79 (235159.72,854873.92)	4513.31 (2354.30,8375.87)	668876.68 (234075.88,1349937.51)	2831.17 (978.18,5744.53)	-1.94(-2.26, -1.62)	1992-2021
**Chile**	-	-	-	-	-	-
**China**	27542.34 (2634.17,257752.68)	2.18 (0.22,20.20)	-	-	-19.53(-21.23, -17.79)	1992-2018
**Colombia**	93740.90 (36146.41,183494.06)	241.77 (93.33,472.42)	4884.12 (1416.72,8805.68)	11.21 (3.22,20.41)	-9.02(-10.68, -7.34)	1992-2021
**Comoros**	6876.76 (219.57,46451.02)	1384.99 (42.40,9503.70)	1777.50 (886.05,2878.16)	234.27 (116.63,380.55)	-4.16(-8.16,0.01)	1992-2021
**Congo**	165930.38 (80118.64,282943.48)	4901.91 (2295.72,8387.34)	161819.36 (65796.07,292470.88)	2991.66 (1206.30,5628.13)	-2.82(-3.35, -2.29)	1992-2021
**Cook Islands**	-	-	-	-	-	-
**Costa Rica**	146.78 (76.40,319.27)	4.32 (2.34,9.15)	12.55 (10.52,15.36)	0.26 (0.21,0.32)	-9.97(-11.07, -8.85)	1992-2014, 2016-2021
**Coted'Ivoire**	1711466.28 (897913.01,2735754.92)	9514.47 (4790.92,15736.91)	1892222.81 (817228.09,3360686.75)	6120.71 (2558.20,11294.53)	-2.18(-3.02, -1.33)	1992-2021
**Croatia**	-	-	-	-	-	-
**Cuba**	-	-	-	-	-	-
**Cyprus**	-	-	-	-	-	-
**Czechia**	-	-	-	-	-	2016

**Democratic People's Republic of Korea**	-	-	224.61 (163.67,308.63)	0.88 (0.62,1.22)	-6.22(-9.10, -3.24)	1996-2021
**Democratic Republic of the Congo**	7289039.09 (4165812.97,11259114.69)	10127.78 (5806.61,15773.44)	5236327.99 (2581069.83,8931618.56)	4348.68 (2096.08,7681.37)	-3.30(-3.71, -2.89)	1992-2021
**Denmark**	-	-	-	-	-	-
**Djibouti**	12179.72 (4572.42,23719.19)	2176.78 (817.03,4336.27)	10749.28 (6185.91,16161.70)	845.62 (488.76,1264.66)	-1.05(-5.88,4.03)	1992-2021
**Dominica**	-	-	-	-	-	-
**Dominican Republic**	796.34 (284.79,1735.60)	10.37 (3.70,22.64)	505.29 (83.89,2063.19)	4.38 (0.73,17.86)	-7.48(-9.35, -5.58)	1992-2021
**Ecuador**	21906.52 (8331.54,44345.57)	184.08 (69.84,372.78)	128.88 (81.41,184.99)	0.71 (0.45,1.02)	-22.60(-26.31, -18.69)	1992-2021
**Egypt**	-	-	-	-	-	-
**El Salvador**	253.06 (187.08,340.72)	4.35 (3.30,5.74)	-	-	-14.11(-17.63, -10.45)	1992-2018
**Equatorial Guinea**	55510.45 (26039.81,105451.04)	7383.17 (3370.95,14294.26)	67106.34 (30225.61,125415.30)	4404.33 (1924.79,9111.23)	-2.90(-3.24, -2.57)	1992-2021
**Eritrea**	12114.95 (2140.20,35282.87)	360.97 (64.08,1050.86)	10641.29 (6932.48,16719.50)	157.27 (102.30,247.58)	-1.96(-4.03,0.14)	1992-2021
**Estonia**	-	-	-	-	-	-
**Eswatini**	1445.49 (272.17,3780.98)	156.45 (28.53,413.66)	317.36 (136.69,784.96)	25.16 (10.82,62.12)	-9.24(-10.85, -7.61)	1992-2021
**Ethiopia**	196762.40 (65894.62,430306.07)	201.47 (73.72,432.49)	532213.74 (162983.73,1240152.94)	351.94 (110.65,812.74)	0.71(-2.31,3.81)	1992-2021
**Fiji**	-	-	-	-	-	-
**Finland**	-	-	-	-	-	-
**France**	-	-	-	-	-7.23(-11.56, -2.70)	1992-1991, 1993-2007, 2009-2016
**Gabon**	64435.26 (30176.30,107310.17)	4839.05 (2253.98,8355.67)	47749.68 (17694.33,104632.23)	2640.51 (964.17,6050.15)	-2.09(-2.63, -1.54)	1992-2021
**Gambia**	42100.96 (14168.33,79147.84)	3405.37 (1095.29,6254.30)	30266.28 (19870.05,48224.94)	1475.35 (982.71,2341.47)	-1.79(-3.57,0.01)	1992-2021
**Georgia**	-	-	-	-	-1.29(-7.42,5.24)	1996-2009
**Germany**	589.10 (320.96,948.69)	0.60 (0.32,0.96)	-	-	-14.60(-27.74,0.92)	1992-1999, 2003
**Ghana**	1375434.53 (708482.46,2249915.31)	6220.50 (3123.35,10375.88)	1064315.86 (520066.64,1857997.41)	2882.44 (1395.62,5267.95)	-2.36(-3.30, -1.41)	1992-2021
**Greece**	-	-	-	-	-	2009
**Greenland**	-	-	-	-	-	-
**Grenada**	-	-	-	-	-	-

**Guam**	-	-	-	-	-	-
**Guatemala**	11709.44 (9729.00,14466.31)	152.14 (126.91,187.65)	0.91 (0.49,1.47)	0.01 (0.00,0.01)	-20.73(-23.62, -17.74)	1992-2021
**Guinea**	696741.57 (367531.53,1322966.35)	6272.49 (3315.32,11867.26)	981763.44 (439535.65,1804768.25)	5442.20 (2381.22,10415.47)	-0.33(-0.87,0.22)	1992-2021
**Guinea-Bissau**	103435.82 (57988.63,190324.46)	6287.05 (3488.66,11597.46)	42893.97 (11120.66,93932.48)	1768.55 (472.91,3930.74)	-6.29(-7.30, -5.26)	1992-2021
**Guyana**	19316.62 (7534.79,39906.19)	2337.06 (893.49,4900.02)	3396.23 (1540.57,5765.26)	433.23 (196.85,734.98)	-6.33(-8.15, -4.46)	1992-2021
**Haiti**	91918.34 (39425.79,188454.38)	1218.10 (499.42,2530.44)	42231.01 (5255.41,136131.26)	316.08 (39.15,1025.30)	-5.39(-7.16, -3.58)	1992-2021
**Honduras**	1472.25 (901.70,2800.03)	26.87 (16.22,51.07)	62.40 (7.43,152.49)	0.60 (0.07,1.46)	-12.58(-14.07, -11.07)	1992-2021
**Hungary**	-	-	-	-	-	-
**Iceland**	-	-	-	-	-	-
**India**	6701222.57 (2841347.59,17629658.79)	602.58 (256.08,1579.47)	1148456.10 (43818.92,4188423.38)	91.00 (3.17,329.10)	-5.72(-6.79, -4.64)	1992-2021
**Indonesia**	260055.01 (19648.76,1425980.48)	133.33 (9.67,740.63)	79957.41 (15082.73,324824.84)	28.46 (5.41,115.30)	-4.56(-6.08, -3.02)	1992-2021
**Iran (Islamic Republic of)**	25631.93 (1884.74,207958.00)	37.56 (2.83,304.31)	282.80 (184.06,393.56)	0.32 (0.21,0.45)	-15.64(-17.18, -14.07)	1992-2021
**Iraq**	775.57 (623.98,970.06)	4.14 (3.38,5.07)	-	-	-20.21(-28.28, -11.23)	1992-2009
**Ireland**	-	-	-	-	-	-
**Israel**	-	-	-	-	-	-
**Italy**	-	-	-	-	-	-
**Jamaica**	-	-	-	-	-	-
**Japan**	-	-	-	-	-	-
**Jordan**	-	-	-	-	-	-
**Kazakhstan**	-	-	-	-	-	-
**Kenya**	1129886.04 (718988.12,1836862.79)	2989.87 (1829.57,5046.44)	570872.08 (347113.05,879532.92)	1071.56 (650.21,1665.48)	-7.10(-8.68, -5.49)	1992-2021
**Kiribati**	-	-	-	-	-	-
**Kuwait**	-	-	-	-	-	-
**Kyrgyzstan**	-	-	-	-	-18.73(-29.47, -6.34)	1996-2014
**Lao People's Democratic Republic**	11335.56 (5179.93,28374.94)	229.50 (103.03,570.80)	633.14 (417.86,919.31)	8.25 (5.47,11.91)	-9.32(-11.92, -6.64)	1992-2021
**Latvia**	-	-	-	-	-	-

**Lebanon**	-	-	-	-	-	-
**Lesotho**	-	-	-	-	-	-
**Liberia**	403798.43 (179182.55,717290.69)	13800.21 (6010.40,24676.40)	386647.19 (138504.71,728450.77)	6836.60 (2425.73,13296.02)	-3.20(-3.74, -2.66)	1992-2021
**Libya**	-	-	-	-	-	-
**Lithuania**	-	-	-	-	-	-
**Luxembourg**	-	-	-	-	-	-
**Madagascar**	468669.48 (274383.56,740666.06)	2616.42 (1512.11,4140.81)	482453.99 (119421.56,1031765.00)	1501.44 (379.16,3202.25)	-2.45(-3.25, -1.64)	1992-2021
**Malawi**	1912051.25 (1205957.57,3029253.70)	11106.27 (6961.73,17798.18)	625023.84 (256368.48,1230750.96)	2871.58 (1160.10,5962.09)	-5.81(-6.23, -5.39)	1992-2021
**Malaysia**	5121.69 (2567.22,10293.64)	26.11 (12.92,52.66)	17.72 (11.15,24.68)	0.05 (0.03,0.07)	-20.46(-22.60, -18.26)	1992-2021
**Maldives**	-	-	-	-	-	-
**Mali**	1627121.19 (892976.44,2960367.62)	10794.94 (5834.49,19602.59)	1880800.67 (915891.08,3657459.56)	5353.07 (2601.63,10694.81)	-2.18(-2.55, -1.82)	1992-2021
**Malta**	-	-	-	-	-	-
**Marshall Islands**	-	-	-	-	-	-
**Mauritania**	22762.03 (3023.70,61551.10)	832.84 (117.16,2285.70)	23793.64 (5008.12,50188.97)	531.98 (114.04,1131.82)	-2.01(-4.40,0.44)	1992-2021
**Mauritius**	-	-	-	-	-	-
**Mexico**	1433.53 (1218.97,1722.41)	1.59 (1.37,1.86)	111.44 (91.39,136.74)	0.08 (0.07,0.10)	-10.30(-10.91, -9.68)	1992-2021
**Micronesia (Federated States of)**	-	-	-	-	-	-
**Monaco**	-	-	-	-	-	-
**Mongolia**	-	-	-	-	-	-
**Montenegro**	-	-	-	-	-	-
**Morocco**	162.69 (95.31,245.64)	0.55 (0.33,0.84)	-	-	-35.75(-39.92, -31.30)	1992-2004
**Mozambique**	2431024.87 (1452755.73,4015460.80)	12638.52 (7442.38,21131.02)	1860457.77 (658984.77,3898523.66)	5637.63 (1872.76,12272.54)	-3.53(-3.89, -3.18)	1992-2021
**Myanmar**	150162.09 (68992.04,376978.17)	326.87 (148.73,816.21)	26582.16 (16674.98,60881.94)	47.32 (29.75,108.55)	-4.07(-7.52, -0.50)	1992-2021
**Namibia**	8426.46 (764.16,33692.10)	545.77 (46.71,2197.03)	2522.09 (835.34,11378.54)	97.75 (32.35,441.74)	-9.48(-13.49, -5.28)	1992-2021
**Nauru**	-	-	-	-	-	-
**Nepal**	15141.38 (9243.80,24196.87)	53.89 (33.57,85.68)	665.80 (367.50,1155.04)	2.12 (1.17,3.69)	-11.38(-13.53, -9.17)	1992-2021

**Netherlands**	-	-	-	-	-	-
**New Zealand**	-	-	-	-	-	-
**Nicaragua**	1756.70 (945.95,3996.38)	33.13 (18.27,73.62)	5147.72 (135.03,8885.64)	76.36 (2.03,131.60)	-7.15(-11.77, -2.29)	1992-2021
**Niger**	1500340.66 (656673.30,3015340.25)	10390.27 (4362.22,20823.76)	2549575.86 (956589.89,4503770.50)	7344.92 (2624.86,13447.18)	-0.90(-1.31, -0.49)	1992-2021
**Nigeria**	12123884.32 (6310618.67,20979503.85)	9068.15 (4752.65,15646.26)	16497677.19 (6034839.68,32747629.87)	6016.99 (2146.43,12367.83)	-1.84(-2.21, -1.48)	1992-2021
**Niue**	-	-	-	-	-	-
**North Macedonia**	-	-	-	-	-	-
**Northern Mariana Islands**	-	-	-	-	-	-
**Norway**	-	-	-	-	-	-
**Oman**	4463.64 (1547.40,8624.46)	241.29 (82.11,470.69)	69.54 (46.94,98.72)	1.38 (0.93,1.99)	-10.37(-15.73, -4.66)	1992-2004, 2006-2021
**Pakistan**	635858.06 (37528.85,2913251.15)	449.45 (27.28,2138.16)	635781.35 (56465.72,1770082.24)	239.57 (21.51,671.50)	-2.12(-3.55, -0.68)	1992-2021
**Palau**	-	-	-	-	-	-
**Palestine**	-	-	-	-	-	-
**Panama**	139.99 (54.35,279.37)	5.41 (2.07,10.83)	48.81 (16.27,121.16)	1.16 (0.39,2.83)	-6.70(-9.98, -3.30)	1992-2021
**Papua New Guinea**	141251.90 (32440.77,473196.06)	3126.23 (682.07,10807.00)	132839.99 (59402.70,243405.39)	1259.20 (549.97,2352.77)	-2.75(-3.64, -1.86)	1992-2021
**Paraguay**	128.66 (93.98,179.42)	2.86 (2.14,3.88)	-	-	-19.87(-25.13, -14.23)	1992-2014
**Peru**	54174.52 (12859.47,147868.89)	222.50 (52.57,604.71)	1818.31 (524.31,7044.89)	4.99 (1.46,19.13)	-13.03(-15.26, -10.73)	1992-2021
**Philippines**	28234.46 (1849.35,189261.70)	38.30 (2.54,262.32)	1571.03 (135.50,6278.23)	1.36 (0.12,5.43)	-9.29(-10.54, -8.03)	1992-2021
**Poland**	-	-	-	-	-	-
**Portugal**	-	-	-	-	7.65(-16.94,39.51)	1993, 1996, 1999, 2001
**Puerto Rico**	-	-	-	-	-	-
**Qatar**	-	-	-	-	-	-
**Republic of Korea**	2019.21 (1618.58,2502.72)	4.26 (3.42,5.27)	62.01 (51.10,76.51)	0.10 (0.08,0.13)	-12.84(-15.59, -10.01)	1992-2021
**Republic of Moldova**	-	-	-	-	-	-

**Romania**	-	-	-	-	-	2015
**Russian Federation**	-	-	-	-	-	-
**Rwanda**	803795.48 (391771.24,1566537.74)	7348.77 (3442.33,14859.36)	219026.68 (126756.52,370125.84)	1455.25 (840.64,2513.94)	-7.10(-8.17, -6.01)	1992-2021
**Saint Kitts and Nevis**	-	-	-	-	-	-
**Saint Lucia**	-	-	-	-	-	-
**Saint Vincent and the Grenadines**	-	-	-	-	-	-
**Samoa**	-	-	-	-	-	-
**San Marino**	-	-	-	-	-	-
**Sao Tome and Principe**	2604.20 (386.35,6953.78)	1993.86 (279.38,5412.08)	678.76 (316.90,1067.34)	292.22 (138.82,456.47)	-9.48(-11.92, -6.97)	1992-2021
**Saudi Arabia**	5008.07 (1641.70,10241.44)	30.26 (9.64,62.07)	423.65 (278.60,600.71)	1.00 (0.65,1.41)	-12.64(-15.16, -10.04)	1992-2021
**Senegal**	422249.33 (119297.30,866095.57)	3991.14 (1071.48,8392.38)	290739.30 (126530.95,424809.51)	1831.71 (816.83,2725.03)	-5.17(-6.30, -4.03)	1992-2021
**Serbia**	-	-	-	-	-	-
**Seychelles**	-	-	-	-	-	-
**Sierra Leone**	727075.26 (292991.91,1301171.74)	12251.16 (4861.98,21885.28)	894693.65 (311690.72,1715468.41)	8940.31 (3029.75,17358.47)	-1.95(-2.41, -1.50)	1992-2021
**Singapore**	-	-	-	-	-	1998
**Slovakia**	-	-	-	-	-	-
**Slovenia**	-	-	-	-	-	-
**Solomon Islands**	12427.32 (2190.35,39096.52)	4875.02 (733.31,16034.10)	6857.16 (4302.20,11665.58)	1432.10 (870.71,2482.40)	-5.40(-6.62, -4.15)	1992-2021
**Somalia**	398162.50 (162318.11,886664.79)	3561.16 (1472.48,8025.14)	171168.17 (70089.01,385719.75)	696.36 (288.13,1600.16)	-7.87(-9.16, -6.57)	1992-2021
**South Africa**	8587.18 (1901.62,25432.16)	21.10 (4.60,62.87)	4002.05 (1556.68,14794.28)	6.79 (2.62,25.11)	-5.83(-7.80, -3.83)	1992-2021
**South Sudan**	368853.68 (182835.28,666487.49)	4024.34 (1997.92,7352.18)	607029.06 (263427.11,1147741.40)	4423.00 (1868.65,8462.20)	-0.25(-0.80,0.31)	1992-2021
**Spain**	-	-	-	-	59.53(38.40,83.89)	1995, 1997-1998
**Sri Lanka**	50846.27 (22261.76,123624.88)	300.29 (130.32,736.58)	-	-	-35.21(-38.36, -31.89)	1992-2014
**Sudan**	337214.58 (166176.91,531922.56)	1244.91 (590.55,2009.02)	286336.21 (118658.77,616677.83)	595.33 (239.50,1302.78)	-4.38(-5.36, -3.39)	1992-2021
**Suriname**	1279.04 (91.34,6928.01)	311.36 (22.04,1698.69)	16.25 (5.40,36.11)	2.87 (0.92,6.45)	-19.87(-23.28, -16.32)	1992-2021
**Sweden**	-	-	-	-	-	-

**Switzerland**	-	-	-	-	-	-
**Syrian Arab Republic**	215.66 (157.38,278.75)	1.59 (1.16,2.04)	-	-	-22.66(-31.30, -12.94)	1992-2004
**Taiwan (Province of China)**	-	-	-	-	-	-
**Tajikistan**	24851.68 (2048.33,85067.64)	266.65 (22.82,930.93)	-	-	-32.91(-39.57, -25.51)	1992-2014
**Thailand**	50476.58 (21447.03,102904.78)	84.06 (36.30,170.80)	343.71 (88.39,516.31)	0.56 (0.13,0.86)	-15.16(-17.08, -13.19)	1992-2021
**Timor-Leste**	5032.97 (871.96,17282.53)	463.56 (82.22,1590.49)	-	-	-21.43(-29.55, -12.37)	1992-2018, 2020
**Togo**	344740.07 (173694.46,587005.84)	6450.31 (3256.25,11172.41)	371317.24 (139612.29,673434.86)	4325.44 (1605.32,8066.96)	-1.09(-1.65, -0.53)	1992-2021
**Tokelau**	-	-	-	-	-	-
**Tonga**	-	-	-	-	-	-
**Trinidad and Tobago**	-	-	-	-	-	-
**Tunisia**	-	-	-	-	-	-
**Turkey**	2343.22 (1886.77,2947.68)	3.92 (3.17,4.88)	-	-	-8.50(-11.08, -5.84)	1992-2009
**Turkmenistan**	56.71 (49.67,64.95)	1.59 (1.43,1.79)	-	-	-5.50(-6.31, -4.69)	1992-1994, 1996-2006
**Tuvalu**	-	-	-	-	-	-
**Uganda**	3626602.83 (2324362.18,5684109.64)	10957.79 (6810.68,17614.66)	3658758.93 (1288958.22,6372018.80)	6008.63 (2044.84,11064.79)	-4.86(-5.70, -4.01)	1992-2021
**Ukraine**	-	-	-	-	-	-
**United Arab Emirates**	-	-	-	-	-	-
**United Kingdom**	-	-	-	-	-	-
**United Republic of Tanzania**	2869151.12 (1469873.52,4828735.95)	6254.94 (3175.24,10516.78)	1265202.60 (438778.92,2520884.39)	1623.13 (570.49,3334.91)	-6.16(-6.96, -5.35)	1992-2021
**United States Virgin Islands**	-	-	-	-	-	-
**United States of America**	-	-	-	-	-	-
**Uruguay**	-	-	-	-	-	-
**Uzbekistan**	221.82 (159.87,292.67)	1.01 (0.73,1.33)	-	-	-16.47(-25.06, -6.89)	1992-1994, 1996-2014
**Vanuatu**	1413.43 (559.31,3159.13)	1307.62 (490.74,2996.90)	26.76 (19.67,35.67)	8.25 (6.08,11.03)	-14.19(-18.40, -9.76)	1992-2021

**Venezuela (Bolivarian Republic of)**	4228.74 (1743.95,8203.06)	19.85 (8.18,38.59)	21717.94 (5712.28,57861.76)	86.74 (22.71,231.50)	7.47(4.55,10.47)	1992-2021
**Viet Nam**	48070.23 (7817.73,167502.27)	67.60 (10.84,235.93)	269.37 (176.83,398.34)	0.27 (0.18,0.41)	-15.68(-17.89, -13.42)	1992-2021
**Yemen**	365449.98 (134905.90,803156.56)	2662.66 (928.27,5951.26)	489291.27 (102086.49,1085800.07)	1641.84 (318.61,3882.07)	-1.63(-2.91, -0.33)	1992-2021
**Zambia**	643235.01 (363039.85,1115895.28)	5402.45 (3009.20,9335.67)	487965.32 (149987.65,1014230.33)	2216.20 (657.01,4779.88)	-4.57(-5.45, -3.69)	1992-2021
**Zimbabwe**	131594.53 (48270.75,266564.71)	920.71 (327.74,1904.14)	138078.17 (59838.41,276597.18)	685.30 (298.00,1379.26)	0.28(-1.29,1.87)	1992-2021

Abbreviations: DALY=disability-adjusted life-years. ASR=age-standardized rate. EAPC=estimated annual percentage change. CI=confidence interval.

Table S7. Age-standardized rates of DALY in 2021, and their estimated annual percentage changes from 1992 to 2021 for dengue in all ages, by country.

	1992		2021		1992 - 2021	Included years
**Location**	**DALY Number**	**DALY ASR, per 100 000**	**DALY Number**	**DALY ASR, per 100 000**	**EAPC 95%CI**	
**Afghanistan**	4.41 (1.98,10.82)	0.04 (0.02,0.10)	9.08 (1.67,44.55)	0.05 (0.01,0.17)	0.86(0.74,0.97)	1992-2021
**Albania**	-	-	-	-	-	-
**Algeria**	-	-	-	-	-	-
**American Samoa**	10.50 (2.57,36.46)	20.39 (5.17,70.04)	11.65 (2.55,41.48)	23.64 (5.37,83.22)	0.46(0.08,0.85)	1992-2021
**Andorra**	-	-	-	-	-	-
**Angola**	43.19 (3.18,241.76)	0.39 (0.03,2.20)	762.99 (26.00,4872.43)	2.30 (0.08,14.73)	7.48(6.87,8.09)	1992-2021
**Antigua and Barbuda**	1.61 (0.39,3.64)	2.56 (0.63,5.82)	0.62 (0.14,1.59)	0.69 (0.15,1.78)	-5.35(-6.09, -4.60)	1992-2021
**Argentina**	412.04 (18.95,1353.87)	1.21 (0.06,3.98)	819.76 (149.56,2210.89)	1.80 (0.33,4.85)	1.93(1.43,2.43)	1992-2021
**Armenia**	-	-	-	-	-	-
**Australia**	60.85 (3.64,190.20)	0.35 (0.02,1.11)	191.74 (49.82,498.28)	0.74 (0.19,1.92)	3.96(3.03,4.91)	1992-2021
**Austria**	-	-	-	-	-	-
**Azerbaijan**	-	-	-	-	-	-
**Bahamas**	31.35 (0.58,103.77)	11.60 (0.22,38.24)	51.10 (0.89,185.98)	13.29 (0.25,48.46)	0.49(0.38,0.59)	1992-2021
**Bahrain**	0.92 (0.35,1.85)	0.25 (0.11,0.45)	1.30 (0.45,2.51)	0.12 (0.05,0.23)	-2.97(-3.22, -2.73)	1992-2021
**Bangladesh**	3000.60 (170.36,8806.87)	2.99 (0.42,8.03)	7326.55 (1399.45,17115.41)	4.82 (1.13,10.87)	2.05(1.51,2.59)	1992-2021
**Barbados**	44.40 (5.66,124.19)	17.45 (2.26,48.97)	68.48 (13.95,193.48)	23.00 (4.68,64.71)	1.84(0.22,3.49)	1992-2021
**Belarus**	-	-	-	-	-	-
**Belgium**	-	-	-	-	-	-
**Belize**	7.69 (0.71,25.55)	3.79 (0.34,12.56)	20.67 (2.28,65.37)	4.77 (0.53,15.01)	1.49(0.25,2.73)	1992-2021
**Benin**	196.73 (1.26,1201.07)	3.70 (0.02,22.27)	639.04 (8.65,4594.96)	4.63 (0.07,33.04)	1.48(1.12,1.85)	1992-2021
**Bermuda**	-	-	-	-	-	-
**Bhutan**	38.94 (14.39,78.63)	6.19 (2.48,11.60)	75.76 (22.63,170.74)	11.27 (3.49,24.39)	2.49(1.51,3.47)	1992-2021
**Bolivia**	301.97 (14.32,971.26)	4.34 (0.19,13.89)	873.29 (172.10,2492.70)	7.41 (1.49,21.16)	2.89(1.39,4.42)	1992-2021

**Bosnia and Herzegovina**	-	-	-	-	-	-
**Botswana**	-	-	-	-	-	-
**Brazil**	71428.90 (7805.36,178525.72)	45.75 (5.05,114.40)	141380.46 (49301.57,303154.78)	64.77 (22.45,138.93)	2.16(1.56,2.78)	1992-2021
**Brunei Darussalam**	5.16 (1.45,12.64)	5.62 (1.62,14.31)	10.20 (3.54,23.82)	4.61 (1.51,10.62)	0.24(-0.53,1.02)	1992-2021
**Bulgaria**	-	-	-	-	-	-
**Burkina Faso**	65.42 (2.07,365.12)	0.64 (0.01,3.62)	343.10 (19.04,2032.11)	1.50 (0.08,8.87)	4.13(3.01,5.26)	1992-2021
**Burundi**	267.71 (0.84,1127.60)	4.55 (0.02,18.98)	26.58 (1.46,154.17)	0.20 (0.01,1.16)	-11.54(-14.68, -8.29)	1992-2021
**Cabo Verde**	210.80 (0.00,970.18)	55.01 (0.00,252.81)	413.10 (0.59,2365.08)	73.73 (0.11,422.12)	1.09(0.47,1.71)	1992-2021
**Cambodia**	2901.31 (1254.90,6293.63)	19.23 (9.10,38.52)	3564.76 (1410.36,7120.06)	20.47 (8.27,40.50)	-0.72(-2.95,1.55)	1992-2021
**Cameroon**	242.23 (2.06,1664.13)	2.15 (0.01,14.74)	1106.50 (21.44,8436.02)	3.40 (0.07,25.72)	1.45(1.23,1.67)	1992-2021
**Canada**	-	-	-	-	-	-
**Central African Republic**	63.20 (1.05,439.38)	2.20 (0.04,15.34)	135.65 (1.32,955.63)	2.44 (0.03,17.29)	0.37(0.15,0.60)	1992-2021
**Chad**	22.66 (1.15,144.61)	0.35 (0.01,2.25)	90.73 (4.35,727.76)	0.50 (0.02,4.07)	0.52(0.18,0.86)	1992-2021
**Chile**	-	-	-	-	-	-
**China**	3528.35 (2319.62,4753.07)	0.32 (0.21,0.44)	621.61 (275.39,1188.32)	0.05 (0.02,0.10)	-5.55(-6.05, -5.05)	1992-2021
**Colombia**	5042.38 (2059.96,10275.99)	14.16 (5.56,29.28)	11140.33 (4328.31,20793.90)	23.86 (9.40,43.61)	2.66(0.98,4.37)	1992-2021
**Comoros**	605.21 (138.49,2040.14)	122.73 (28.50,416.84)	883.35 (0.12,6116.90)	117.57 (0.02,809.67)	-1.23(-1.60, -0.86)	1992-2021
**Congo**	96.06 (7.40,489.17)	3.70 (0.28,19.05)	163.24 (5.60,1336.73)	2.99 (0.11,24.24)	-0.21(-0.49,0.08)	1992-2021
**Cook Islands**	0.10 (0.05,0.18)	0.60 (0.28,1.08)	0.23 (0.05,0.83)	1.25 (0.23,4.64)	3.20(1.97,4.45)	1992-2021
**Costa Rica**	810.95 (152.61,2104.29)	24.76 (4.79,64.19)	1218.95 (153.29,3491.45)	25.79 (3.22,74.09)	1.10(-0.65,2.89)	1992-2021
**Coted'Ivoire**	232.02 (2.86,1453.35)	1.75 (0.02,10.96)	630.83 (16.87,4997.97)	2.26 (0.07,17.91)	1.12(0.89,1.35)	1992-2021
**Croatia**	-	-	-	-	-	-
**Cuba**	199.01 (73.42,461.56)	1.82 (0.67,4.22)	195.53 (30.88,596.48)	1.74 (0.27,5.33)	0.38(-0.31,1.07)	1992-2021
**Cyprus**	-	-	-	-	-	-
**Czechia**	-	-	-	-	-	-
**Democratic People's Republic of Korea**	-	-	-	-	-	-

**Democratic Republic of the Congo**	586.94 (35.66,4105.11)	1.46 (0.09,10.16)	1485.75 (44.13,9986.29)	1.64 (0.05,10.99)	0.22(0.14,0.30)	1992-2021
**Denmark**	-	-	-	-	-	-
**Djibouti**	553.48 (6.19,1683.84)	110.15 (1.28,334.74)	76.83 (0.43,589.76)	6.10 (0.05,46.88)	-11.13(-13.27, -8.92)	1992-2021
**Dominica**	2.46 (0.11,7.26)	3.39 (0.16,10.03)	1.47 (0.15,4.81)	2.17 (0.22,7.15)	0.18(-2.45,2.87)	1992-2021
**Dominican Republic**	434.09 (102.02,1094.41)	5.20 (1.07,13.78)	714.64 (127.05,1766.39)	6.56 (1.16,16.19)	1.33(-0.99,3.71)	1992-2021
**Ecuador**	454.76 (151.49,1028.33)	4.11 (1.31,9.47)	1263.52 (425.33,2746.02)	7.04 (2.41,15.25)	0.95(-0.61,2.54)	1992-2021
**Egypt**	384.89 (155.56,902.22)	0.56 (0.23,1.39)	292.59 (22.57,1758.30)	0.28 (0.03,1.67)	-3.05(-3.71, -2.39)	1992-2021
**El Salvador**	748.03 (202.57,1890.48)	12.46 (2.99,32.96)	1248.52 (334.94,2785.46)	19.28 (5.21,42.89)	1.63(0.38,2.89)	1992-2021
**Equatorial Guinea**	0.64 (0.12,3.06)	0.14 (0.03,0.67)	19.03 (0.62,148.94)	1.24 (0.04,9.70)	10.55(8.55,12.59)	1992-2021
**Eritrea**	77.45 (0.60,313.08)	2.43 (0.03,9.83)	8.18 (0.29,52.27)	0.12 (0.00,0.80)	-10.90(-13.14, -8.61)	1992-2021
**Estonia**	-	-	-	-	-	-
**Eswatini**	-	-	-	-	-	-
**Ethiopia**	3159.67 (158.11,11061.22)	5.33 (0.22,19.28)	1448.85 (806.20,2638.40)	0.96 (0.53,1.75)	-5.05(-7.81, -2.22)	1992-2021
**Fiji**	146.07 (37.60,354.07)	18.66 (5.00,44.18)	219.41 (77.53,442.77)	23.98 (8.49,48.14)	0.80(0.37,1.23)	1992-2021
**Finland**	-	-	-	-	-	-
**France**	0.42 (0.20,0.67)	0.00 (0.00,0.00)	1.05 (0.34,1.99)	0.00 (0.00,0.00)	3.05(1.63,4.49)	1992-2021
**Gabon**	41.96 (2.08,277.72)	4.01 (0.21,26.73)	78.92 (1.33,632.72)	4.29 (0.08,34.07)	0.12(-0.28,0.53)	1992-2021
**Gambia**	29.28 (0.26,175.54)	2.70 (0.02,16.13)	114.08 (3.66,845.80)	4.65 (0.16,34.10)	1.30(0.84,1.77)	1992-2021
**Georgia**	-	-	-	-	-	-
**Germany**	44.36 (13.04,93.83)	0.03 (0.01,0.07)	-	-	4.49(-29.08,53.94)	1992-1994
**Ghana**	657.35 (1.98,4607.76)	4.11 (0.01,28.33)	2305.10 (36.35,17034.01)	6.65 (0.11,48.94)	1.30(0.86,1.74)	1992-2021
**Greece**	0.16 (0.09,0.24)	0.00 (0.00,0.00)	0.27 (0.08,0.57)	0.00 (0.00,0.00)	0.65(-0.62,1.95)	1992-2021
**Greenland**	-	-	-	-	-	-
**Grenada**	0.81 (0.14,2.22)	0.91 (0.15,2.46)	3.35 (0.91,8.58)	3.31 (0.91,8.52)	4.33(3.97,4.69)	1992-2021
**Guam**	0.08 (0.04,0.16)	0.08 (0.04,0.14)	0.19 (0.08,0.39)	0.09 (0.04,0.17)	1.46(-0.13,3.07)	1992-2021
**Guatemala**	325.53 (168.43,578.57)	3.20 (1.60,5.88)	4486.87 (2225.61,7545.80)	29.17 (14.62,48.43)	9.94(8.39,11.51)	1992-2021

**Guinea**	54.14 (1.62,510.61)	0.82 (0.02,7.87)	160.09 (4.42,920.14)	1.18 (0.03,6.78)	1.17(0.04,2.31)	1992-2021
**Guinea-Bissau**	25.13 (0.28,146.82)	2.36 (0.02,13.98)	82.15 (0.45,580.60)	3.91 (0.02,27.37)	1.61(1.41,1.81)	1992-2021
**Guyana**	20.55 (3.51,55.98)	2.67 (0.49,7.25)	33.21 (7.83,81.01)	4.37 (1.03,10.65)	3.52(1.67,5.40)	1992-2021
**Haiti**	222.04 (11.70,1023.02)	3.23 (0.15,15.18)	562.89 (27.00,2796.67)	4.36 (0.21,21.61)	-0.13(-1.02,0.77)	1992-2021
**Honduras**	824.19 (345.31,1661.08)	17.06 (7.20,33.58)	2840.22 (831.50,6161.74)	29.45 (9.36,62.88)	2.84(1.24,4.46)	1992-2021
**Hungary**	-	-	-	-	-	-
**Iceland**	-	-	-	-	-	-
**India**	375900.91 (185993.40,610875.21)	43.05 (21.19,69.38)	841615.98 (282570.71,1498205.19)	62.77 (21.96,110.46)	1.90(1.61,2.18)	1992-2021
**Indonesia**	610495.41 (389675.91,957140.90)	284.97 (186.78,437.65)	669642.90 (402102.04,971681.35)	279.79 (170.93,404.43)	0.29(0.12,0.47)	1992-2021
**Iran (Islamic Republic of)**	-	-	-	-	-	-
**Iraq**	-	-	-	-	-	-
**Ireland**	-	-	-	-	-	-
**Israel**	-	-	-	-	-	-
**Italy**	-	-	-	-	-	-
**Jamaica**	71.90 (8.44,185.53)	2.88 (0.33,7.43)	80.30 (27.63,175.56)	2.92 (1.00,6.32)	2.92(0.58,5.32)	1992-2021
**Japan**	24.39 (18.56,29.09)	0.03 (0.02,0.04)	7.10 (3.83,10.17)	0.01 (0.00,0.01)	-3.57(-4.22, -2.91)	1992-2021
**Jordan**	5.47 (1.85,15.32)	0.14 (0.05,0.38)	21.45 (3.78,76.80)	0.18 (0.04,0.63)	3.71(0.87,6.63)	1992-2021
**Kazakhstan**	-	-	-	-	-	-
**Kenya**	6187.75 (409.51,19336.94)	24.71 (1.61,76.94)	1646.92 (26.08,9368.31)	3.20 (0.05,18.42)	-7.11(-9.29, -4.88)	1992-2021
**Kiribati**	50.55 (8.09,167.47)	65.80 (11.18,215.97)	75.09 (6.07,320.81)	62.41 (5.61,262.50)	-1.01(-1.48, -0.55)	1992-2021
**Kuwait**	17.74 (4.39,44.91)	1.06 (0.26,2.67)	26.32 (1.35,101.59)	0.59 (0.03,2.26)	0.55(-2.17,3.35)	1992-2021
**Kyrgyzstan**	-	-	-	-	-	-
**Lao People's Democratic Republic**	784.89 (352.03,1740.06)	13.42 (6.15,27.09)	1121.24 (400.69,2238.96)	14.39 (5.07,28.34)	0.95(-0.48,2.41)	1992-2021
**Latvia**	-	-	-	-	-	-
**Lebanon**	6.86 (1.19,19.45)	0.22 (0.04,0.63)	18.95 (1.38,67.45)	0.34 (0.02,1.22)	4.27(1.27,7.37)	1992-2021
**Lesotho**	-	-	-	-	-	-

**Liberia**	84.44 (0.65,479.04)	4.11 (0.02,23.41)	296.51 (4.55,2031.81)	5.31 (0.09,36.63)	0.98(0.65,1.30)	1992-2021
**Libya**	-	-	-	-	-	-
**Lithuania**	-	-	-	-	-	-
**Luxembourg**	-	-	-	-	-	-
**Madagascar**	482.62 (2.64,1830.15)	3.78 (0.03,14.24)	107.97 (1.51,834.99)	0.37 (0.01,2.90)	-8.26(-10.47, -6.00)	1992-2021
**Malawi**	1054.09 (150.81,3304.84)	9.41 (1.26,30.80)	819.41 (177.34,2597.89)	3.07 (0.70,9.17)	-3.73(-4.99, -2.46)	1992-2021
**Malaysia**	10097.87 (5339.36,16309.66)	51.43 (26.64,82.70)	18524.04 (9852.58,32846.27)	57.98 (31.11,102.26)	0.79(0.16,1.42)	1992-2021
**Maldives**	258.27 (90.89,572.52)	70.37 (25.80,151.31)	392.76 (106.56,1140.11)	86.34 (27.26,229.39)	1.41(0.63,2.20)	1992-2021
**Mali**	48.67 (2.14,306.94)	0.53 (0.02,3.41)	153.14 (8.44,1355.63)	0.63 (0.03,5.57)	0.90(0.76,1.03)	1992-2021
**Malta**	-	-	-	-	-	-
**Marshall Islands**	37.95 (6.56,157.50)	77.69 (13.62,320.41)	56.69 (5.21,250.49)	100.28 (9.53,442.01)	1.05(0.61,1.50)	1992-2021
**Mauritania**	5.99 (0.11,40.31)	0.27 (0.00,1.86)	18.89 (1.25,134.49)	0.42 (0.03,3.02)	1.97(1.69,2.24)	1992-2021
**Mauritius**	40.57 (4.25,133.31)	3.62 (0.39,12.02)	122.31 (2.73,531.67)	9.83 (0.19,42.90)	3.44(3.02,3.85)	1992-2021
**Mexico**	6894.63 (1084.23,16135.60)	7.62 (1.20,17.78)	14517.95 (9025.38,21849.37)	11.27 (7.01,16.95)	3.84(2.34,5.35)	1992-2021
**Micronesia (Federated States of)**	5.48 (0.96,15.21)	5.30 (1.04,14.11)	4.73 (0.76,14.00)	4.72 (0.83,13.72)	-0.48(-0.77, -0.18)	1992-2021
**Monaco**	-	-	-	-	-	-
**Mongolia**	-	-	-	-	-	-
**Montenegro**	-	-	-	-	-	-
**Morocco**	-	-	-	-	-	-
**Mozambique**	982.85 (23.64,3518.50)	7.15 (0.18,25.33)	361.81 (1.14,2951.01)	1.15 (0.00,9.37)	-5.54(-7.19, -3.86)	1992-2021
**Myanmar**	28153.30 (12118.54,61957.81)	58.10 (25.52,126.19)	26470.48 (10219.41,48321.50)	49.86 (19.22,91.40)	-0.45(-0.60, -0.30)	1992-2021
**Namibia**	-	-	-	-	-	-
**Nauru**	0.07 (0.03,0.14)	0.93 (0.49,1.79)	1.17 (0.02,5.11)	10.68 (0.35,46.54)	10.09(7.25,13.00)	1992-2021
**Nepal**	9975.99 (5628.87,17995.83)	46.91 (29.95,74.94)	14814.24 (6023.40,28795.76)	52.12 (23.00,97.99)	0.74(0.52,0.97)	1992-2021
**Netherlands**	-	-	-	-	-	-
**New Zealand**	-	-	-	-	-	-

**Nicaragua**	316.17 (82.99,817.91)	6.64 (1.56,17.93)	800.68 (262.52,2023.89)	11.93 (3.91,30.11)	1.16(-0.27,2.61)	1992-2021
**Niger**	35.84 (2.25,201.14)	0.41 (0.02,2.35)	95.53 (4.54,791.86)	0.38 (0.02,3.13)	0.17(-0.12,0.47)	1992-2021
**Nigeria**	5190.36 (28.20,18927.42)	5.38 (0.02,19.73)	16733.12 (493.99,66035.72)	7.17 (0.21,28.02)	1.10(0.94,1.25)	1992-2021
**Niue**	0.03 (0.01,0.08)	1.18 (0.28,3.40)	0.03 (0.01,0.07)	1.61 (0.48,4.36)	1.04(0.66,1.43)	1992-2021
**North Macedonia**	-	-	-	-	-	-
**Northern Mariana Islands**	2.58 (0.78,6.50)	5.72 (1.97,14.45)	3.81 (0.76,12.27)	8.02 (1.65,25.83)	0.40(-0.79,1.61)	1992-2021
**Norway**	-	-	-	-	-	-
**Oman**	383.90 (181.77,769.39)	23.80 (13.69,40.09)	295.95 (124.53,585.63)	13.84 (7.42,25.31)	-1.64(-1.80, -1.48)	1992-2021
**Pakistan**	20694.08 (5372.75,45391.31)	17.31 (4.36,37.98)	67835.55 (21616.89,138461.59)	29.82 (9.91,60.29)	2.25(2.08,2.42)	1992-2021
**Palau**	5.98 (0.36,29.29)	37.04 (2.23,181.91)	5.94 (0.07,33.42)	34.75 (0.58,191.60)	-1.38(-2.33, -0.41)	1992-2021
**Palestine**	8.11 (3.96,16.63)	0.37 (0.19,0.76)	15.41 (4.57,49.70)	0.34 (0.12,0.99)	1.54(-0.37,3.48)	1992-2021
**Panama**	144.70 (23.16,409.82)	5.72 (0.92,16.10)	514.95 (176.68,1513.43)	11.96 (4.11,35.16)	2.65(1.85,3.46)	1992-2021
**Papua New Guinea**	22.78 (9.61,40.76)	0.72 (0.29,1.31)	62.97 (26.54,151.38)	0.76 (0.32,1.59)	-0.28(-0.78,0.22)	1992-2021
**Paraguay**	536.47 (11.58,1877.94)	12.30 (0.28,42.90)	2489.10 (994.90,4870.14)	35.62 (14.46,68.67)	6.01(4.80,7.24)	1992-2021
**Peru**	744.22 (144.35,1913.22)	3.21 (0.61,8.35)	2190.39 (660.23,5822.00)	6.05 (1.82,16.06)	3.34(2.60,4.09)	1992-2021
**Philippines**	53566.20 (44858.01,64714.74)	62.43 (52.35,74.60)	147299.83 (110875.56,190016.49)	126.60 (95.17,164.20)	3.74(3.11,4.37)	1992-2021
**Poland**	-	-	-	-	-	-
**Portugal**	-	-	-	-	-	2012-2013
**Puerto Rico**	726.42 (74.02,2629.04)	19.77 (2.02,71.53)	568.87 (18.62,2481.62)	17.01 (0.53,74.55)	-0.26(-0.96,0.44)	1992-2021
**Qatar**	0.50 (0.17,1.00)	0.13 (0.05,0.25)	1.50 (0.35,3.31)	0.07 (0.02,0.13)	-2.35(-2.67, -2.03)	1992-2021
**Republic of Korea**	1.54 (0.79,2.36)	0.01 (0.00,0.01)	0.69 (0.21,1.34)	0.00 (0.00,0.00)	-8.38(-9.19, -7.57)	1992-2021
**Republic of Moldova**	-	-	-	-	-	-
**Romania**	-	-	-	-	-	-
**Russian Federation**	-	-	-	-	-	-
**Rwanda**	371.17 (1.21,1603.97)	4.85 (0.03,20.84)	19.65 (2.07,117.50)	0.15 (0.02,0.88)	-13.10(-15.88, -10.23)	1992-2021
**Saint Kitts and Nevis**	0.38 (0.02,2.18)	0.91 (0.06,5.17)	0.45 (0.04,3.26)	0.81 (0.08,5.71)	0.32(-0.12,0.76)	1992-2021

**Saint Lucia**	2.84 (0.92,6.47)	2.01 (0.66,4.57)	7.08 (2.68,14.33)	4.24 (1.65,8.57)	3.13(2.47,3.80)	1992-2021
**Saint Vincent and the Grenadines**	6.25 (0.68,17.85)	5.55 (0.59,15.82)	3.80 (0.19,12.93)	3.37 (0.17,11.42)	-1.33(-2.31, -0.33)	1992-2021
**Samoa**	4.12 (0.70,11.11)	2.59 (0.48,6.65)	12.32 (3.71,39.12)	5.83 (1.87,18.22)	2.42(1.25,3.60)	1992-2021
**San Marino**	-	-	-	-	-	-
**Sao Tome and Principe**	9.78 (0.09,52.62)	7.50 (0.05,40.80)	17.11 (0.56,128.73)	7.75 (0.26,58.18)	0.14(-0.05,0.32)	1992-2021
**Saudi Arabia**	17.90 (6.78,39.40)	0.34 (0.13,0.72)	183.73 (64.87,376.17)	0.79 (0.30,1.46)	4.84(3.64,6.05)	1992-2021
**Senegal**	35.13 (1.30,161.16)	0.43 (0.01,1.98)	116.43 (19.23,439.28)	0.73 (0.12,2.75)	2.16(1.88,2.44)	1992-2021
**Serbia**	-	-	-	-	-	-
**Seychelles**	61.57 (2.10,300.52)	80.60 (2.69,396.36)	127.53 (24.51,481.10)	124.10 (24.09,466.69)	1.63(1.36,1.91)	1992-2021
**Sierra Leone**	218.03 (1.33,1773.27)	5.17 (0.02,42.00)	454.32 (6.66,3777.47)	5.02 (0.09,42.03)	-0.01(-0.29,0.27)	1992-2021
**Singapore**	2310.18 (399.50,6379.55)	72.41 (12.58,200.32)	4695.23 (857.98,12009.93)	85.30 (15.20,216.32)	1.21(-0.05,2.49)	1992-2021
**Slovakia**	-	-	-	-	-	-
**Slovenia**	-	-	-	-	-	-
**Solomon Islands**	15.44 (0.49,61.29)	4.47 (0.24,16.72)	73.61 (14.87,209.85)	10.86 (2.36,30.35)	4.02(1.95,6.13)	1992-2021
**Somalia**	633.43 (3.97,2298.11)	8.02 (0.07,29.31)	94.33 (2.63,789.63)	0.44 (0.01,3.65)	-10.73(-12.88, -8.52)	1992-2021
**South Africa**	-	-	-	-	-	-
**South Sudan**	438.79 (0.51,1787.27)	7.00 (0.01,28.46)	4.84 (2.00,11.12)	0.05 (0.02,0.11)	-19.60(-23.27, -15.75)	1992-2021
**Spain**	0.25 (0.13,0.38)	0.00 (0.00,0.00)	0.17 (0.06,0.32)	0.00 (0.00,0.00)	-3.61(-4.37, -2.84)	1992-2021
**Sri Lanka**	8193.11 (5267.64,12817.14)	44.97 (28.91,69.95)	12691.08 (7577.35,23644.48)	59.54 (35.34,109.82)	2.32(0.90,3.75)	1992-2021
**Sudan**	30.46 (4.42,114.46)	0.14 (0.02,0.53)	75.19 (3.36,508.70)	0.18 (0.01,1.18)	1.17(0.38,1.97)	1992-2021
**Suriname**	15.34 (3.19,42.99)	3.87 (0.80,10.77)	51.91 (14.81,107.21)	9.23 (2.60,19.62)	0.63(-2.07,3.40)	1992-2021
**Sweden**	-	-	-	-	-	-
**Switzerland**	-	-	-	-	-	-
**Syrian Arab Republic**	7.66 (3.11,21.15)	0.06 (0.02,0.15)	7.14 (1.97,20.18)	0.05 (0.02,0.15)	1.97(-0.82,4.85)	1992-2021
**Taiwan (Province of China)**	328.03 (41.48,1018.67)	1.58 (0.20,4.91)	738.41 (211.06,1823.84)	2.47 (0.59,6.86)	3.23(2.45,4.03)	1992-2021
**Tajikistan**	-	-	-	-	-	-

**Thailand**	26402.19 (15787.25,42785.85)	46.38 (27.27,73.63)	11525.18 (6700.31,18440.21)	20.79 (12.53,32.24)	-2.46(-2.93, -1.99)	1992-2021
**Timor-Leste**	183.16 (82.81,435.91)	15.56 (8.01,33.52)	332.70 (163.44,619.95)	19.96 (9.84,36.76)	0.93(-0.74,2.62)	1992-2021
**Togo**	162.42 (0.73,1267.11)	4.10 (0.01,31.81)	421.15 (0.43,3360.17)	4.97 (0.01,39.40)	0.87(0.62,1.12)	1992-2021
**Tokelau**	0.01 (0.00,0.02)	0.58 (0.25,1.13)	0.01 (0.01,0.03)	0.87 (0.38,2.12)	1.08(0.33,1.85)	1992-2021
**Tonga**	36.43 (14.74,69.82)	37.48 (15.24,67.81)	178.50 (14.87,602.85)	168.65 (14.61,563.73)	6.55(4.82,8.30)	1992-2021
**Trinidad and Tobago**	105.93 (37.11,252.02)	8.60 (3.04,20.19)	292.13 (105.67,558.29)	20.94 (7.62,40.45)	3.33(2.11,4.57)	1992-2021
**Tunisia**	-	-	-	-	-	-
**Turkey**	-	-	-	-	-	-
**Turkmenistan**	-	-	-	-	-	-
**Tuvalu**	0.10 (0.04,0.25)	1.19 (0.54,2.77)	0.59 (0.04,2.64)	4.81 (0.38,21.35)	5.75(4.17,7.36)	1992-2021
**Uganda**	2687.43 (8.25,10661.83)	14.32 (0.07,56.65)	226.40 (7.81,1492.38)	0.51 (0.02,3.40)	-12.36(-14.90, -9.74)	1992-2021
**Ukraine**	-	-	-	-	-	-
**United Arab Emirates**	-	-	-	-	-	-
**United Kingdom**	-	-	-	-	-	-
**United Republic of Tanzania**	4767.48 (4.52,17500.02)	17.10 (0.03,62.85)	119.43 (11.28,804.28)	0.20 (0.02,1.36)	-15.52(-19.14, -11.73)	1992-2021
**United States Virgin Islands**	2.52 (0.10,11.07)	2.38 (0.10,10.38)	1.65 (0.03,10.07)	1.92 (0.03,11.83)	-0.61(-0.91, -0.31)	1992-2021
**United States of America**	6.44 (3.31,11.98)	0.00 (0.00,0.01)	42.09 (15.85,121.12)	0.02 (0.01,0.04)	8.32(7.40,9.24)	1992-2021
**Uruguay**	-	-	-	-	-	-
**Uzbekistan**	-	-	-	-	-	-
**Vanuatu**	4.13 (0.81,10.56)	2.78 (0.65,6.81)	25.42 (6.29,75.16)	8.17 (2.12,23.82)	4.26(3.45,5.08)	1992-2021
**Venezuela (Bolivarian Republic of)**	2749.45 (962.43,6100.69)	13.41 (4.63,29.90)	6305.86 (2256.76,12786.04)	25.01 (8.99,51.00)	2.47(1.54,3.40)	1992-2021
**Viet Nam**	10216.43 (4590.20,19113.53)	12.98 (5.85,23.90)	16031.38 (5654.25,33260.50)	16.84 (6.06,34.57)	0.58(-0.16,1.34)	1992-2021
**Yemen**	24.72 (3.24,110.35)	0.17 (0.02,0.75)	85.51 (9.41,468.44)	0.26 (0.03,1.39)	4.47(0.64,8.44)	1992-2021
**Zambia**	817.03 (3.00,3501.19)	9.59 (0.07,40.91)	91.05 (3.31,558.27)	0.46 (0.02,2.84)	-11.01(-13.62, -8.32)	1992-2021
**Zimbabwe**	14.48 (2.56,65.24)	0.13 (0.02,0.59)	12.28 (1.13,79.37)	0.08 (0.01,0.51)	-2.52(-3.28, -1.76)	1992-2021

Abbreviations: DALY=disability-adjusted life-years. ASR=age-standardized rate. EAPC=estimated annual percentage change. CI=confidence interval.

Table S8. Joinpoint regression analysis results for dengue and malaria burden

disease	Rate	Period	*APC*%（95%CI）	*AAPC*%（95%CI）
malaria	ASIR	1992-2001	0.93 (0.68, 1.19) *	
malaria	ASIR	2001-2010	-0.65 (-0.94, -0.35) *	-0.21 (-0.42, 0.00)
malaria	ASIR	2010-2014	-3.61 (-4.59, -2.62) *	
malaria	ASIR	2014-2021	0.95 (0.52, 1.39) *	
malaria	ASR of DALY	1992-2003	1.45 (1.18, 1.73)	
malaria	ASR of DALY	2003-2010	-2.01 (-2.44, -1.58)	
malaria	ASR of DALY	2010-2013	-6.34 (-8.19, -4.45)	-0.63 (-0.97, -0.29) *
malaria	ASR of DALY	2013-2018	-2.97 (-3.83, -2.10)	
malaria	ASR of DALY	2018-2021	6.32 (4.21, 8.46)	
dengue	ASIR	1992-2004	1.07 (0.90, 1.23) *	
dengue	ASIR	2004-2015	3.79 (3.58, 4.01) *	1.31 (1.07, 1.55) *
dengue	ASIR	2015-2018	-5.58 (-7.01, -4.14) *	
dengue	ASIR	2018-2021	0.92 (-0.60, 2.47)	
dengue	ASR of DALY	1992-2003	1.10 (0.66, 1.55)	
dengue	ASR of DALY	2003-2014	2.68 (2.23, 3.14)	0.82 (0.52, 1.12) *
dengue	ASR of DALY	2014-2021	-2.30 (-2.99, -1.60)	

* indicates a p-value <0.05.

Table S9. Formal comparison of SDI-burden correlations between 1992 and 2021 across 21 GBD regions.

Disease	Metric	r_{1992}	r_{2021}	Δ r	Bootstrap 95% CI	P
Dengue	ASR of DALY	-0.622	-0.497	0.125	-0.053 to 0.349	0.555
Dengue	ASIR	-0.592	-0.521	0.070	-0.151 to 0.347	0.740
Malaria	ASR of DALY	-0.870	-0.849	0.020	-0.094 to 0.140	0.892
Malaria	ASIR	-0.902	-0.848	0.054	-0.066 to 0.205	0.713

Delta r was calculated as r_{2021} - r_{1992}. P values were obtained from paired permutation tests preserving the matched regional structure.

Table S10. Formal comparison of SDI-ASIR and SDI-age-standardized DALY rate correlations within the same year.

Disease	Year	r (SDI, ASIR)	r (SDI, ASR of DALY)	Δ r	Bootstrap 95% CI	P
Dengue	1,992	-0.592	-0.622	0.030	-0.065 to 0.139	0.495
Dengue	2,021	-0.521	-0.497	-0.024	-0.127 to 0.080	0.508
Malaria	1,992	-0.902	-0.870	-0.032	-0.165 to 0.043	0.284
Malaria	2,021	-0.848	-0.849	0.001	-0.083 to 0.084	0.943

Williams tests were applied after rank transformation because the two Spearman correlations shared the same SDI variable and were estimated from the same 21 regions.

Table S11 APC sensitivity analysis using GBD lower-bound estimates: net drift of incidence, 1992–2021.

Group	Net drift value (95% CI)					
	Global	Low SDI	Low-middle SDI	Middle SDI	High-middle SDI	High SDI
**Malaria**						
both	-2.02(-2.67, -1.36)	-1.15(-2.37,0.10)	-3.17(-3.81, -2.52)	-3.88(-4.53, -3.24)	-3.48(-3.65, -3.31)	-13.38(-13.82, -12.95)
females	-1.88(-2.49, -1.26)	-1.20(-2.36, -0.03)	-3.13(-3.73, -2.53)	-3.74(-4.35, -3.13)	-3.37(-3.54, -3.19)	-12.69(-13.19, -12.19)
males	-2.18(-2.87, -1.48)	-1.09(-2.41,0.25)	-3.20(-3.89, -2.51)	-4.01(-4.71, -3.32)	-3.62(-3.80, -3.45)	-14.18(-14.57, -13.78)
**Dengue**						
both	3.58(3.40,3.75)	-13.10(-13.30, -12.90)	1.02(0.88,1.16)	6.32(6.23,6.41)	7.23(7.12,7.34)	4.52(4.34,4.71)
females	3.70(3.53,3.87)	-13.59(-13.77, -13.41)	1.13(1.00,1.27)	6.47(6.38,6.56)	7.31(7.19,7.42)	4.27(4.10,4.43)
males	3.55(3.38,3.72)	-12.81(-12.91, -12.71)	0.90(0.77,1.04)	6.49(6.42,6.56)	7.49(7.39,7.60)	4.40(4.22,4.59)

Net drift reflects the overall annual percentage change in the ASIR from 1992 to 2021.

Table S12 APC sensitivity analysis using GBD upper-bound estimates: net drift of incidence, 1992–2021.

Group	Net drift value (95% CI)					
	Global	Low SDI	Low-middle SDI	Middle SDI	High-middle SDI	High SDI
**Malaria**						
both	-2.08(-2.38, -1.79)	-1.43(-1.96, -0.91)	-3.58(-3.84, -3.33)	-4.02(-4.44, -3.59)	-5.05(-5.83, -4.26)	-13.27(-13.61, -12.92)
females	-1.90(-2.19, -1.60)	-1.43(-1.96, -0.91)	-3.48(-3.73, -3.23)	-3.81(-4.22, -3.40)	-4.87(-5.61, -4.14)	-12.83(-13.22, -12.44)
males	-2.30(-2.60, -2.00)	-1.43(-1.96, -0.90)	-3.69(-3.96, -3.42)	-4.22(-4.67, -3.78)	-7.33(-8.04, -6.62)	-13.83(-14.16, -13.51)
**Dengue**						
both	1.92(1.88,1.96)	1.05(0.90,1.20)	1.58(1.55,1.62)	1.45(1.41,1.50)	2.44(2.34,2.54)	1.66(1.50,1.83)
females	2.05(2.01,2.09)	0.43(0.28,0.58)	1.60(1.57,1.64)	1.48(1.44,1.53)	2.68(2.58,2.78)	1.70(1.53,1.87)
males	1.73(1.68,1.78)	1.09(0.95,1.24)	1.54(1.50,1.57)	1.41(1.36,1.46)	2.31(2.21,2.41)	1.61(1.42,1.79)

Net drift reflects the overall annual percentage change in the ASIR from 1992 to 2021.

Table S13. Candidate BAPC model comparison under the 1992–2011 training and 2012–2021 testing split for malaria.

Sex	Model structure	DIC	RMSE	MAE	MAPE (%)
both	age=rw2, period=rw2, cohort=rw1	7282.95	188.54	158.76	5.00
both	age=rw1, period=rw2, cohort=rw1	7282.94	198.33	165.73	5.22
both	age=rw1, period=rw2, cohort=rw2	7282.36	249.68	200.37	6.32
**both**	**age=rw2, period=rw1, cohort=rw2**	**7282.34**	**192.72**	**176.19**	**5.60**
both	age=rw2, period=rw2, cohort=rw2	7278.60	2733629.77	978017.48	29880.62
both	age=rw2, period=rw1, cohort=rw1	7282.93	455.13	444.58	14.10
both	age=rw1, period=rw1, cohort=rw1	7282.89	466.60	455.57	14.45
both	age=rw1, period=rw1, cohort=rw2	7282.16	541.64	525.54	16.66
female	age=rw2, period=rw2, cohort=rw1	7021.10	161.51	138.99	4.26
female	age=rw1, period=rw2, cohort=rw1	7021.08	170.49	145.50	4.46
female	age=rw1, period=rw2, cohort=rw2	7020.39	228.85	185.65	5.69
**female**	**age=rw2, period=rw1, cohort=rw2**	**7020.43**	**191.58**	**175.82**	**5.44**
female	age=rw2, period=rw2, cohort=rw2	7008.67	1.30×10^{31}	4.12×10^{30}	1.22×10^{29}
female	age=rw2, period=rw1, cohort=rw1	7021.15	433.90	423.97	13.10
female	age=rw1, period=rw1, cohort=rw1	7021.11	444.88	434.60	13.42
female	age=rw1, period=rw1, cohort=rw2	7020.17	576.75	558.27	17.22
male	age=rw2, period=rw2, cohort=rw1	6980.76	223.85	183.68	5.94
male	age=rw1, period=rw2, cohort=rw1	6980.69	232.17	189.58	6.13
**male**	**age=rw2, period=rw1, cohort=rw2**	**6978.96**	**196.56**	**177.95**	**5.80**
male	age=rw1, period=rw2, cohort=rw2	6979.04	273.37	216.94	7.02
male	age=rw1, period=rw1, cohort=rw2	6978.79	460.35	448.70	14.61
male	age=rw2, period=rw2, cohort=rw2	6980.97	17224.19	8958.42	285.03
male	age=rw2, period=rw1, cohort=rw1	6980.63	479.60	468.01	15.24
male	age=rw1, period=rw1, cohort=rw1	6980.54	492.55	480.32	15.64

Light-blue highlighted rows indicate the models selected for the final BAPC projections based on out-of-sample predictive performance, DIC, and numerical stability.

Table S14. Candidate BAPC model comparison under the 1992–2011 training and 2012–2021 testing split for dengue.

Sex	Model structure	DIC	RMSE	MAE	MAPE (%)
**both**	**age=rw1, period=rw1, cohort=rw2**	**7060.21**	**71.15**	**64.73**	**8.24**
both	age=rw2, period=rw1, cohort=rw2	7060.22	76.36	68.41	8.75
both	age=rw2, period=rw1, cohort=rw1	7058.48	83.43	74.03	9.06
both	age=rw1, period=rw1, cohort=rw1	7058.49	84.06	74.83	9.17
both	age=rw2, period=rw2, cohort=rw2	7060.31	149.98	112.33	14.74
both	age=rw1, period=rw2, cohort=rw2	7060.37	150.30	112.53	14.77
both	age=rw2, period=rw2, cohort=rw1	7058.74	163.76	121.04	15.90
both	age=rw1, period=rw2, cohort=rw1	7058.74	163.91	121.13	15.92
**female**	**age=rw1, period=rw1, cohort=rw2**	**6825.58**	**77.16**	**70.51**	**8.30**
female	age=rw2, period=rw1, cohort=rw2	6825.61	83.21	74.97	8.88
female	age=rw2, period=rw1, cohort=rw1	6822.98	96.14	86.64	9.85
female	age=rw1, period=rw1, cohort=rw1	6822.97	96.85	87.51	9.95
female	age=rw2, period=rw2, cohort=rw2	6825.80	169.27	126.61	15.41
female	age=rw1, period=rw2, cohort=rw2	6825.77	169.30	126.63	15.41
female	age=rw2, period=rw2, cohort=rw1	6823.30	185.18	136.68	16.66
female	age=rw1, period=rw2, cohort=rw1	6823.30	185.34	136.78	16.67
male	age=rw2, period=rw1, cohort=rw1	6746.75	70.21	60.33	7.98
**male**	**age=rw1, period=rw1, cohort=rw2**	**6750.33**	**65.91**	**59.57**	**8.24**
male	age=rw1, period=rw1, cohort=rw1	6746.75	70.71	61.00	8.07
male	age=rw2, period=rw1, cohort=rw2	6750.33	69.89	62.26	8.64
male	age=rw1, period=rw2, cohort=rw2	6750.70	129.05	97.24	13.81
male	age=rw2, period=rw2, cohort=rw2	6750.67	129.05	97.24	13.81
male	age=rw2, period=rw2, cohort=rw1	6747.31	139.46	103.83	14.76
male	age=rw1, period=rw2, cohort=rw1	6747.33	139.59	103.91	14.77

Light-blue highlighted rows indicate the models selected for the final BAPC projections based on out-of-sample predictive performance, DIC, and numerical stability.

Table S15. Projected ASIR of malaria and dengue by sex, 2022–2036.

Sex	Year	ASIR of malaria (95% *CI*)	ASIR of dengue (95% *CI*)
Both	2,022	3028.63 (2799.74, 3257.52)	747.24 (729.67, 764.81)
Both	2,023	3043.91 (2774.35, 3313.47)	752.18 (730.24, 774.12)
Both	2,024	3061.49 (2749.00, 3373.98)	757.40 (731.50, 783.31)
Both	2,025	3080.84 (2722.61, 3439.06)	762.80 (733.13, 792.46)
Both	2,026	3101.73 (2694.55, 3508.91)	768.18 (734.82, 801.53)
Both	2,027	3124.58 (2664.84, 3584.33)	773.54 (736.49, 810.59)
Both	2,028	3150.05 (2633.79, 3666.31)	779.20 (738.38, 820.03)
Both	2,029	3177.88 (2600.84, 3754.91)	785.17 (740.45, 829.89)
Both	2,030	3207.74 (2565.34, 3850.14)	791.31 (742.53, 840.09)
Both	2,031	3239.53 (2526.83, 3952.24)	797.47 (744.43, 850.51)
Both	2,032	3273.63 (2485.25, 4062.00)	803.65 (746.11, 861.20)
Both	2,033	3310.58 (2440.74, 4180.43)	810.19 (747.83, 872.55)
Both	2,034	3350.31 (2392.70, 4307.92)	817.11 (749.60, 884.62)
Both	2,035	3392.67 (2340.45, 4444.90)	824.30 (751.27, 897.34)
Both	2,036	3437.72 (2283.38, 4592.05)	831.63 (752.64, 910.61)
Female	2,022	3118.48 (2879.64, 3357.32)	803.29 (784.43, 822.16)
Female	2,023	3133.70 (2852.07, 3415.33)	808.48 (784.71, 832.25)
Female	2,024	3151.37 (2824.11, 3478.64)	814.00 (785.81, 842.19)
Female	2,025	3170.88 (2794.56, 3547.20)	819.71 (787.35, 852.07)
Female	2,026	3191.97 (2762.76, 3621.19)	825.39 (788.95, 861.82)
Female	2,027	3215.19 (2728.80, 3701.59)	831.02 (790.51, 871.53)
Female	2,028	3241.34 (2693.12, 3789.57)	836.99 (792.33, 881.65)
Female	2,029	3270.11 (2655.04, 3885.17)	843.30 (794.36, 892.25)
Female	2,030	3301.08 (2613.78, 3988.38)	849.80 (796.40, 903.20)
Female	2,031	3334.17 (2568.79, 4099.54)	856.29 (798.22, 914.36)
Female	2,032	3369.84 (2520.04, 4219.64)	862.76 (799.75, 925.78)
Female	2,033	3408.81 (2467.67, 4349.94)	869.62 (801.33, 937.92)
Female	2,034	3450.93 (2410.92, 4490.95)	876.90 (802.95, 950.85)
Female	2,035	3496.06 (2348.90, 4643.23)	884.46 (804.44, 964.48)
Female	2,036	3544.27 (2280.85, 4807.70)	892.13 (805.58, 978.68)
Male	2,022	2943.15 (2717.19, 3169.11)	692.64 (675.45, 709.82)
Male	2,023	2958.19 (2694.31, 3222.06)	697.45 (676.53, 718.38)
Male	2,024	2975.27 (2671.66, 3278.89)	702.51 (678.11, 726.90)
Male	2,025	2994.02 (2648.38, 3339.66)	707.71 (679.97, 735.45)
Male	2,026	3014.26 (2623.96, 3404.57)	712.94 (681.89, 743.99)
Male	2,027	3036.24 (2598.29, 3474.19)	718.21 (683.82, 752.60)
Male	2,028	3060.43 (2571.59, 3549.28)	723.73 (685.91, 761.55)
Male	2,029	3086.67 (2543.43, 3629.92)	729.51 (688.14, 770.88)
Male	2,030	3114.73 (2513.31, 3716.15)	735.46 (690.38, 780.55)
Male	2,031	3144.52 (2480.88, 3808.16)	741.48 (692.48, 790.48)

Sex	Year	ASIR of malaria (95% *CI*)	ASIR of dengue (95% *CI*)
Male	2,032	3176.28 (2446.05, 3906.51)	747.58 (694.43, 800.73)
Male	2,033	3210.42 (2408.90, 4011.94)	754.01 (696.42, 811.60)
Male	2,034	3246.89 (2369.03, 4124.76)	760.79 (698.44, 823.14)
Male	2,035	3285.60 (2325.92, 4245.29)	767.84 (700.38, 835.30)
Male	2,036	3326.58 (2279.15, 4374.01)	775.06 (702.10, 848.02)

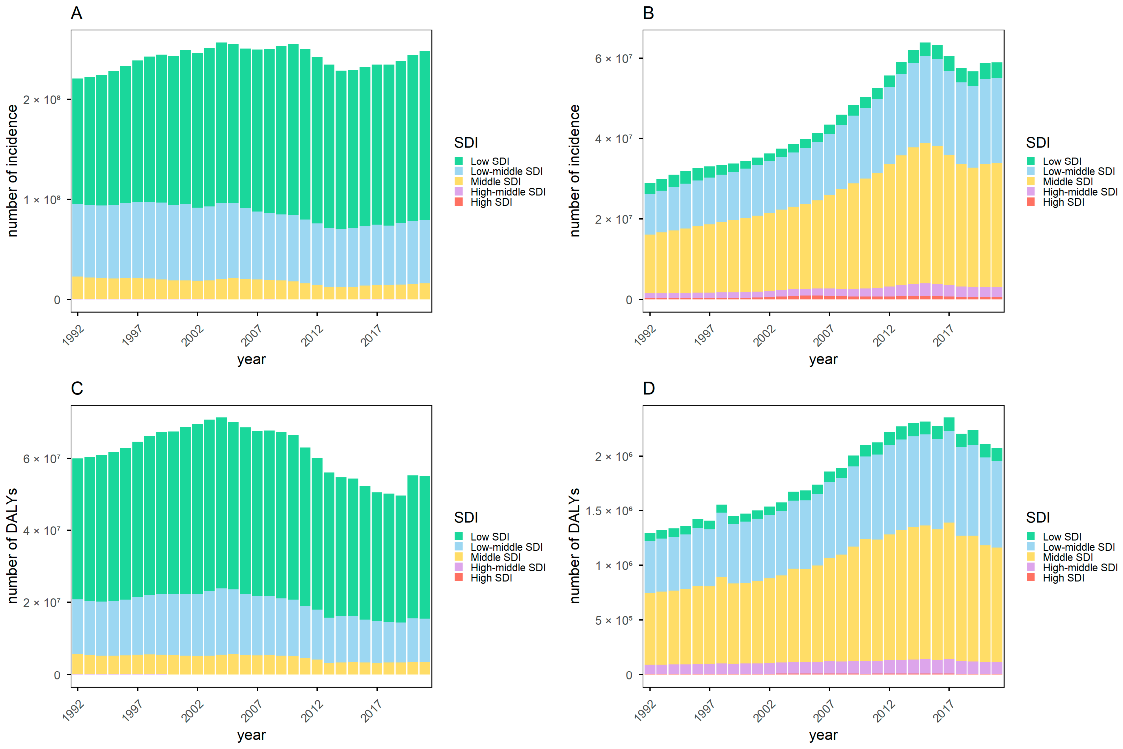

Figure S1. Numbers of incident cases and DALY contributed by five GBD SDI, for malaria and dengue, in 1992-2021

Numbers of incident cases(A) and numbers of DALY(C) of malaria. Numbers of incident cases(B) and numbers of DALY(D) of dengue. DALY=disability-adjusted life-years.

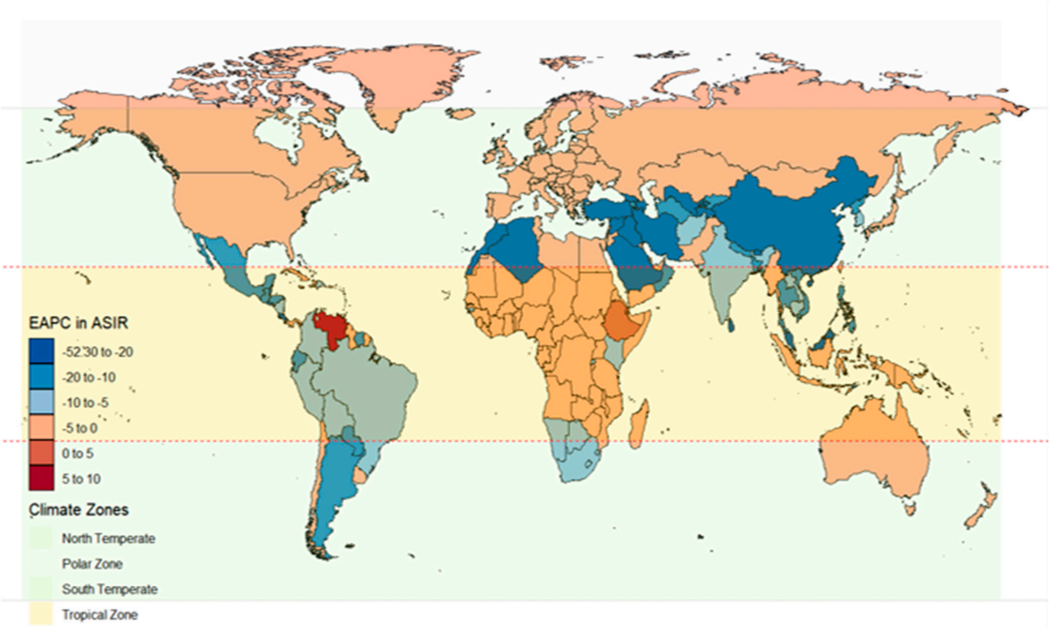

Figure S2. EAPC of ASIR contributed by 204 country, for malaria, in 1992-2021

EAPC=estimated annual percentage change. ASIR= Age-Standardized Incidence Rate.

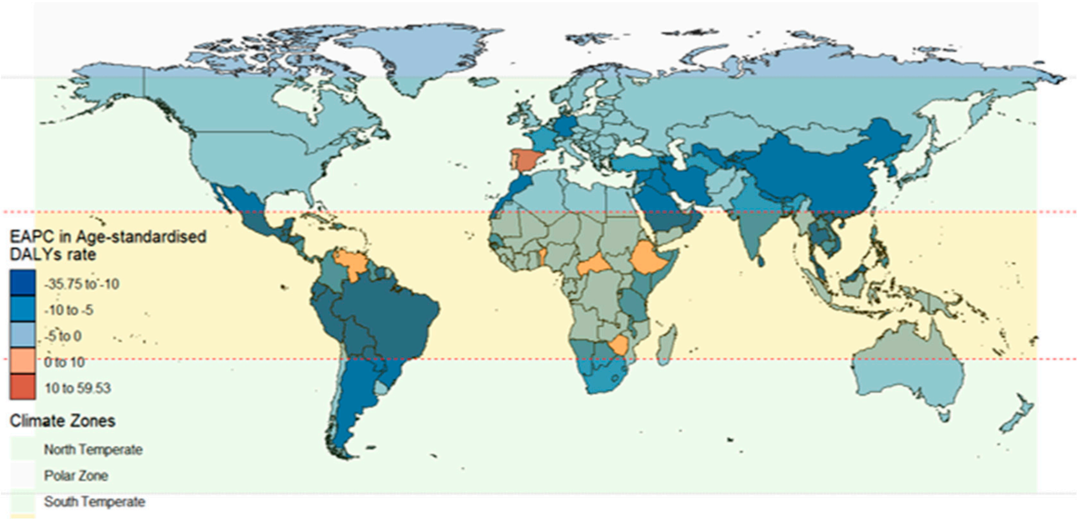

Figure S3. EAPC of age-standardized rates of DALY contributed by 204 country, for malaria, in 1992-2021

EAPC=estimated annual percentage change. DALY=disability-adjusted life-years.

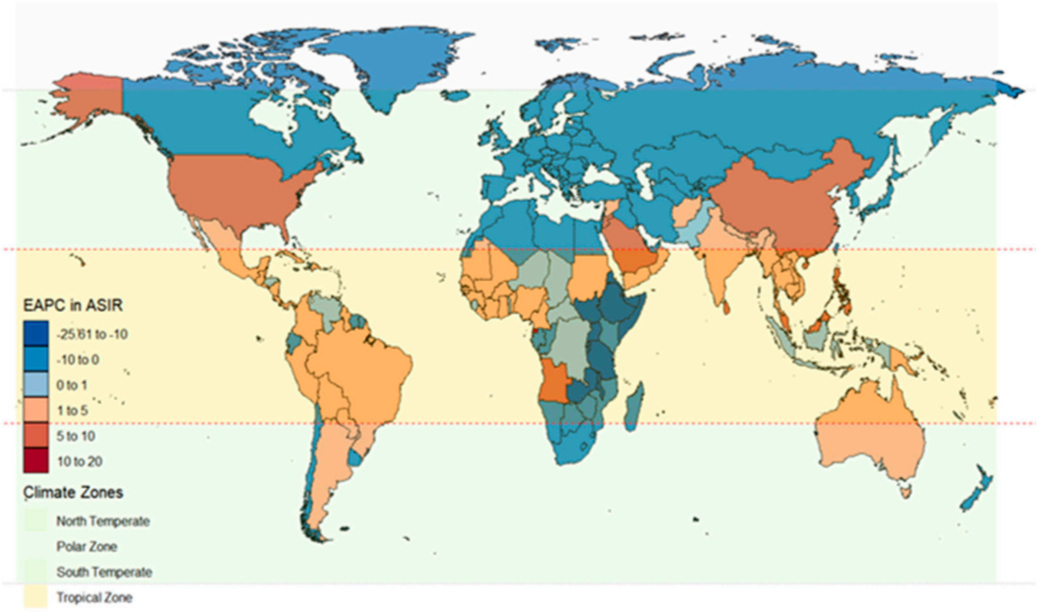

Figure S4. EAPC of ASIR contributed by 204 country, for dengue, in 1992-2021

EAPC=estimated annual percentage change. ASIR= Age-Standardized Incidence Rate.

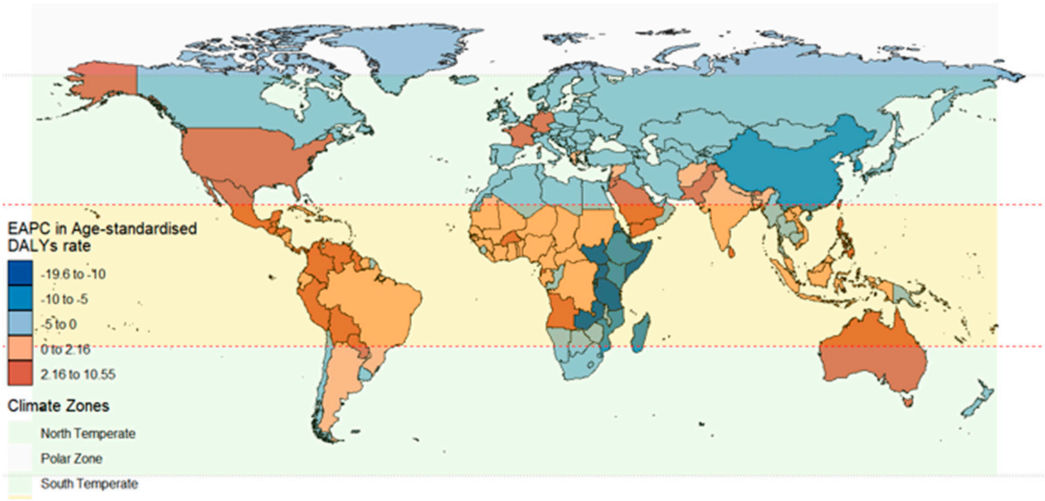

Figure S5. EAPC of age-standardized rates of DALY contributed by 204 country, for dengue, in 1992-2021

EAPC=estimated annual percentage change. DALY=disability-adjusted life-years.

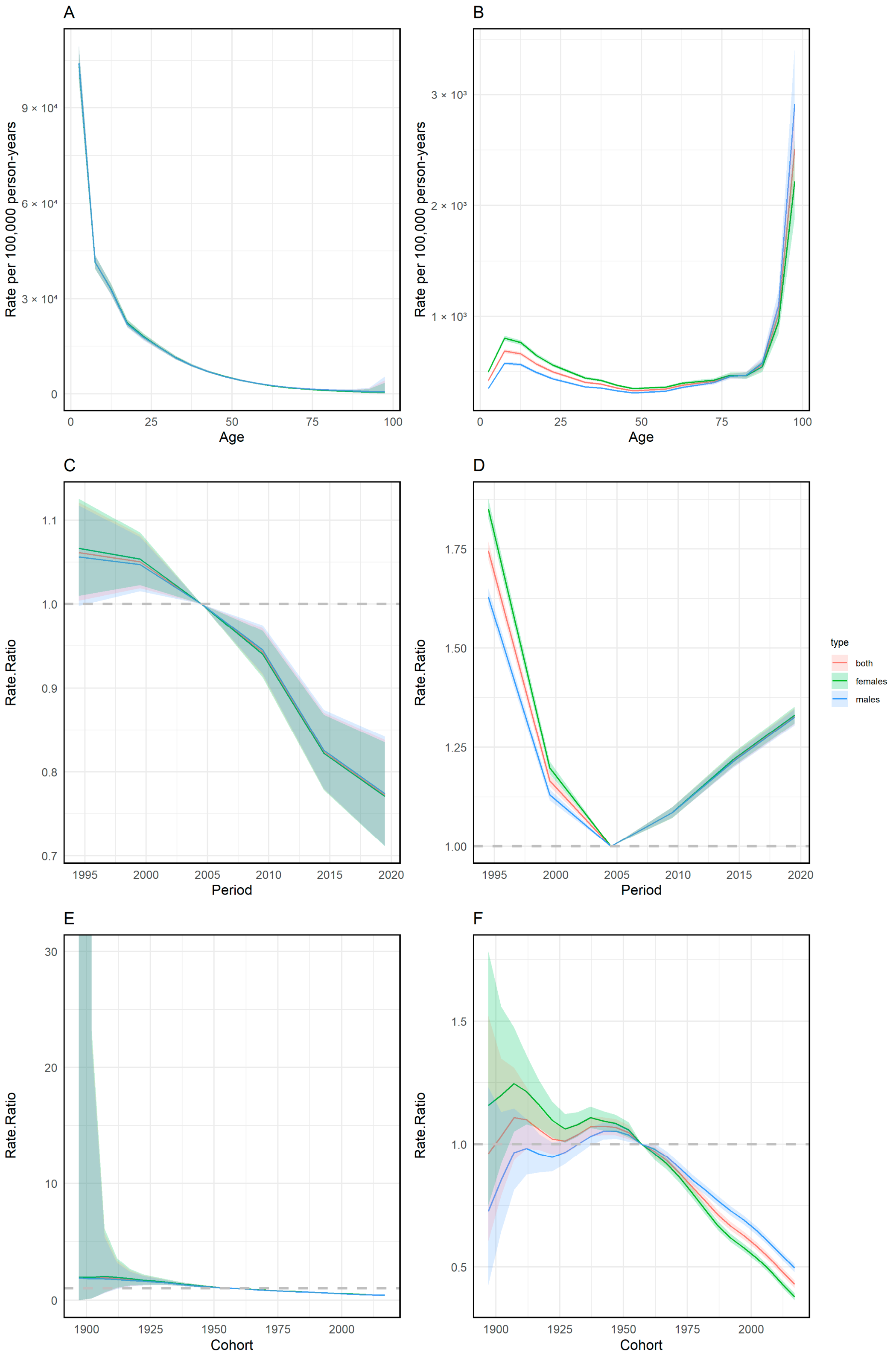

Figure S6. APC effect of incidence rate of malaria and dengue in low SDI region from 1992 to 2021

Age effect of malaria (A) and dengue (B); Period effect of malaria(C) and dengue(D); Cohort effect of malaria(E) and dengue(F). Expected values with 95% CI, based on LSDI Region from 1992 to 2021, are shown as a solid line and shaded area. SDI= low socio-demographic index. The dashed horizontal line indicates RR = 1 (reference).

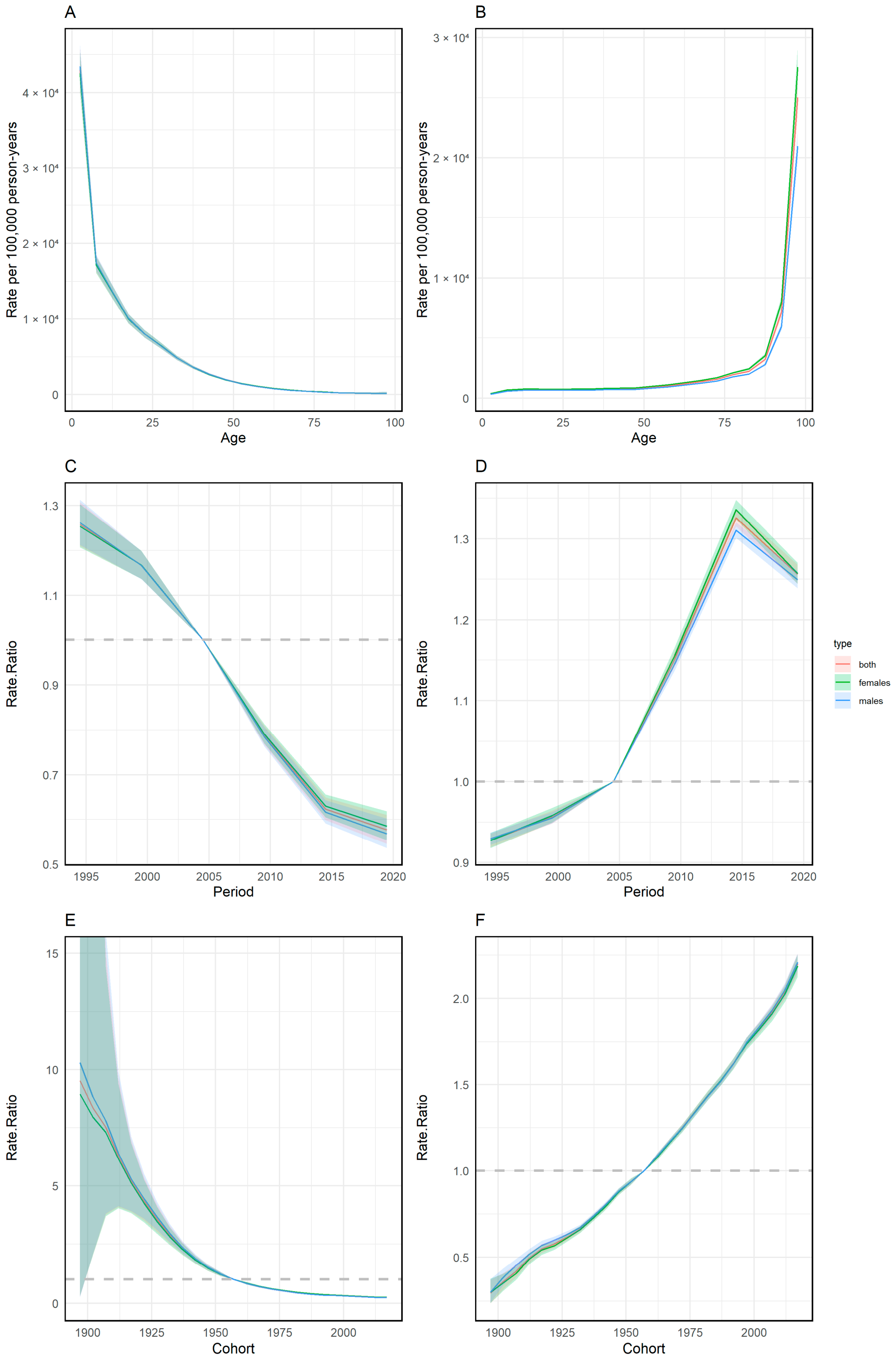

Figure S7. APC effect of incidence rate of malaria and dengue in low-middle SDI region from 1992 to 2021

Age effect of malaria (A) and dengue (B); Period effect of malaria(C) and dengue(D); Cohort effect of malaria(E) and dengue(F). Expected values with 95% CI, based on LSDI Region from 1992 to 2021, are shown as a solid line and shaded area. SDI= socio-demographic index. The dashed horizontal line indicates RR = 1 (reference).

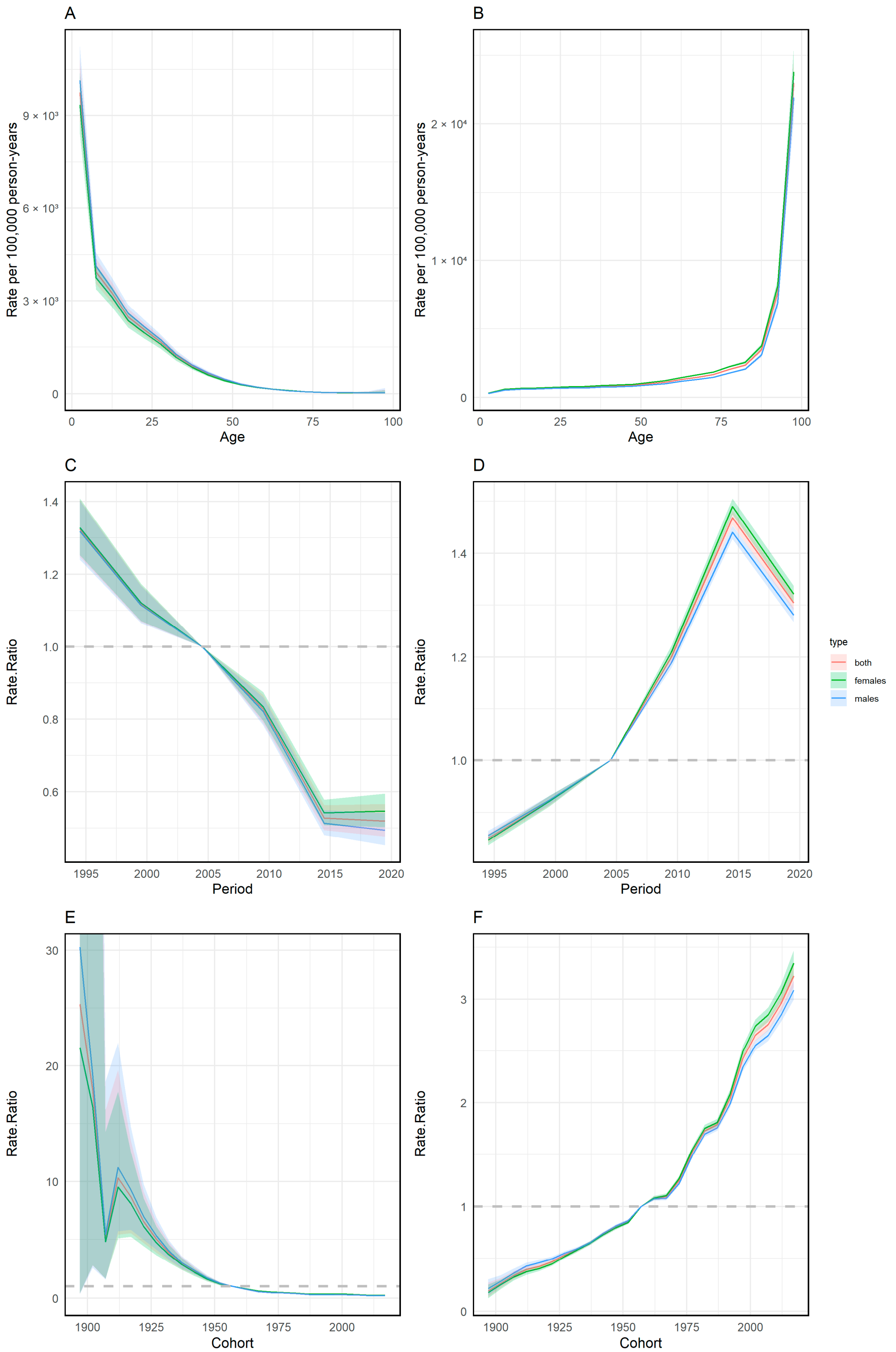

Figure S8. APC effect of incidence rate of malaria and dengue in middle SDI region from 1992 to 2021

Age effect of malaria (A) and dengue (B); Period effect of malaria(C) and dengue(D); Cohort effect of malaria(E) and dengue(F). Expected values with 95% CI, based on LSDI Region from 1992 to 2021, are shown as a solid line and shaded area. SDI= socio-demographic index. The dashed horizontal line indicates RR = 1 (reference).

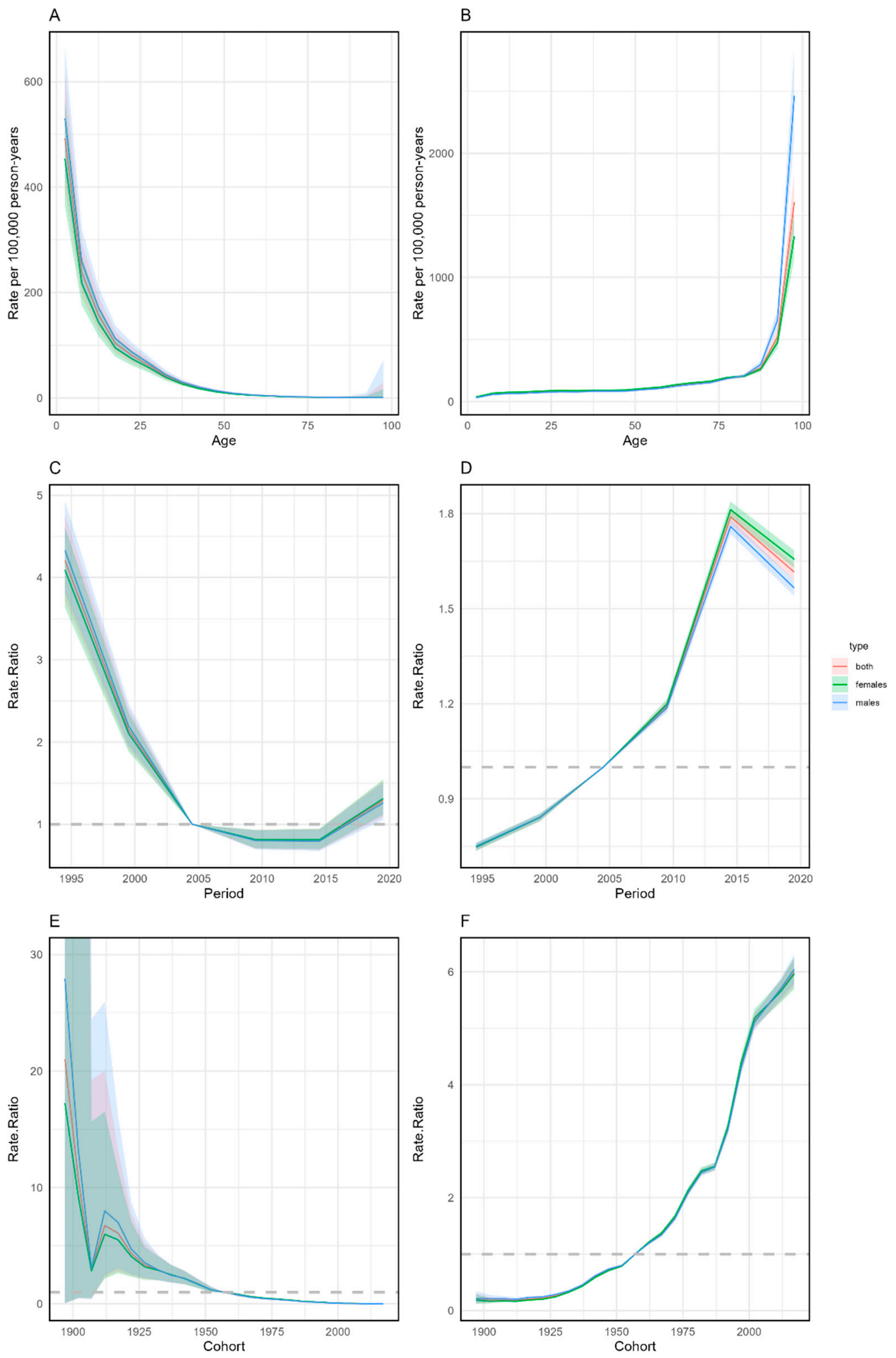

Figure S9. APC effect of incidence rate of malaria and dengue in high-middle SDI region from 1992 to 2021

Age effect of malaria (A) and dengue (B); Period effect of malaria(C) and dengue(D); Cohort effect of malaria(E) and dengue(F). Expected values with 95% CI, based on LSDI Region from 1992 to 2021, are shown as a solid line and shaded area. SDI= socio-demographic index. The dashed horizontal line indicates RR = 1 (reference).

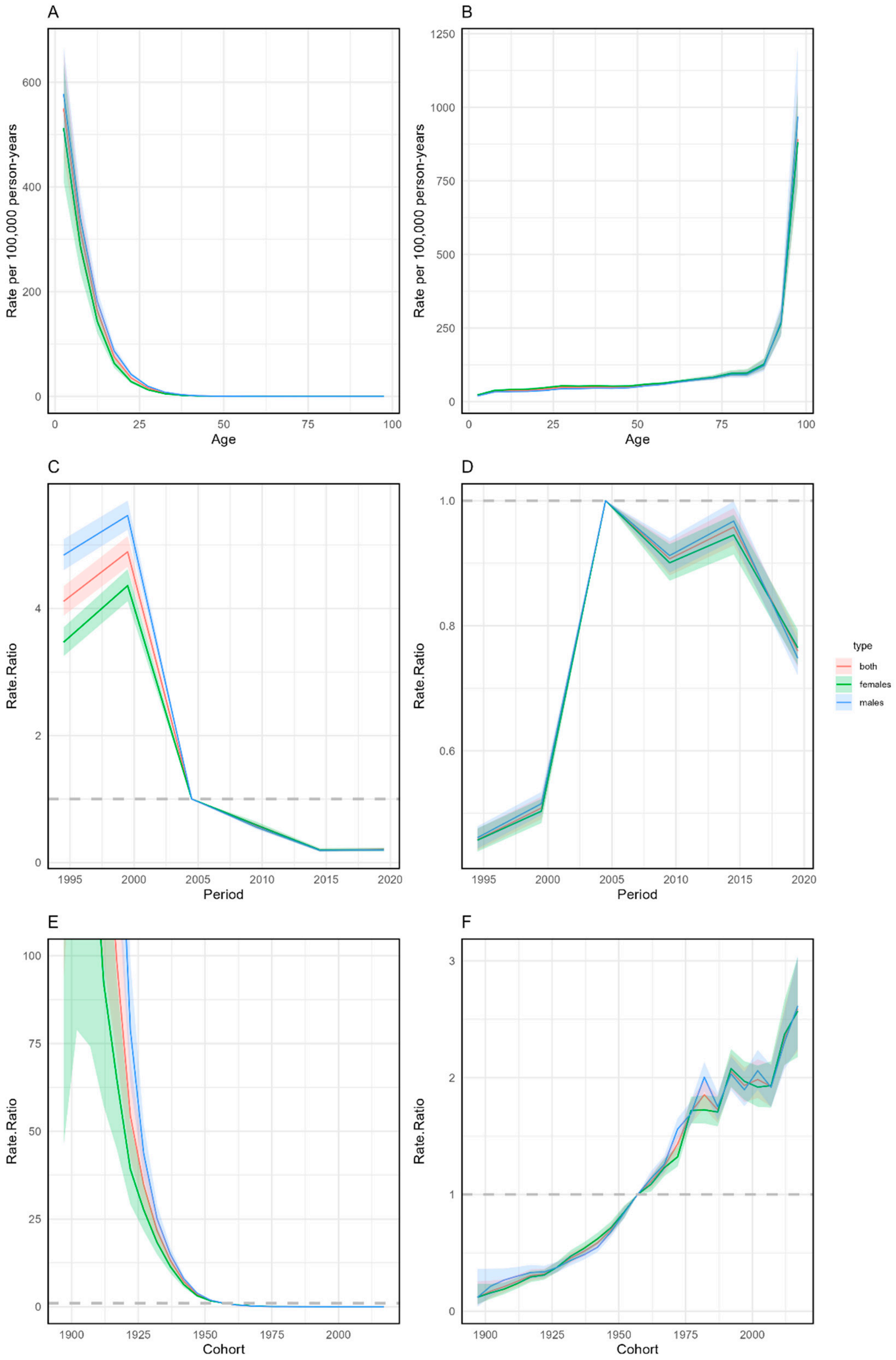

Figure S10. APC effect of incidence rate of malaria and dengue in high SDI region from 1992 to 2021

Age effect of malaria (A) and dengue (B); Period effect of malaria(C) and dengue(D); Cohort effect of malaria(E) and dengue(F). Expected values with 95% CI, based on LSDI Region from 1992 to 2021, are shown as a solid line and shaded area. SDI= socio-demographic index. The dashed horizontal line indicates RR = 1 (reference).

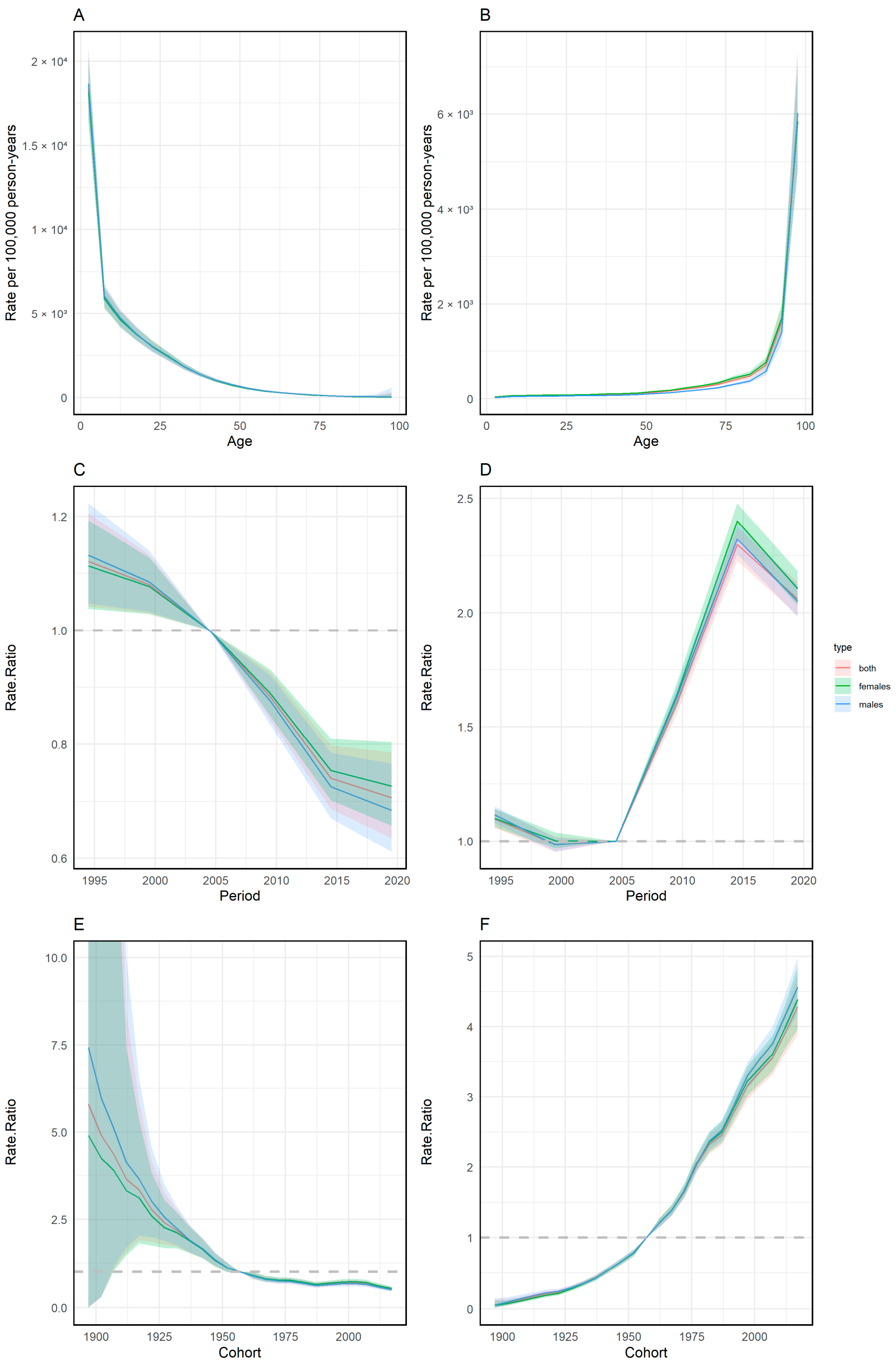

Figure S11. APC sensitivity analysis using GBD lower-bound estimates, global population.

Age effect of malaria (A) and dengue (B); Period effect of malaria(C) and dengue(D); Cohort effect of malaria(E) and dengue(F). Expected values with 95% CI, based on LSDI Region from 1992 to 2021, are shown as a solid line and shaded area. SDI= socio-demographic index. The dashed horizontal line indicates RR = 1 (reference).

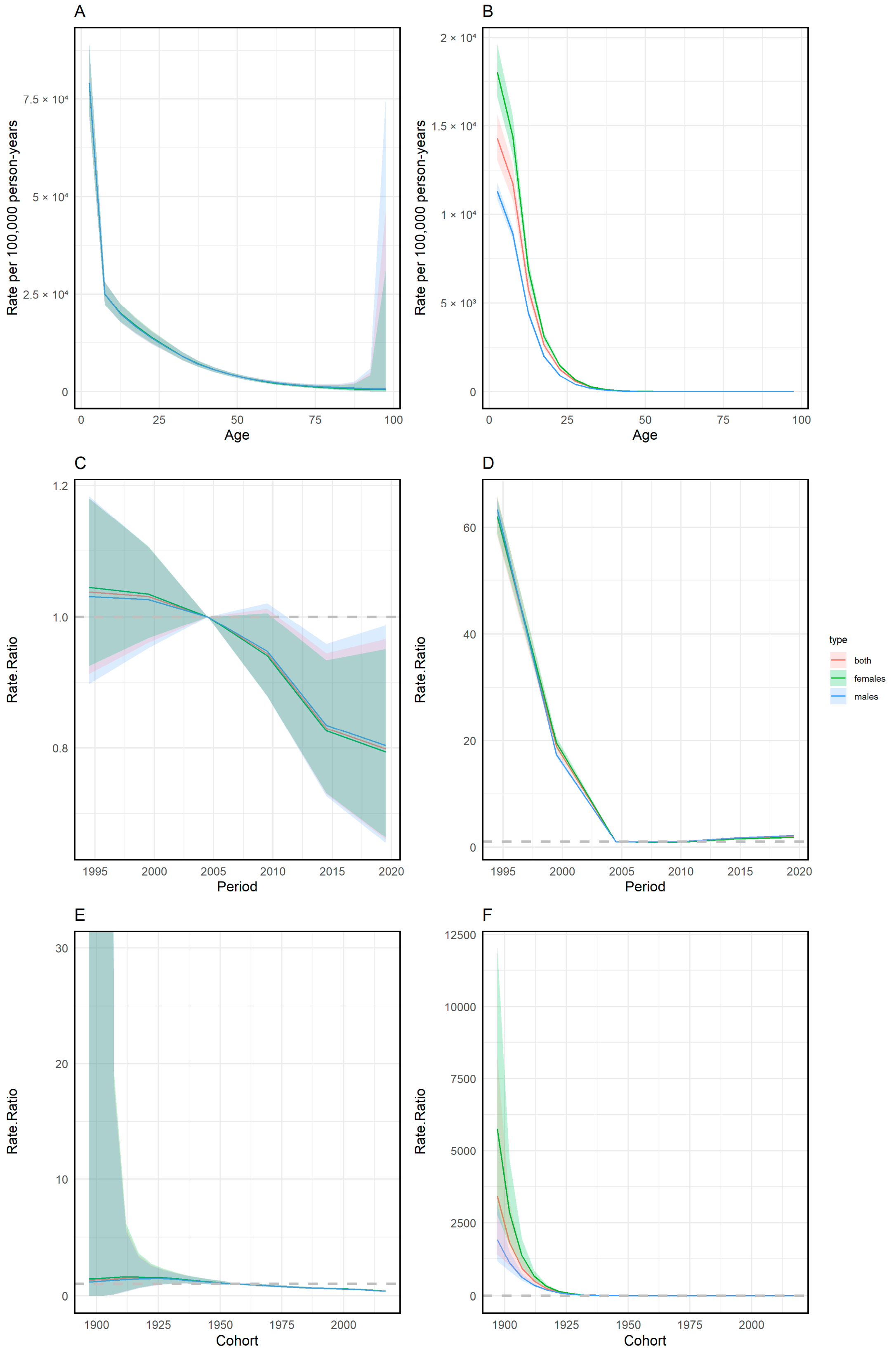

Figure S12. APC sensitivity analysis using GBD lower-bound estimates, low-SDI region.

Age effect of malaria (A) and dengue (B); Period effect of malaria(C) and dengue(D); Cohort effect of malaria(E) and dengue(F). Expected values with 95% CI, based on LSDI Region from 1992 to 2021, are shown as a solid line and shaded area. SDI= socio-demographic index. The dashed horizontal line indicates RR = 1 (reference).

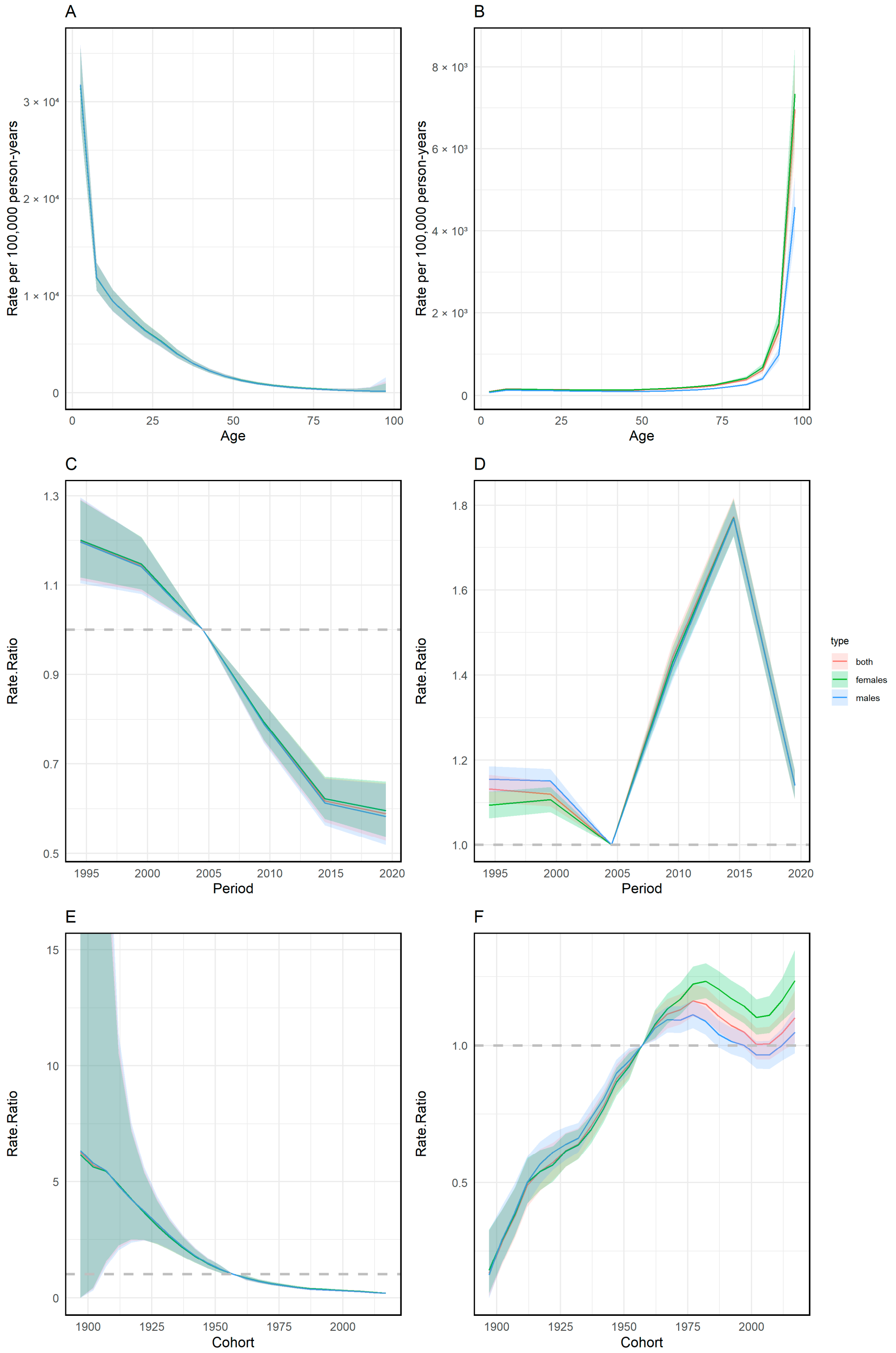

Figure S13. APC sensitivity analysis using GBD lower-bound estimates, low-middle-SDI region. Age effect of malaria (A) and dengue (B); Period effect of malaria(C) and dengue(D); Cohort effect of malaria(E) and dengue(F). Expected values with 95% CI, based on LSDI Region from 1992 to 2021, are shown as a solid line and shaded area. SDI= socio-demographic index. The dashed horizontal line indicates RR = 1 (reference).

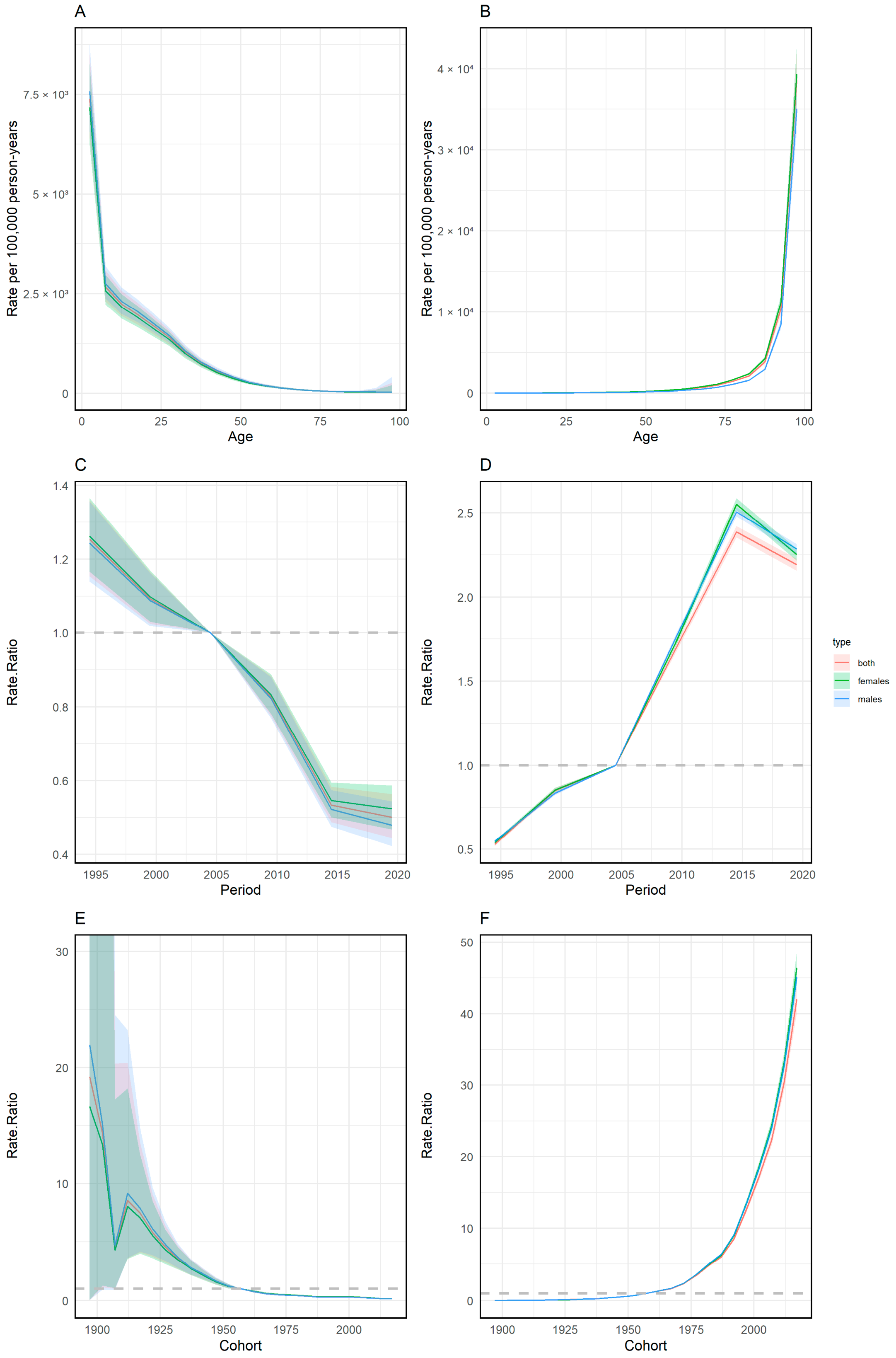

Figure S14. APC sensitivity analysis using GBD lower-bound estimates, middle-SDI region.

Age effect of malaria (A) and dengue (B); Period effect of malaria(C) and dengue(D); Cohort effect of malaria(E) and dengue(F). Expected values with 95% CI, based on LSDI Region from 1992 to 2021, are shown as a solid line and shaded area. SDI= socio-demographic index. The dashed horizontal line indicates RR = 1 (reference).

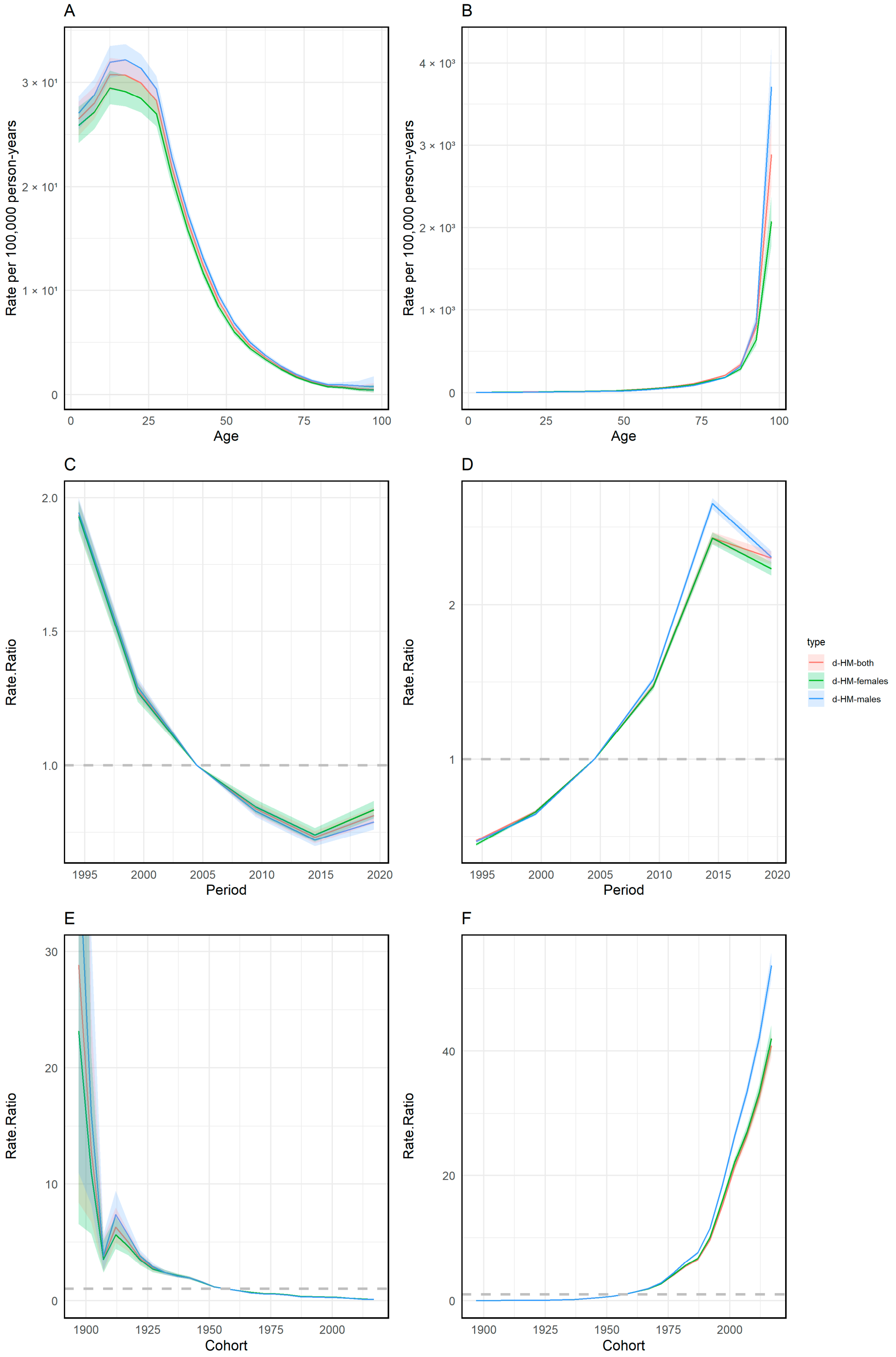

Figure S15. APC sensitivity analysis using GBD lower-bound estimates, high-middle-SDI region. Age effect of malaria (A) and dengue (B); Period effect of malaria(C) and dengue(D); Cohort effect of malaria(E) and dengue(F). Expected values with 95% CI, based on LSDI Region from 1992 to 2021, are shown as a solid line and shaded area. SDI= socio-demographic index. The dashed horizontal line indicates RR = 1 (reference).

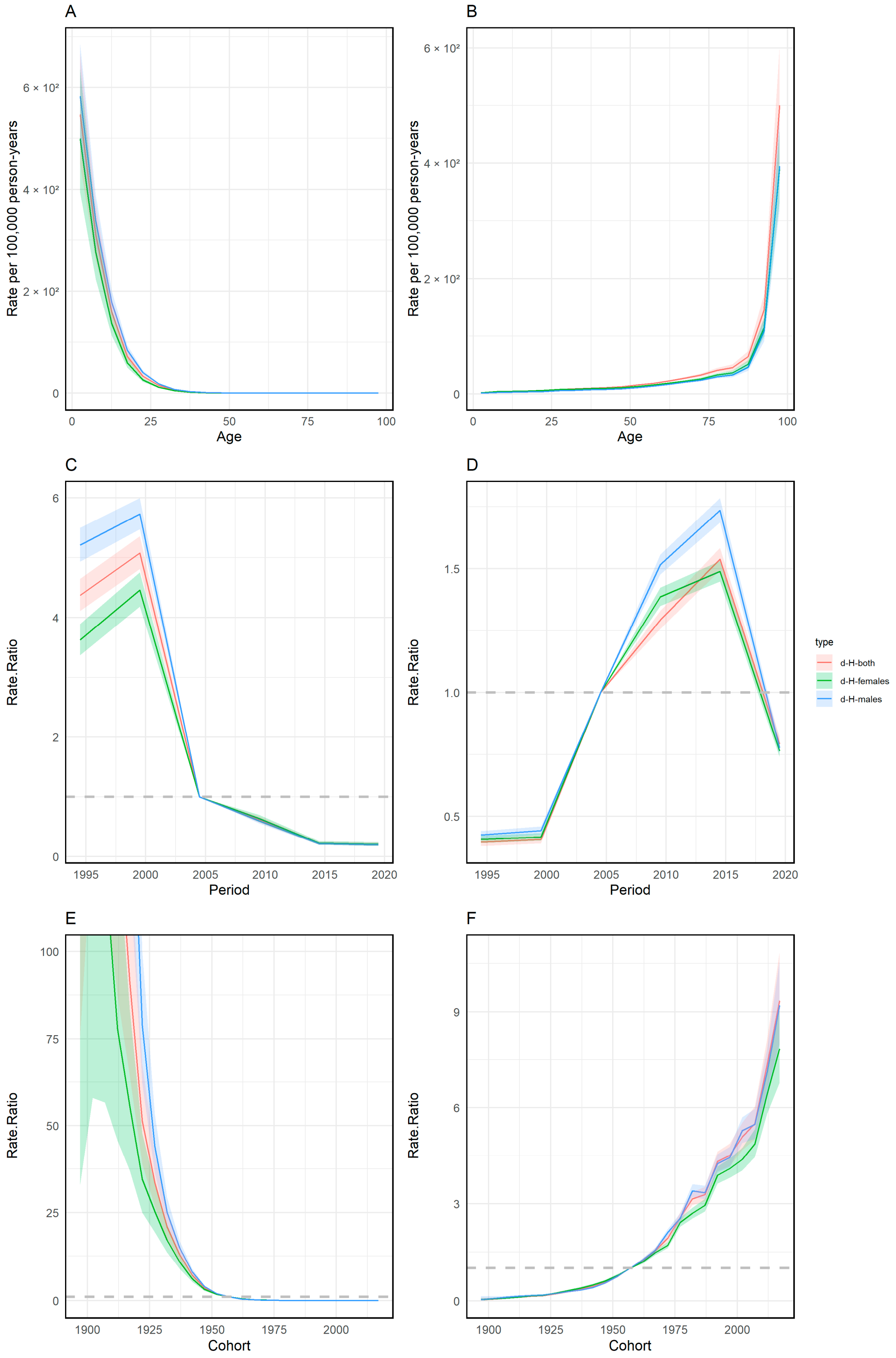

Figure S16. APC sensitivity analysis using GBD lower-bound estimates, high-SDI region.

Age effect of malaria (A) and dengue (B); Period effect of malaria(C) and dengue(D); Cohort effect of malaria(E) and dengue(F). Expected values with 95% CI, based on LSDI Region from 1992 to 2021, are shown as a solid line and shaded area. SDI= socio-demographic index. The dashed horizontal line indicates RR = 1 (reference).

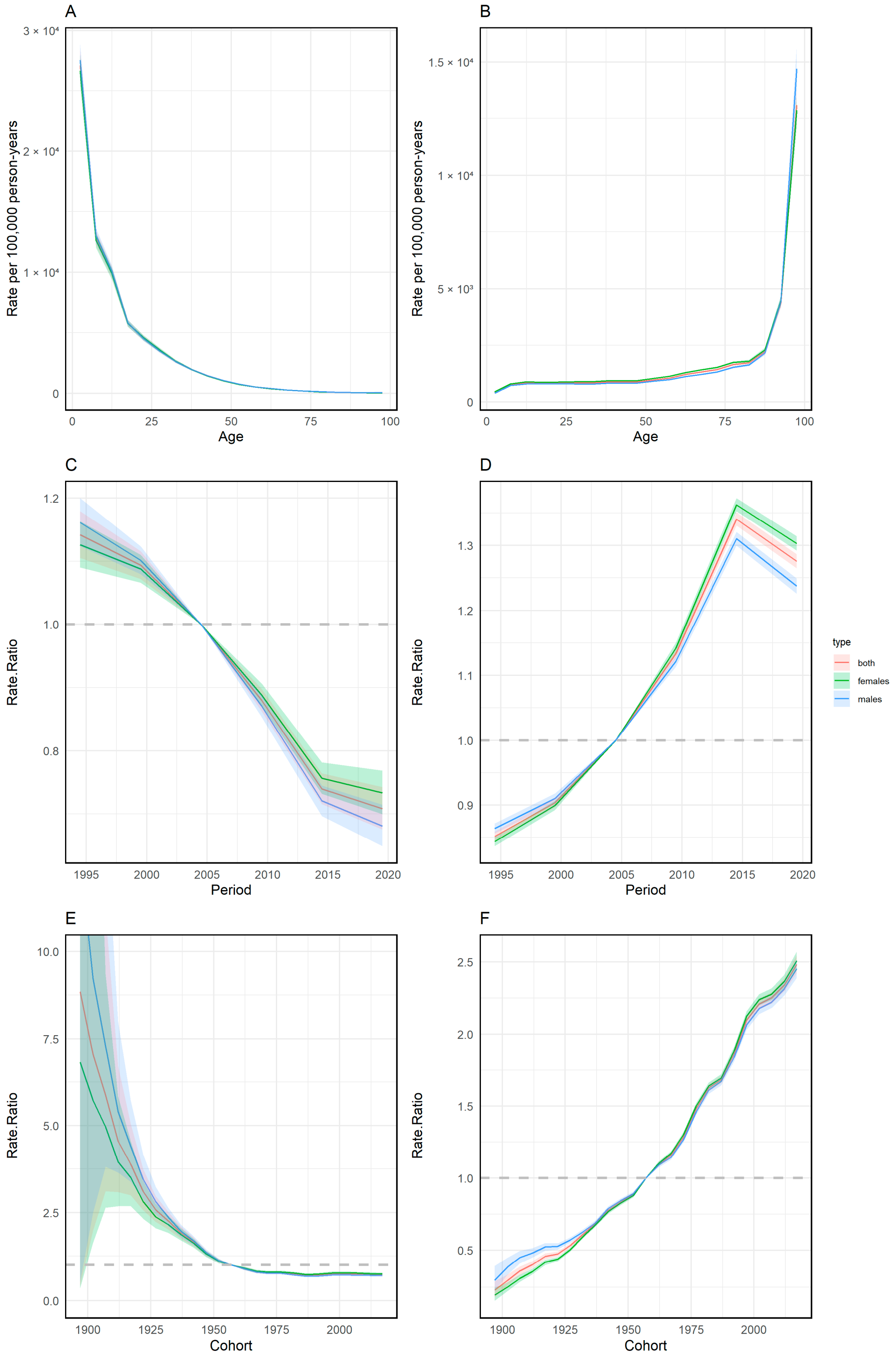

Figure S17. APC sensitivity analysis using GBD upper-bound estimates, global population.

Age effect of malaria (A) and dengue (B); Period effect of malaria(C) and dengue(D); Cohort effect of malaria(E) and dengue(F). Expected values with 95% CI, based on LSDI Region from 1992 to 2021, are shown as a solid line and shaded area. SDI= socio-demographic index. The dashed horizontal line indicates RR = 1 (reference).

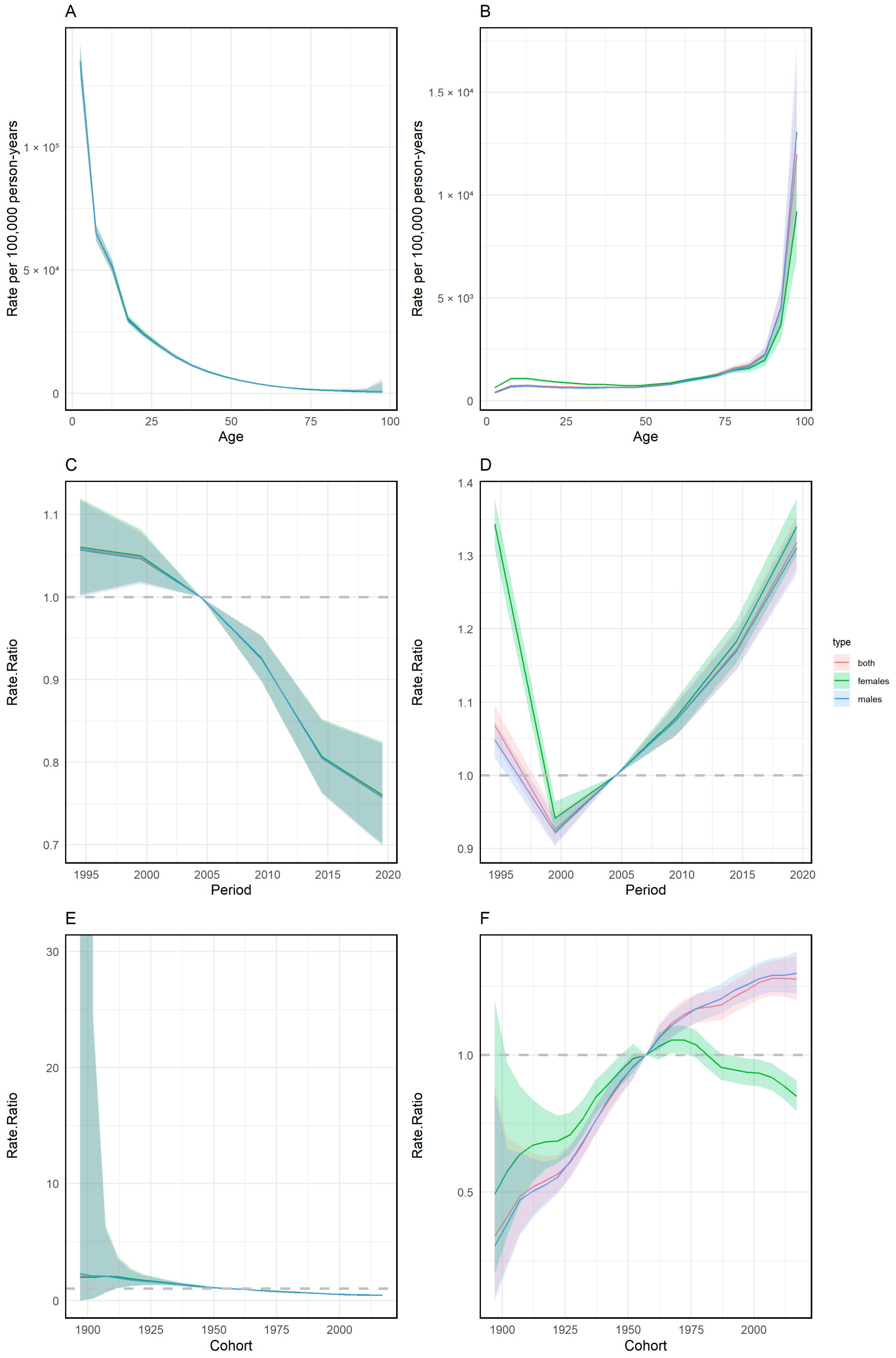

Figure S18. APC sensitivity analysis using GBD upper-bound estimates, low-SDI region.

Age effect of malaria (A) and dengue (B); Period effect of malaria(C) and dengue(D); Cohort effect of malaria(E) and dengue(F). Expected values with 95% CI, based on LSDI Region from 1992 to 2021, are shown as a solid line and shaded area. SDI= socio-demographic index. The dashed horizontal line indicates RR = 1 (reference).

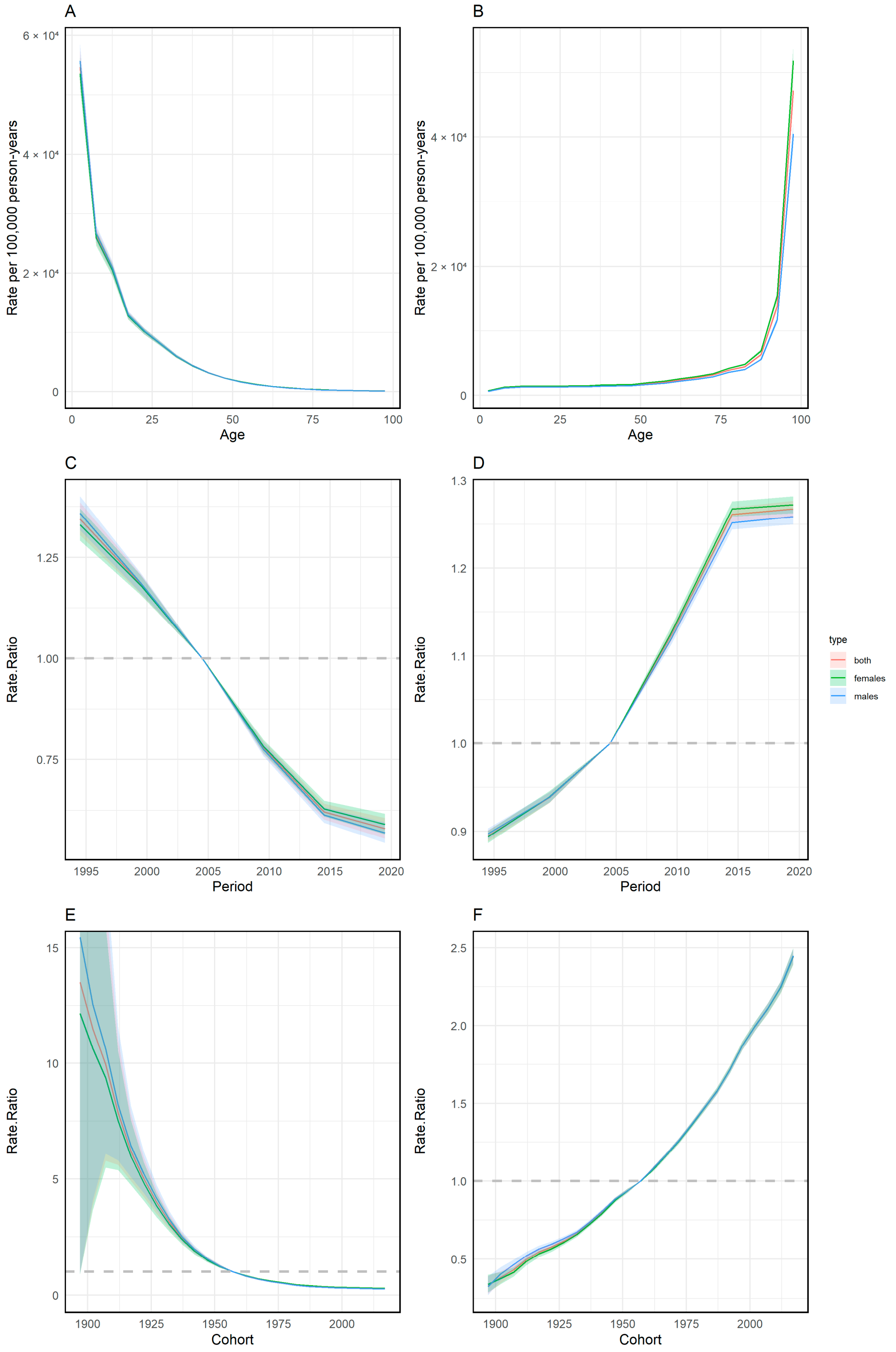

Figure S19. APC sensitivity analysis using GBD upper-bound estimates, low-middle-SDI region. Age effect of malaria (A) and dengue (B); Period effect of malaria(C) and dengue(D); Cohort effect of malaria(E) and dengue(F). Expected values with 95% CI, based on LSDI Region from 1992 to 2021, are shown as a solid line and shaded area. SDI= socio-demographic index. The dashed horizontal line indicates RR = 1 (reference).

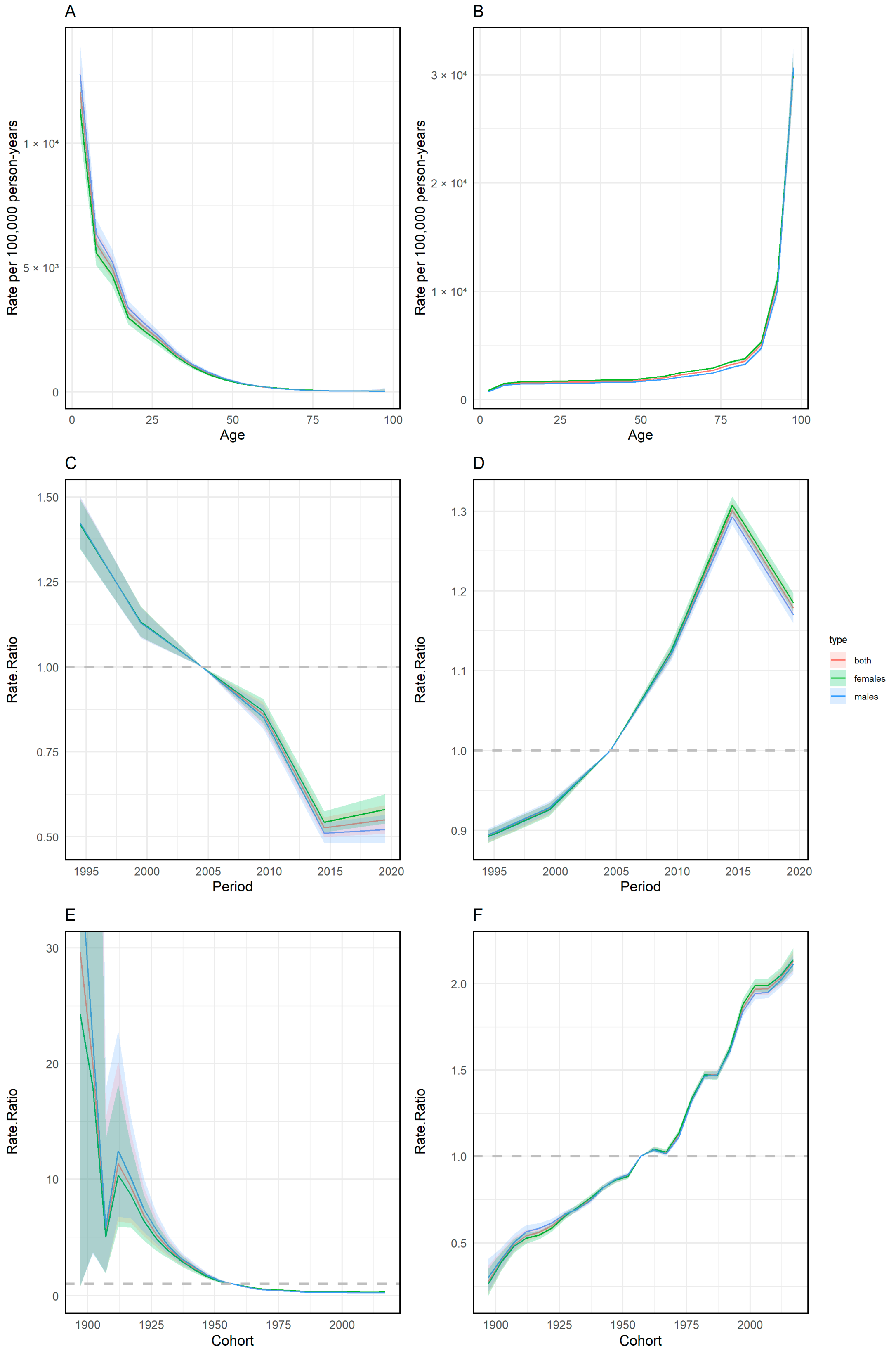

Figure S20. APC sensitivity analysis using GBD upper-bound estimates, middle-SDI region.

Age effect of malaria (A) and dengue (B); Period effect of malaria(C) and dengue(D); Cohort effect of malaria(E) and dengue(F). Expected values with 95% CI, based on LSDI Region from 1992 to 2021, are shown as a solid line and shaded area. SDI= socio-demographic index. The dashed horizontal line indicates RR = 1 (reference).

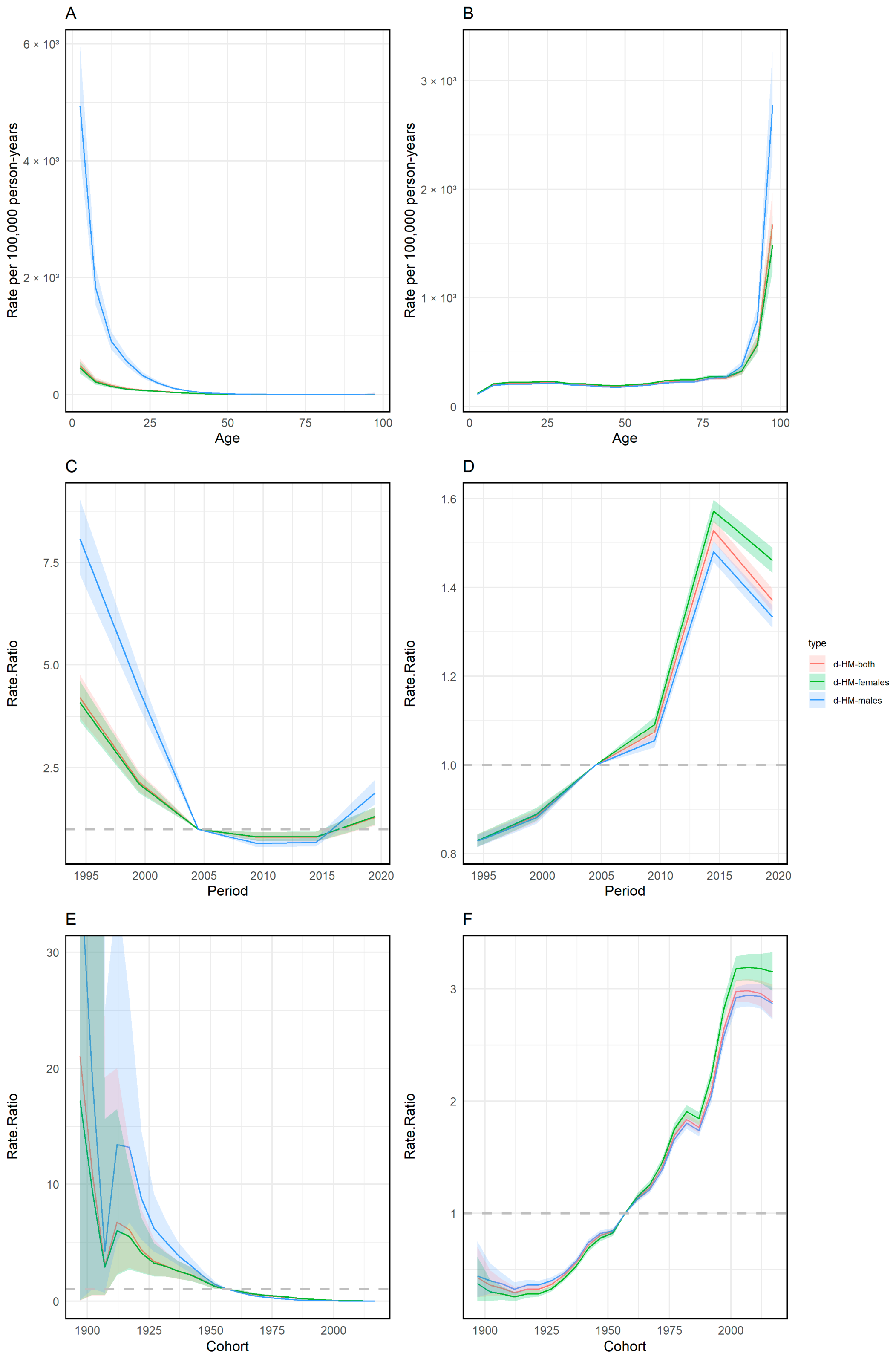

Figure S21. APC sensitivity analysis using GBD upper-bound estimates, high-middle-SDI region. Age effect of malaria (A) and dengue (B); Period effect of malaria(C) and dengue(D); Cohort effect of malaria(E) and dengue(F). Expected values with 95% CI, based on LSDI Region from 1992 to 2021, are shown as a solid line and shaded area. SDI= socio-demographic index. The dashed horizontal line indicates RR = 1 (reference).

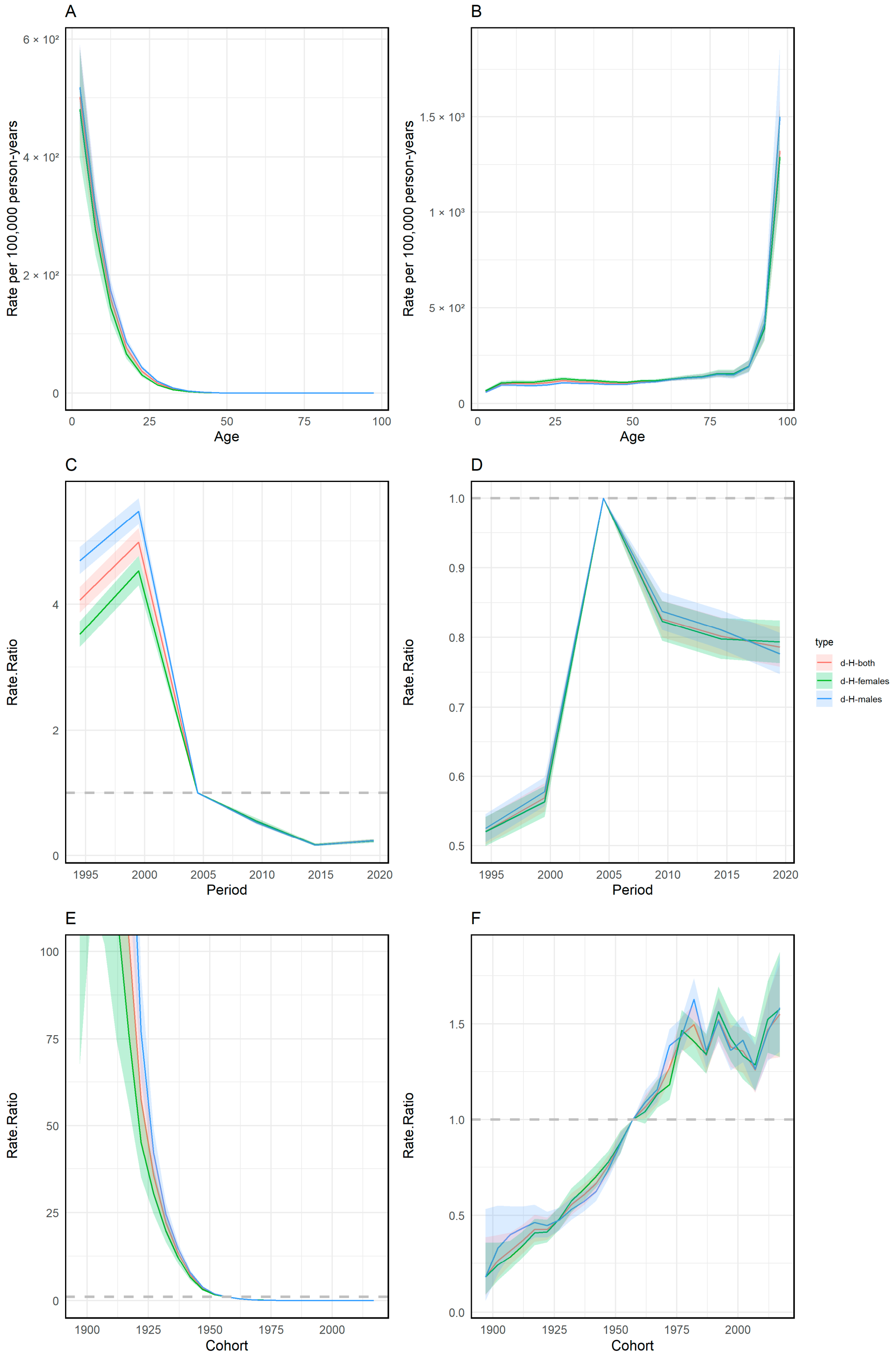

Figure S22. APC sensitivity analysis using GBD upper-bound estimates, high-SDI region.

Age effect of malaria (A) and dengue (B); Period effect of malaria(C) and dengue(D); Cohort effect of malaria(E) and dengue(F). Expected values with 95% CI, based on LSDI Region from 1992 to 2021, are shown as a solid line and shaded area. SDI= socio-demographic index. The dashed horizontal line indicates RR = 1 (reference).

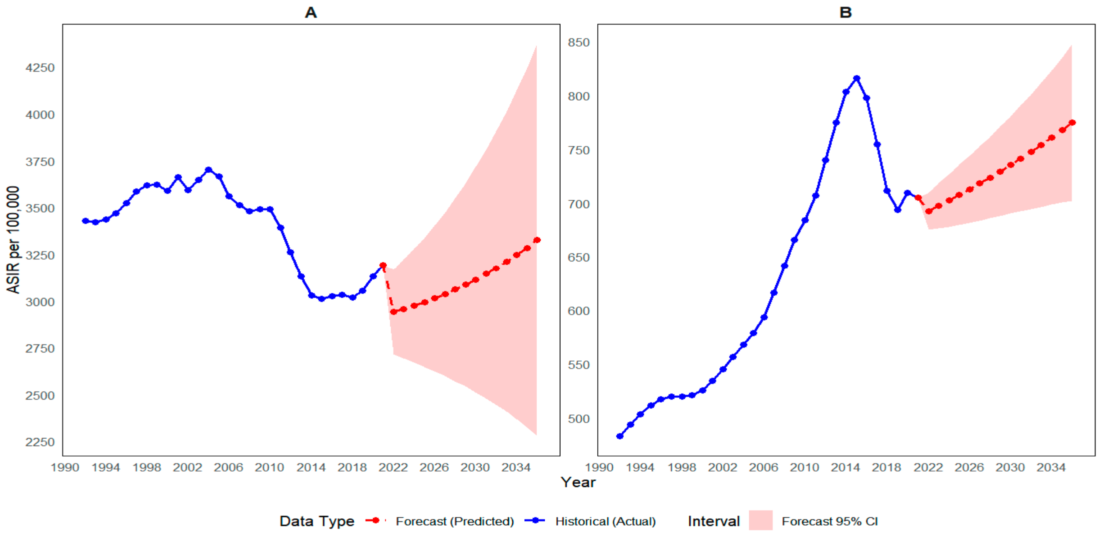

Figure S23. Prediction of age-standardized incidence rates of malaria and dengue by males in global from 1992 to 2036

The solid lines indicate the observed values (1992–2021) and the dotted lines are the predicted values (2022–2036). The ASIR of malaria(A) and dengue(B) globally. ASIR = Age-Standardized Incidence Rate.

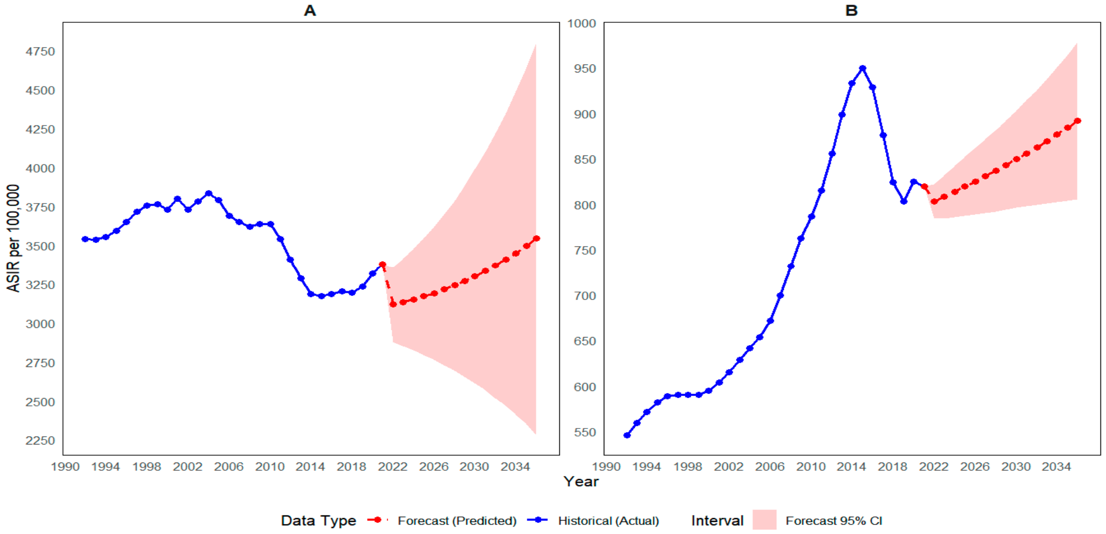

Figure S24. Prediction of age-standardized incidence rates of malaria and dengue by females in global from 1992 to 2036

The solid lines indicate the observed values (1992–2021) and the dotted lines are the predicted values (2022–2036). The ASIR of malaria(A) and dengue(B) globally. ASIR = Age-Standardized Incidence Rate.
